# Integrated Genetic Analysis of Racial Differences of Common *GBA* Variants in Parkinson's Disease: A Meta-Analysis

**DOI:** 10.3389/fnmol.2018.00043

**Published:** 2018-02-15

**Authors:** Yuan Zhang, Li Shu, Qiying Sun, Xun Zhou, Hongxu Pan, Jifeng Guo, Beisha Tang

**Affiliations:** ^1^Department of Neurology, Xiangya Hospital, Central South University, Changsha, China; ^2^Department of Geriatrics, Xiangya Hospital, Central South University, Changsha, China; ^3^National Clinical Research Center for Geriatric Disorders, Changsha, China; ^4^Key Laboratory of Hunan Province in Neurodegenerative Disorders, Central South University, Changsha, China; ^5^Center for Medical Genetics, School of Life Sciences, Central South University, Changsha, China; ^6^Parkinson's Disease Center of Beijing Institute for Brain Disorders, Beijing, China

**Keywords:** Parkinson's disease, *GBA*, AJ, non-AJ, meta-analysis

## Abstract

**Background:** Numerous studies have indicated that there is a possible relationship between *GBA* variants and Parkinson's disease (PD), however, most of them focused on a few variants such as L444P, N370S. We performed a comprehensive pooled analysis to clarify the relationship between variations of *GBA* and the risk of PD in different racial groups.

**Methods**: Standard meta-analysis was conducted, including generating inclusion and exclusion criteria, searching literature, extracting and analyzing data.

**Results**: Fifty studies containing 20,267 PD patients and 24,807 controls were included. We found that variants 84insGG, IVS2+1G>A, R120W, H255Q, E326K, T369M, N370S, D409H, L444P, R496H and RecNciI increased the risk of PD in total populations (OR: 1.78–10.49; *p*: <0.00001, 0.00005, 0.0008, 0.005, <0.00001, 0.004, <0.00001, 0.0003, <0.00001, <0.0001, 0.0001). In subgroup analysis by ethnicity, in AJ populations, variants 84insGG, R496H, N370S increased the risk of PD (OR: 9.26–3.51; *p*: <0.00001, <0.0001, <0.00001). In total non-AJ populations, variants L444P, R120W, IVS2+1G>A, H255Q, N370S, D409H, RecNciI, E326K, T369M increased the risk of PD (OR: 8.66–1.89; *p*: <0.00001, 0.0008, 0.02, 0.005, <0.00001, 0.001, 0.0001, <0.00001, 0.002). Among the non-AJ populations, pooled analysis from five different groups were done separately. Variants L444P, N370S, H255Q, D409H, RecNciI, E326K increased risk of PD (OR: 6.52–1.84; *p*: <0.00001, <0.00001, 0.005, 0.005, 0.04, <0.00001) in European/West Asians while R120W and RecNciI in East Asians (OR: 14.93, 3.56; *p*: 0.001, 0.003). L444P increased the risk of PD in Hispanics, East Asians and Mixed populations (OR: 15.44, 12.43, 7.33; *p*: 0.00004, <0.00001, 0.009). Lacking of enough original studies, we failed to conduct quantitative analysis in Africa.

**Conclusions**: Obvious racial differences were found for *GBA* variants in PD. 84insGG and R496H exclusively increased PD risks in AJ populations, so did L444P, R120W, IVS2+1G>A, H255Q, D409H, RecNciI, E326K, T369M in non-AJ populations. N370S increased the risk of PD in both ethnics. In non-AJ subgroup populations, N370S, H255Q, D409H, E326K exclusively increased PD risks in European/West Asians, as were R120W in East Asians. L444P increased the risk of PD in all groups in non-AJ ethnicity. These results will contribute to the future genetic screening of *GBA* gene in PD.

## Introduction

Parkinson's disease (PD), one of the most common progressive neurodegenerative disorders, is characterized by motor and non-motor symptoms. Although the etiology of PD is still unclear, it is convincing that the interaction of genetic factor, environmental factor and aging together contribute to the disease (Kalia and Lang, 2015).

*GBA* encodes lysosomal enzyme glucocerebrosidase (GCase), the homozygous mutations of which can lead to accumulation of glucocerebroside and storage of lysosomal lipid, thus causing Gaucher's disease (GD) (Grabowski, 1993). More than 300 mutations including point mutations, insertions, deletions and frameshift mutations were discovered as the pathogenic variants of GD nowadays (O'Regan et al., 2017). In the year 2004, Lwin *et.al* firstly identified a higher rate of *GBA* mutations, either heterozygous or homozygous (N370S, L444P, K198T, and R329C), in brain samples from PD patients, which indicated a possible relationship between *GBA* mutations and the disease (Lwin et al., 2004). Since then, numbers of studies have confirmed that G*BA* mutations were associated with PD risks (Aharon-Peretz et al., 2004; Clark et al., 2005).

A prominent genetic feature of *GBA* mutations in PD was ethnic heterogeneity in different regions. Several meta-analyses (Mao et al., 2013; Chen et al., 2014; Zhao et al., 2016) have demonstrated that *GBA* variants like L444P and N370S are risk factors for PD. However, pooled analysis of the association between other *GBA* variants and the risk of PD is still lacking. And the few available original studies investigating associations between *GBA* variants, such as 84insGG, R120W, and R496H, and the risk of PD yielded inconsistent results. Thus, we performed a comprehensive meta-analysis to clarify the relationship between the potential variations of *GBA* and the risk of PD in total populations from different ethnicities, which would be essential in genetic screening in PD patients from different populations with different ethnic background.

## Materials and methods

### Inclusion criteria

We performed our meta-analysis based on PICOS (participants, interventions, controls, outcomes, and studies) rules.

Participants: all PD patients were diagnosed with any accepted criteria without the requirement negative of family history.

Interventions: the DNA from peripheral blood of all cases and controls were analyzed using PCR based methods or other accepted methods.

Controls: all controls were reported healthy controls or had no PD or other neurological disorders.

Outcomes: the number of cases or controls carrying homozygous or heterozygous variants of *GBA* was reported (unless articles pointing out that there were homozygous variants of *GBA* screened out in PD patients, we considered the variants of *GBA* as heterozygous).

Studies: types of all studies were case-control study or cohort study.

### Literature search

With the key words of (“parkinson^*^” or “PD”) and (“*GBA*” or “glucocerebrosidase”), we searched the electronic databases including Pubmed, embase, web of science (WOS) and the Cochrane library for English publications up to October 20th, 2017. All searched papers were imported into ENDNOTE for further management. One copy of the overlapping articles from different databases was kept with the help of electronic and manual checking. Two researchers performed the search independently. If there were controversial ideas, the third researcher was asked to solve the debates.

### Data extraction

For studies satisfying the aforesaid criteria, two authors independently extracted the following data: year of publication, first author's name, countries, ethnicities, numbers of PD patients and controls, sequencing strategy (all *GBA* exons or specific variants), the sequencing results of specific variants' genotypes and alleles in PD patients and controls. Complete data were independently extracted by two researchers. Due the prominent ethnic heterogeneity, especially in AJ and non-AJ populations (Sidransky et al., 2009; Sun et al., 2010), of *GBA* mutations in PD in different regions, all ethnicities information including AJ, non-AJ (Africans, European/West Asians, Hispanics, East Asians, Mixed:composed of at least two different groups) (Risch et al., 2002), were extracted according the original studies. Once confronted with debates or difficulties, the third researcher was asked to make the decision. Newcastle-Ottawa Scale (NOS) (Stang, 2010), a good tool to assess the quality of case-control study from the aspects of selection, comparability and exposure, was used to assess the quality of all included case-control studies.

### Statistical analysis

Revman 5.3 software was used to do all the statistical analysis. Pooled odds ratio (OR) and 95% CI (confidence interval) were calculated to assess the strength of association between the variants and PD. Heterogeneity across each study was identified by a standard Q test. Q statistic (*P* < 0.1) and *I*^2^ statistic (>50%) indicated heterogeneity of the analysis. If the heterogeneity was not significant (*P* > 0.1, *I*^2^ ≤ 50%), a fixed-effects model (FM) was used to do the analysis. Otherwise a random-effects model (RM) was applied. Reporting biases were measured by funnel plot analysis. In order to measure the stability of the analysis, sensitivity analysis was performed by removing each individual study in turn from the total and re-analyzing the results from the remainder.

## Results

**Figure 1:**
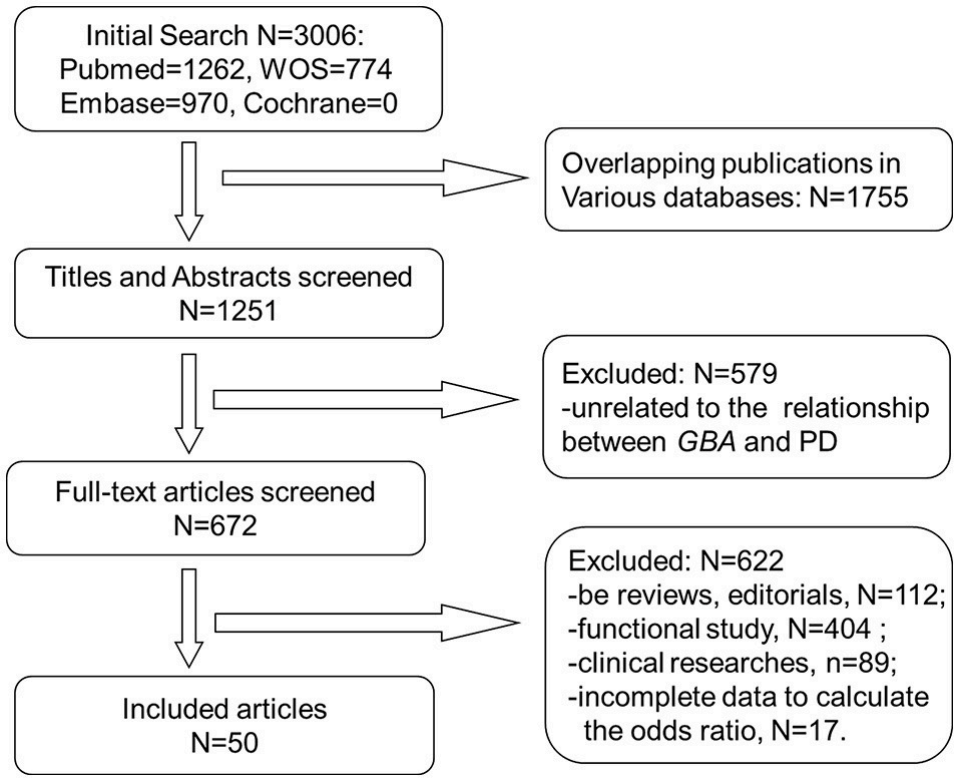
Flow chart of publications included process.

A total of 3,006 publications were retrieved from all searched databases and finally 50 studies were included for further analysis (Figure 1). The characteristics of all included studies, containing 20,267 PD patients and 24,807 controls, were shown on Table 1. The NOS scores of each study indicated that all of them were of good quality.

**Table 1:** The characteristics of all publications included.

**References**	**Groups**	**Area**	**Seq. all *GBA* Exs**	**Total no. of**	**NOS**
				**PD/Controls**	
**AJ**					
Aharon-Peretz et al., 2004	AJ	Israel	NO	99/1543	9
Clark et al., 2005	AJ	America	NO	160/92	9
Clark et al., 2007	AJ	America	YES	178/85	9
Gan-Or et al., 2008	AJ	Israel	NO	420/4138	8
Dagan et al., 2015	AJ	Israel	NO	287/400	8
**NON-AJ**					
Tan et al., 2007	East Asians	Singapore	NO	331/347	8
Ziegler et al., 2007	East Asians	Taiwan	YES	92/92	9
Wu et al., 2007	East Asians	Taiwan	NO	518/339	8
Gutti et al., 2008	East Asians	Taiwan	NO	184/92	7
Mitsui et al., 2009	East Asians	Japan	YES	534/544	9
Hu et al., 2010	East Asians	China	NO	328/300	8
Sun et al., 2010	East Asians	China	NO	402/413	8
Mao et al., 2010	East Asians	China	NO	616/411	8
Huang et al., 2011	East Asians	China	NO	967/780	8
Choi et al., 2012	East Asians	Korea	YES	277/100	9
Zhang et al., 2012	East Asians	China	NO	195/443	7
Wang et al., 2012	East Asians	China	NO	208/298	8
Pulkes et al., 2014	East Asians	Thailand	YES	480/395	9
Li et al., 2014	East Asians	Japan	YES	147/100	9
Yu et al., 2015	East Asians	China	YES	184/130	9
Guo et al., 2015	East Asians	China	NO	1019/1030	8
Toft et al., 2006	European/West Asians	Norway	NO	311/474	8
De Marco et al., 2008	European/West Asians	Italy	NO	395/483	8
Bras et al., 2009	European/West Asians	Portugal	YES	230/430	9
Mata et al., 2008	European/West Asians	America	NO	721/554	8
Neumann et al., 2009	European/West Asians	British	YES	790/257	9
Kalinderi et al., 2009	European/West Asians	Greece	YES	172/132	9
Moraitou et al., 2011	European/West Asians	Greece	NO	205/206	7
Setó-Salvia et al., 2012	European/West Asians	Spain	YES	225/186	9
Lesage et al., 2011a	European/West Asians	France	YES	1391/391	9
Emelyanov et al., 2012	European/West Asians	Russia	NO	330/240	8
Kumar et al., 2013	European/West Asians	Serbia	NO	360/348	8
Nalls et al., 2013	European/West Asians	European	NO	151/1962	7
Duran et al., 2013	European/West Asians	UK	YES	185/283	9
Asselta et al., 2014	European/West Asians	Italy	NO	2350/1111	8
Ran et al., 2016	European/West Asians	Sweden	NO	1625/2025	8
Crosiers et al., 2016	European/West Asians	Flanders-Belgian	YES	266/536	9
Török et al., 2016	European/West Asians	Hungary	NO	124/122	7
Jesús et al., 2016	European/West Asians	Spain	YES	532/542	9
Spitz et al., 2008	Hispanics	Brazil	NO	65/267	7
Dos Santos et al., 2010	Hispanics	Brazil	NO	110/155	7
Guimarães Bde et al., 2012	Hispanics	Brazil	NO	237/186	7
González-Del Rincón Mde et al., 2013	Hispanics	Mexico	NO	128/252	7
Clark et al., 2005	Mixed	Canada	NO	88/122	8
Clark et al., 2007	Mixed	America	YES	100/94	9
Nichols et al., 2009	Mixed	North America	NO	450/359	8
Han et al., 2016	Mixed	Canada	YES	225/110	9
Barber et al., 2017	Mixed	America	NO	106/283	7
Nishioka et al., 2010	African	Tunisia	NO	428/372	8
Lesage et al., 2011b	African	North Africa	YES	194/177	9
Barkhuizen et al., 2017	African	South Africa	NO	105/40	7

*PD, Parkinson's disease; AJ: Ashkenazi Jewish; NOS, Newcastle-Ottawa Scale*.

**Figure 2:**
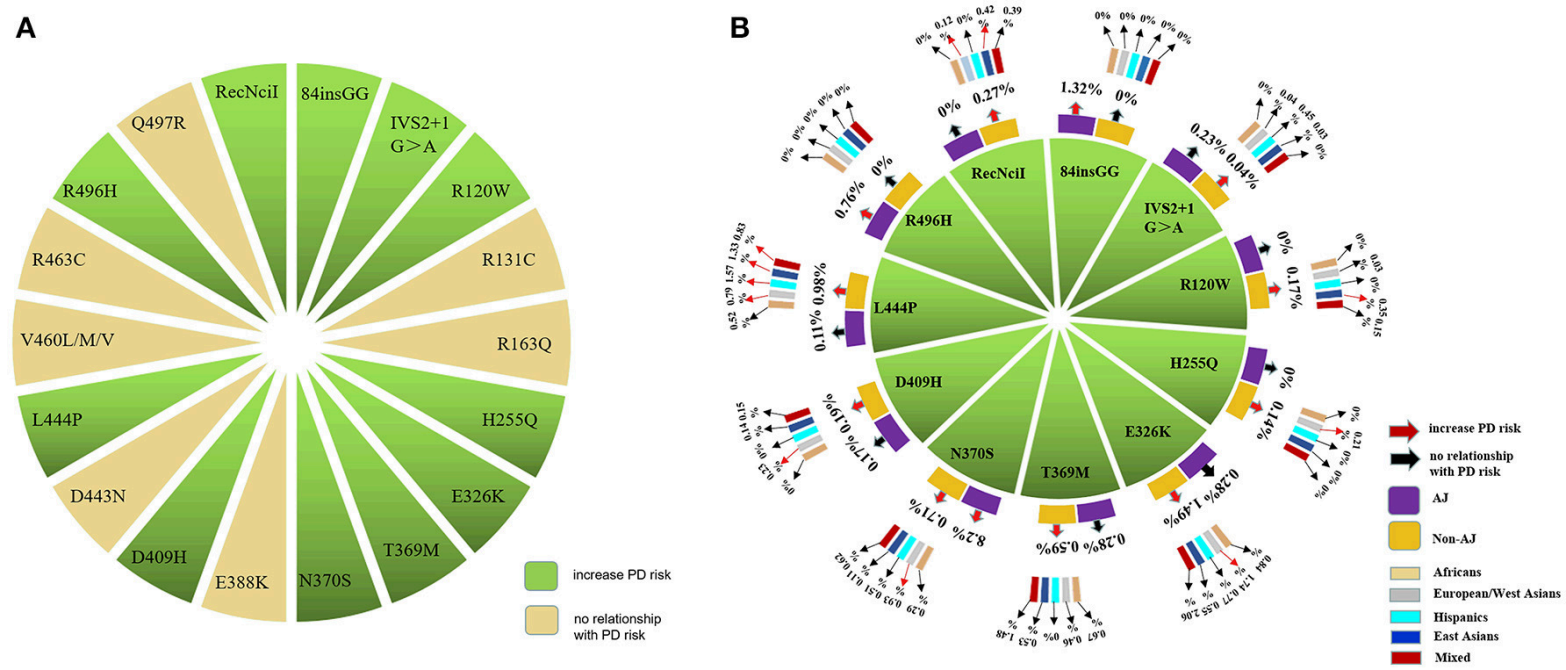
A total of 18 *GBA* variants were included and conducted in the meta-analysis. **(A)** 18 *GBA* variants related to PD were included for meta-analysis according to our inclusion and exclusion criteria. **(B)** 11 of 18 variants were found to increase the risk for PD. And the MAFs of the 11 risk variants were largely ethnicity dependent.

According to the detecting methods, 17 of 50 publications sequenced all exons of *GBA* in participants and reported a series of variants either associated with PD or not (Supplementary Table 1). And other 33 included studies just detected specific variants (Supplementary Table 2). Even though more than 130 variants of *GBA* were researched in PD cohorts of all included publications, most of which were only reported by one or two studies. We further conducted meta-analysis for 18 variants of all included variants as they had sufficient data (effected variant carriers reported in at least four articles) (Figure 2 and Supplementary Table 3).

We found that 11 of the 18 variants, including 84insGG, IVS2+1G>A, R120W, H255Q, E326K, T369M, N370S, D409H, L444P, R496H and RecNciI, increased the risk of PD in total populations (*p*: <0.00001, 0.00005, 0.0008, 0.005, <0.00001, 0.004, <0.00001, 0.0003, <0.00001, <0.0001, 0.0001) with the ORs ranging from 1.78 to 10.49 (Table 2 and Supplementary Figure 1). In subgroup analysis by ethnicities, in AJ populations, three variants of *GBA* were found to be in statistical difference between PD patients and controls. The variants listed by ORs from the top to the bottom were 84insGG, R496H, N370S (OR: 9.26–3.51; *p*: <0.00001, <0.0001, <0.00001). In total non-AJ populations, nine variants can increase the risk of PD. Results of variants from the highest OR values to the lowest OR values were L444P, R120W, IVS2+1G>A, H255Q, N370S, D409H, RecNciI, E326K, T369M (OR: 8.66–1.89; *p*: <0.00001, 0.0008, 0.02, 0.005, <0.00001, 0.001, 0.0001, <0.00001, 0.002). Among the non-AJ populations, pooled analysis from five different groups were done separately. In European/West Asians, six variants were associated with increased risk of PD. Variants having highest OR values to the lowest were L444P, N370S, H255Q, D409H, RecNciI, E326K (OR: 6.52–1.84; *p*: <0.00001, <0.00001, 0.005, 0.005, 0.04, <0.00001). L444P increased the risk of PD in Hispanics, East Asians and Mixed populations (OR: 15.44, 12.43, 7.33; *p*: 0.00004, <0.00001, 0.009). In East Asian populations, besides L444P, R120W and RecNciI can also increase the risk of PD (OR: 14.93, 3.56; *p*: 0.001, 0.003). For the lack of enough original studies, we were not able to conduct meta-analysis in Africa (Table 2 and Supplementary Figure 2).

**Table 2:** The association between variants included in *GBA* and the risk of PD.

Variants^*^	**Total population**	**AJ**	**Non-AJ**					
			**Total**	**Africans**	**European/West Asians**	**Hispanics**	**East Asians**	**Mixed**
84insGG	4 (984/6,166)	4 (984/6,166)	0 (0/0)	0 (0/0)	0 (0/0)	0 (0/0)	0 (0/0)	0 (0/0)
	**9.26 [4.02, 21.34]**	**9.26 [4.02, 21.34]**	NA	NA	NA	NA	NA	NA
	**<0.00001**	**<0.00001**	NA	NA	NA	NA	NA	NA
IVS2+1G>A	5 (1,346/6,933)	1 (420/4,138)	4 (926/2,795)	0 (0/0)	2 (336/2,245)	1 (110/155)	1 (480/395)	0 (0/0)
	**10.49 [2.81, 39.19]**	NA	**6.72 [1.42, 31.92]**	NA	NA	NA	NA	NA
	**0.00005**	NA	**0.02**	NA	NA	NA	NA	NA
R120W	5 (2,815/1,484)	0 (0/0)	5 (2,815/1,484)	0 (0/0)	1 (1,391/391)	0 (0/0)	3 (1,199/983)	1 (225/110)
	**8.61 [2.46, 30.14]**	NA	**8.61 [2.46, 30.14]**	NA	NA	NA	**14.93 [2.94, 75.78]**	NA
	**0.0008**	NA	**0.0008**	NA	NA	NA	**0.001**	NA
R131C	5 (3,094/1,652)	0 (0/0)	5 (3,094/1,652)	1 (194/177)	3 (2,366/931)	0 (0/0)	1 (534/544)	0 (0/0)
	2.42 [0.59, 9.84]	NA	2.42 [0.59, 9.84]	NA	1.66 [0.26, 10.50]	NA	NA	NA
	0.22	NA	0.22	NA	0.59	NA	NA	NA
R163Q	4 (1,179/1,065)	0 (0/0)	4 (1,179/1,065)	0 (0/0)	0 (0/0)	0 (0/0)	4 (1,179/1,065)	0 (0/0)
	0.90 [0.34, 2.38]	NA	0.90 [0.34, 2.38]	NA	NA	NA	0.90 [0.34, 2.38]	NA
	0.84	NA	0.84	NA	NA	NA	0.84	NA
H255Q	4 (922/969)	0 (0/0)	4 (922/969)	0 (0/0)	4 (922/969)	0 (0/0)	0 (0/0)	0 (0/0)
	**4.75 [1.60, 14.09]**	NA	**4.75 [1.60, 14.09]**	NA	**4.75 [1.60, 14.09]**	NA	NA	NA
	**0.005**	NA	**0.005**	NA	**0.005**	NA	NA	NA
E326K	14 (5,700/5,422)	1 (178/85)	13 (5,522/5,337)	1 (105/40)	7 (4,401/4,339)	1 (65/267)	2 (276/222)	2 (675/469)
	**1.97 [1.57, 2.46]**	NA	**1.98 [1.58, 2.49]**	NA	**1.84 [1.43, 2.37]**	NA	NA	NA
	**<0.00001**	NA	**<0.00001**	NA	**<0.00001**	NA	NA	NA
T369M	15 (4,738/3,879)	1 (178/85)	14 (4,560/3,794)	2 (299/217)	8 (3,394/2,922)	0 (0/0)	1 (92/92)	3 (775/563)
	**1.78 [1.20, 2.64]**	NA	**1.89 [1.27, 2.83]**	NA	1.29 [0.75, 2.23]	NA	NA	1.69 [0.77, 3.68]
	**0.004**	NA	**0.002**	NA	0.36	NA	NA	0.19
N370S	30 (13,431/16,522)	5 (1,144/6,258)	26 (12,287/10,264)	3 (727/589)	15 (9,916/8,066)	2 (347/341)	1 (328/300)	5 (969/968)
	**3.70 [3.04, 4.50]**	**3.51 [2.78, 4.43]**	**3.98 [2.82, 5.63]**	0.93 [0.24, 3.55]	**4.85 [3.21, 7.32]**	NA	NA	2.05 [0.76, 5.54]
	**<0.00001**	**<0.00001**	**<0.00001**	0.92	**<0.00001**	NA	NA	0.16
E388K	6 (4,667/4,525)	0 (0/0)	6 (4,667/4,525)	0 (0/0)	6 (4,667/4,525)	0 (0/0)	0 (0/0)	0 (0/0)
	1.11 [0.39, 3.13]	NA	1.11 [0.39, 3.13]	NA	1.11 [0.39, 3.13]	NA	NA	NA
	0.85	NA	0.85	NA	0.85	NA	NA	NA
D409H	14 (5,468/7,529)	1 (420/4,138)	13 (5,048/3,391)	0 (0/0)	8 (3,558/2,233)	0 (0/0)	4 (1,390/1,064)	1 (100/94)
	**3.84 [1.86, 7.91]**	NA	**3.39 [1.60, 7.16]**	NA	**3.77 [1.49, 9.53]**	NA	2.72 [0.68, 10.86]	NA
	**0.0003**	NA	**0.001**	NA	**0.005**	NA	0.16	NA
D443N	4 (3,485/3,507)	0 (0/0)	4 (3,485/3,507)	1 (194/177)	3 (3,291/3,330)	0 (0/0)	0 (0/0)	0 (0/0)
	0.92 [0.20, 4.32]	NA	0.92 [0.20, 4.32]	NA	1.47 [0.22, 9.94]	NA	NA	NA
	0.92	NA	0.92	NA	0.69	NA	NA	NA
L444P	43 (18,534/21,847)	1 (420/4,138)	42 (18,114/17,709)	1 (194/177)	18 (10,363/10,282)	4 (540/860)	15 (6,154/5,705)	4 (863/685)
	**8.60 [6.23, 11.87]**	NA	**8.66 [6.25, 12.01]**	NA	**6.52 [4.23, 10.05]**	**15.44 [3.44, 69.33]**	**12.43 [6.92, 22.31]**	**7.33 [1.66, 32.37]**
	**<0.00001**	NA	**<0.00001**	NA	**<0.00001**	**0.00004**	**<0.00001**	**0.009**
V460L/M/V	6 (2,567/1,428)	0 (0/0)	6 (2,567/1,428)	1 (194/177)	2 (1,563/523)	0 (0/0)	3 (810/728)	0 (0/0)
	0.62 [0.22, 1.80]	NA	0.62 [0.22, 1.80]	NA	NA	NA	0.63 [0.15, 2.77]	NA
	0.38	NA	0.38	NA	NA	NA	0.54	NA
R463C	5 (2,877/3,241)	0 (0/0)	5 (2,877/3,241)	0 (0/0)	5 (2,877/3,241)	0 (0/0)	0 (0/0)	0 (0/0)
	3.58 [0.88, 14.50]	NA	3.58 [0.88, 14.50]	NA	3.58 [0.88, 14.50]	NA	NA	NA
	0.07	NA	0.07	NA	0.07	NA	NA	NA
R496H	4 (984/6,166)	4 (984/6,166)	0 (0/0)	0 (0/0)	0 (0/0)	0 (0/0)	0 (0/0)	0 (0/0)
	**7.32 [2.82, 19.02]**	**7.32 [2.82, 19.02]**	NA	NA	NA	NA	NA	NA
	**<0.0001**	**<0.0001**	<0.0001	NA	NA	NA	NA	NA
Q497R	3 (460/314)	0 (0/0)	3 (460/314)	0 (0/0)	0 (0/0)	0 (0/0)	3 (460/314)	0 (0/0)
	1.41 [0.26, 7.57]	NA	1.41 [0.26, 7.57]	NA	NA	NA	1.41 [0.26, 7.57]	NA
	0.69	NA	0.69	NA	NA	NA	0.69	NA
RecNciI	17 (6,797/6,751)	0 (0/0)	17 (6,797/6,751)	1 (194/177)	7 (3,368/3,963)	1 (110/155)	5 (2,350/1,893)	3 (775/563)
	**3.21 [1.77, 5.79]**	NA	**3.21 [1.77, 5.79]**	NA	**3.24 [1.03, 10.20]**	NA	**3.56 [1.53, 8.29]**	1.91 [0.45, 8.09]
	**0.0001**	NA	**0.0001**	NA	**0.04**	NA	**0.003**	0.38

*PD, Parkinson's disease; AJ, Ashkenazi Jewish*.

* *Results for each variant was shown as Number of articles (No. patients/No. controls), OR [95% CI] and p value for all groups. OR, odds ratio. CI, confidence interval; NA, not available, limit to the number of publications, meta-analyses were not conducted in some subgroups. Bold OR and 95% CI meant statistically significance between a specific variant and risk of PD*.

Totally, the carriers' MAFs among all 11 risk variants of *GBA*, from the highest to the lowest, were E326K, N370S, L444P, T369M, RecNciI, 84insGG, D409H, R120W, H255Q, R496H, IVS2+1G>A with the range from to 1.46 to 0.06%. Besides, the frequencies were largely ethnicity dependent (Figure 2 and Supplementary Table 4). In AJ populations, the top five common variants by the MAFs were N370S, 84insGG, R496H, E326K, T369M (8.2–0.28%). In non-AJ populations as a whole, the variants with top five MAFs were E326K, L444P, N370S, T369M, RecNciI (1.49–0.27%). 84insGG and R496H were screened out only in AJ populations while RecNciI was exclusively existed in non-AJ populations. In subgroup analysis in non-AJ populations, the variants with high MAFs were similar with some specificities such as the L444P variant ranked the top MAFs in East Asian and Hispanics while E326K had the top MAFs in other three populations. Considering the limitations of the small sample size of case-control studies, we checked the allele frequencies of 16 variants among 18 (88insGG and RecNciI not covered) from the Genome Aggregation Database (gnomAD) (http://gnomad.broadinstitute.org/). The trends of the frequencies' differences between cases and controls reported in gnomAD for the 11 positive variants are comparable with our meta-analysis, and our results were even more significant (Supplementary Table 4).

During the sensitivity analysis, the pooled OR and 95% CI of all variants did not change significantly when deleted one article in turn every time. What's more, there were not significant publication biases from the symmetric shapes of all funnel plots (Supplementary Figures 3, 4).

## Discussion

We performed a comprehensive analysis between variants in *GBA* and the risk of PD, which was an update and a complement to previous meta-analysis (Chen et al., 2014; Zhao et al., 2016) on *GBA* gene. In order to reach more reliable conclusions, we incorporated as many relevant original studies as possible. Compared to the obvious heterogeneities between previous studies, what we provided here is a more precise assessment focused on the association between *GBA* variants and risk of PD in total populations with diverse ethnics.

In our analysis of total populations, we provided detailed information about the sequencing methods of each original article. By retrieving information from articles screening specific variants or the full exons of *GBA*, we included all the published positive results and negative results to reach a more precise conclusion. As can be seen from Supplementary Tables 1, 2, there were more than 130 variants of *GBA* in the association analysis in PD. They can either increase the risk of PD or have no relationship with the disease. In our meta-analysis, we only included 18 variants with enough original studies as supporting evidence. As a result, we found 11 variants associated with increased risk of PD. There were no quantitative results for other a hundred-odd variant on which further researches are needed to explain their role in PD. It is true that sequencing full exons of *GBA* could find new potential related variants in PD. These information support that sequencing the entire gene would be more informative than just genotyping the risk variants for PD.

**Figure 3:**
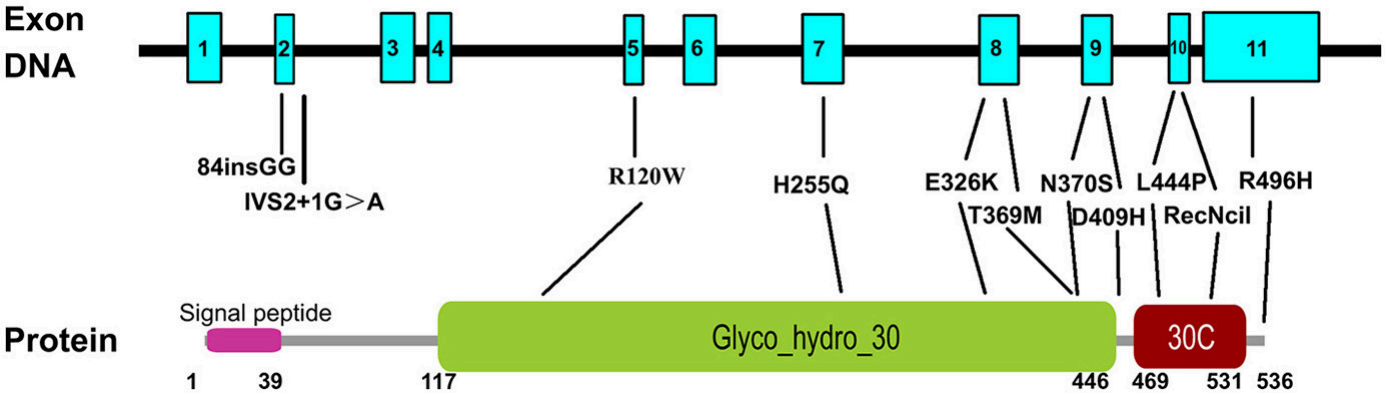
Schematic representation of the *GBA* gene and protein with the 11 risk variants. All variants are named following the common nomenclature which begins 39 codons downstream from the first ATG, excluding the 39-residue signal peptide. *GBA* has 11 exons and the protein has three domains, signal peptide, glyco_hydro_30, and glycol_hydro_30C. Numbers below the protein line indicate the boundaries of each domain.

To develop more specific genetic screening strategies, we did subgroup analysis by ethnicities in AJ populations and non-AJ populations based on different features of *GBA* mutations in these two ethnics. In AJ populations, the frequencies of *GBA* mutations ranged from 10 to 31% while in non-AJ populations, the frequencies fluctuated between 2.9 and 12% (Sun et al., 2010). Variants such as N370S in *GBA* was more common in AJ populations with European origin while L444P was more common in non-AJ populations such as China (Sidransky et al., 2009; Sun et al., 2010). Therefore, we conducted our meta-analysis in AJ populations and non-AJ populations separately. As a result, we successfully demonstrated significant ethnic heterogeneities in *GBA* variants included in our meta-analysis. 84insGG and R496H exclusively increased PD risks in AJ populations (MAF: 1.32%, 0.76%). As what were done by mutations including L444P, R120W, IVS2+1G>A, H255Q, D409H, RecNciI, E326K, T369M in non-AJ populations (MAF: 0.98, 0.17, 0.04, 0.14, 0.19, 0.27, 1.49, 0.59%). N370S was associated with the risk of PD in both ethnics (MAF: 8.20, 0.71%). In non-AJ populations, data from previous reports showed that the frequencies varied in different areas, such as 0.4% in North America, 1.7% in Norway etc. (Neumann et al., 2009). In that case, we did the meta-analysis on different districts in non-AJ populations in PD based on standard racial separations (Risch et al., 2002). From the results of five ethnics in non-AJ populations, N370S, H255Q, D409H, E326K were exclusively related to PD risks in European/West Asians (MAF: 0.93, 0.21, 0.23, 1.74%) while R120W in East Asians (MAF: 0.35%). L444P increased the risk of PD in all populations in non-AJ ethnicity [MAF: 1.33% (East Asians), 0.79% (European/West Asians), 1.57% (Hispanics), 0.52% (African), 0.83% (Mixed)]. In addition, combined with frequency analysis, we found risk variants of *GBA* in PD mainly located on exons 8, 9, and 10, such as E326K, T369M, N370S, D409H, and L444P. For future genetic analysis of *GBA* in PD, taking priority to screen the hot exons 8, 9, and 10 and focusing on those ethnicity dependent risk variants may be more time and money saving (Figures 2, 3). In that case, our meta-analysis can be a valuable reference for researchers to choose specific variants in building targeted reseq panel for PD patients according to different ethnics and groups. However, it's worth noticing that although those positive variants of *GBA* could increase the risk of PD, it does not necessarily mean that the carriers of *GBA* risk variants would develop PD. Because the etiology of PD is still unclear, genetic background is only one of the interactive factors contributing to the PD pathogenesis.

*GBA* gene encodes GCase. Heterozygous *GBA* mutations in PD were associated with decreased GCase activities (Ortega et al., 2016). And the activities of GCase in PD patients with *GBA* homozygotes were even lower than that of heterozygotes (Alcalay et al., 2015). Decreased GCase level could assist α-synuclein accumulation either by interfering protein clearance or by promoting protein aggregation in Lewy bodies. This mechanism were also identified in dementia with Lewy bodies (DLB), which added to the view that *GBA* is a main genetic risk factor for DLB (Beavan and Schapira, 2013). In addition to the aforementioned mechanism for enzymatic loss of function of GBA leading to PD, the gain of function mechanism was also discovered in *GBA* related PD where GCase co-localized with α-synuclein (Westbroek et al., 2011). As to the pathological changes underlying specific polymorphisms, although the information was rare, researchers observed reduced activity of GBA enzyme in brains of PD patients with heterozygous N370S mutation. And PD patients with heterozygous L444P mutation were also shown to have presynaptic dopaminergic neuronal dysfunction via neuroimaging examinations (Clark et al., 2007). Thus, whether it is the gain function or loss function of GBA enzyme leads to PD remains unclear.

In clinical use, distinct feature of *GBA* variants carriers in PD should be paid attention. In previous studies, our group observed unique clinical characteristics of *GBA*-related PD. Those patients carrying *GBA* variants were more likely to develop PD at earlier ages, develop bradykinesia as an initial symptom, have a family history and develop non-motor symptoms such as dementia (Zhang et al., 2015). In such case, *GBA* screening could be conducted in those special PD patients to increase the detection rate. What's more, patients could get more precise treatment in the future when their clinical features and genetic features were combined together.

However, the meta-analysis still has some inevitable limitations. First of all, some original studies without detailed information to calculate OR and 95% CI were not included. Second, the patients and controls of varies clinical features were not separated to do subgroup analysis which may cause biases. Third, due to the limitations of each original study such as the sequencing methods and the sample size, the heterogeneities cannot be totally avoided. For instance, when an original study screened only specific variants, the frequency of other variants may be underestimated.

## Conclusion

In conclusion, the contribution of *GBA* variants to the development of PD is racial dependent. 84insGG and R496H exclusively increased PD risks in AJ populations while L444P, R120W, IVS2+1G>A, H255Q, D409H, RecNciI, E326K, T369M in non-AJ populations. N370S increased the risk of PD in both ethnics. In non-AJ subgroup populations, N370S, H255Q, D409H, E326K exclusively increased PD risks in European/West Asians while R120W in East Asians. L444P increased the risk of PD in all groups in non-AJ ethnicity. Our results will do great help to future *GBA* screening in clinical use.

## Author contributions

YZ, LS, and BT: Conceived and designed the experiments; YZ, LS, and QS: Performed the experiments; YZ, LS, QS, BT: analyzed the data; YZ, LS, and BT: Wrote the manuscript; XZ, HP, and JG: Reference collection and data management.

### Conflict of interest statement

The authors declare that the research was conducted in the absence of any commercial or financial relationships that could be construed as a potential conflict of interest.
