# Supplementary material for: Integrated Genetic Analysis of Racial Differences of Common GBA Variants in Parkinson's Disease: A Meta-Analysis
Source: Front Mol Neurosci. 2018 Feb 15;11:43. doi: 10.3389/fnmol.2018.00043 (PMC5829555; doi:10.3389/fnmol.2018.00043)
Supplement: Supplementary file 1 [file DataSheet1.DOCX]

Supplementary Material

Integrated genetic analysis of racial differences of common GBA variants in parkinson's disease: a Meta-analysis

Yuan Zhang^1 †^, Li Shu^1 †^, Qiying Sun^2,3,4^, Xun Zhou^1^, Hongxu Pan^1^, Jifeng Guo^1, 3,4^, Beisha Tang^1, 3,4,5*^

**^†^** These authors have contributed equally to this work and are co-first authors.

^*^ Correspondence: Beisha Tang [bstang7398@163.com](mailto:bstang7398@163.com)

**Supplementary Table 1**: Variants reported in 17 studies full exons of *GBA* sequenced. Abbreviation: PD, Parkinson’s disease. CONT, controls. (n)*, No. of Ashkenazi Jewish in total Caucasian, other Caucasians are non-AJ.

| Year and First Author | Groups | Total NO. | Reported Variants |
| --- | --- | --- | --- |
| 2007Jose Bras | PD | 230 | K(-27)R,R2L,E326K,T369M,N370S,E388K,N396T,D409H,L444P |
|  | CONT | 430 |  |
| 2007L.N. Clark | PD | 278(178) * | 84insGG,P175P,E326K,T369M,N370S,D409H,R496H,L444P,RecNciI |
|  | CONT | 179(85) * |  |
| 2007Shira G. Ziegler | PD | 92 | L174P,E326K,T369M,D409H,L444P,V460M,Q497R |
|  | CONT | 92 |  |
| 2009Kallirhoe Kalinderi | PD | 172 | H255Q,L268L,S271G,E326K,R329H,D409H,L445P,V460L,T482K |
|  | CONT | 132 |  |
| 2009*Jun Mitsui* | PD | 534 | R120W,R131C,N188S,G193W,F213I,R329C,L444P,R496C,RecNcil, A456P-V460V,R120W-N188R-V191G-S196P-F213I |
|  | CONT | 544 |  |
| 2009Juliane Neumann | PD | 790 | K7E,R131C,G193E,R257Q,N370S,D380A,D409H,L444P,D443N, V458L,R463C,c.1263-1317 del55,RecA456P,RecNcil |
|  | CONT | 257 |  |
| 2011Suzanne Lesage(1) | PD | 1391 | K(-27)R,K79M,G80R,G113C,G113A,I119L,R120W,S125N,R131C,D140H, S173SfsX50,A190A,G202R,P246L,Y304C,Y313Y,T323I,E326K,R329C,S364N,T369M,N370S,G377S,E388K,D409H,L444P,P452L,R463C,R463H,A446A,RecNciI,RecA456P, Rec△5 |
|  | CONT | 391 |  |
| 2011S. Lesage(2) | PD | 194 | K(-27)R,R131C,E326K,T369M,N370S,D443N,L444P,RecNciI |
|  | CONT | 177 |  |
| 2011Nu´ ria Seto´ -Salvia, BS | PD | 225 | M123T,L144V,G202R,I260T,T369M,N370S,W393R,D409H,L444P, S488T,RecNciI |
|  | CONT | 186 |  |
| 2012Jung Mi Choi | PD | 277 | I(-20)V,R163Q,N188S,P201H,R257Q,L268L,S271G,R277C,F347L, N370S,L444P,K466K |
|  | CONT | 100 |  |
| 2013Raquel Duran | PD | 185 | E326K,N370S,L444P,RecNcil,R463C |
|  | CONT | 283 |  |
| 2014Teeratorn Pulkes | PD | 480 | IVS2+1G＞A,L444P,N386K,P428S,V398fsX404 |
|  | CONT | 395 |  |
| 2014Yuanzhe Li | PD | 147 | I(-20)V, G64V, R120W,W393X,D409H,L444P,I489V，RecNciI |
|  | CONT | 100 |  |
| 2014Zhe Yu | PD | 184 | R163Q, F213I, L264I,L314V , E326K, F347L, S364S, V375L, L444P, A456P, Q497RRecNciI , 5-bp deletion (c.334_338delCAGAA) |
|  | CONT | 130 |  |
| 2015Fabin Han | PD | 225 | 5'UTR-A/G,S(-35)N,S13L,R120W,E326K,T369M,N370S,L444P, RecNcil(L444P-A456P-V460V),RecTL(del55+D409H+RecNcil) |
|  | CONT | 110 |  |
| 2016David Crosiers | PD | 266 | G(-1)R,D140H,Q256SfsX9,L324P,E326K,T369M,N379S,L444P, H490R,RecNcil |
|  | CONT | 536 |  |
| 2016Silvia Jesu´s | PD | 532 | 116-8C＞T,W312R,E326K,T369M,N370S,L444P,V457D, |
|  | CONT | 542 |  |
