# Supplementary material for: Integrated Genetic Analysis of Racial Differences of Common GBA Variants in Parkinson's Disease: A Meta-Analysis
Source: Front Mol Neurosci. 2018 Feb 15;11:43. doi: 10.3389/fnmol.2018.00043 (PMC5829555; doi:10.3389/fnmol.2018.00043)
Supplement: Supplementary file 2 [file DataSheet2.DOCX]

Supplementary Material

Integrated genetic analysis of racial differences of common GBA variants in parkinson's disease: a Meta-analysis

Yuan Zhang^1 †^, Li Shu^1 †^, Qiying Sun^2,3,4^, Xun Zhou^1^, Hongxu Pan^1^, Jifeng Guo^1, 3,4^, Beisha Tang^1, 3,4,5*^

**^†^** These authors have contributed equally to this work and are co-first authors.

^*^ Correspondence: Beisha Tang [bstang7398@163.com](mailto:bstang7398@163.com)

**Supplementary Table 2**: Specific variants of *GBA* researched in 33 studies. Abbreviation: PD, Parkinson’s disease. CONT, controls. (n)*, No. of Ashkenazi Jewish in total Caucasian. Bold variants indicated none of the participants carried the variants in the studies, which only included in frequency analysis instead of pooled risk analysis.

| Year and First Author | Groups | Total NO. | Researched Variants(mutation and polymorphism) |
| --- | --- | --- | --- |
| 2004Judith Aharon-Peretz | PD | 99(99)* | 84GG,**IVS2+1G＞A**, N370S,**V394L，L444P,**R496H |
|  | CONT | 1543(1543) * |  |
| 2005Lorraine N. Clark | PD | 160(160) * | N370S |
|  | CONT | 92(92) * |  |
| 2005Christine Sato, BSc | PD | 88 | **84insGG,IVS2+1G＞A, K198T,R329C**,N370S,L444P, RecNciI |
|  | CONT | 122 |  |
| 2006M. Toft | PD | 311 | N370S,L444P |
|  | CONT | 474 |  |
| 2007Elvira V. De Marco | PD | 395 | N370S,L444P |
|  | CONT | 483 |  |
| 2007Mariana Spitz | PD | 65 | E326K,**N370S,G377S**,L444P,**V460V** |
|  | CONT | 267 |  |
| 2007*Eng-King Tan* | PD | 331 | N370S,L444P |
|  | CONT | 347 |  |
| 2007Yih-Ru Wu | PD | 518 | R120W,L444P,,RecNcil |
|  | CONT | 339 |  |
| 2008*Ignacio F. Mata* | PD | 721 | N370S,L444P |
|  | CONT | 554 |  |
| 2008Z. Gan-Or | PD | 420(420) * | 84GG, IVS2+1,N370S,V394L,D409H,L444P,R496H,RecTL |
|  | CONT | 4138(4138) * |  |
| 2008*Usha Gutt* | PD | 184 | R131S, R163Q, L174P,S271G,**N370S**,D409H,L444P,Q497R |
|  | CONT | 92 |  |
| 2008W.C. Nichols | PD | 450 | E326K,T369M,N370S,L444P,RecNciI |
|  | CONT | 359 |  |
| 2010Adriana Vaz dos Santos | PD | 110 | **84GG**，IVS2+1G＞A,**N370S**,G377S,L444P,RecNciI |
|  | CONT | 155 |  |
| 2010F.-Y. Hu | PD | 328 | N370S |
|  | CONT | 300 |  |
| 2010Kenya Nishioka | PD | 428 | N370S,K186R,K-26R |
|  | CONT | 372 |  |
| 2010X.-Y. Mao | PD | 616 | L444P |
|  | CONT | 411 |  |
| 2010Qi-Ying Sun | PD | 402 | **F213I,R353W,N370S**,L444P |
|  | CONT | 413 |  |
| 2011C.-L. Huang | PD | 967 | **R120W,L174P,**D409H,L444P,**Q497R**,RecNciI |
|  | CONT | 780 |  |
| 2011Marina Moraitou | PD | 205 | Y108C,**R120W**,H255Q,T369M,D409H,L444P,**Q497R**, IVS10-1G＞A,**IVS6-2A＞G** |
|  | CONT | 206 |  |
| 2012Anton Emelyanov | PD | 330 | N370S,L444P |
|  | CONT | 240 |  |
| 2012Beatriz de Carvalho Guimarães | PD | 237 | N370S,L444P |
|  | CONT | 186 |  |
| 2012K. R. Kumar | PD | 360 | H255Q,T369M,N370S,D380V,E388K,D409H,L444P,A456P,V459V, R463C,RecNcil |
|  | CONT | 348 |  |
| 2012Youpei Wang | PD | 208 | **R120W,N370S**,L444P |
|  | CONT | 298 |  |
| 2012Xiong Zhang | PD | 195 | **R120W,N370S**,L444P |
|  | CONT | 443 |  |
| 2013*M de L Gonz´ alez-del Rinc´ on* | PD | 128 | **N370S**, L444P |
|  | CONT | 252 |  |
| 2013*Michael A. Nalls* | PD | 151 | L105R,D140H,Q256S,L324P,**N370S**,E388K,D443N,L444P, R463C, ReciNciI, IVS2+1G＞A, |
|  | CONT | 1962 |  |
| 2014Rosanna Asselta | PD | 2350 | N370S,E388K,D443N,L444P,IVS8-24T＞G,IVS9-36C＞G,  IVS9-5T＞A,IVS9+32C＞T,IVS10+1G＞T,IVS10+8C＞A |
|  | CONT | 1111 |  |
| 2015Guo ji-feng | PD | 1061 | L444P |
|  | CONT | 1066 |  |
| 2015E. Dagan | PD | 287(287) * | 84GG,**IVS2+1G＞A**,N370S,V394L,**L444P**,R496H |
|  | CONT | 400(400) * |  |
| 2016Rita Török | PD | 124 | **R120W, N370S**,L444P |
|  | CONT | 122 |  |
| 2016Caroline Ran | PD | 1625 | E326K, N370S,L444P |
|  | CONT | 2025 |  |
| 2017Melinda Barkhuizen | PD | 105 | G35A,E326K,I368T,T369M,N370S,P387L,K441N |
|  | CONT | 40 |  |
| 2017Thomas R. Barber | PD | 106 | N370S,**L444P** |
|  | CONT | 283 |  |
