# Supplementary material for: Integrated Genetic Analysis of Racial Differences of Common GBA Variants in Parkinson's Disease: A Meta-Analysis
Source: Front Mol Neurosci. 2018 Feb 15;11:43. doi: 10.3389/fnmol.2018.00043 (PMC5829555; doi:10.3389/fnmol.2018.00043)
Supplement: Supplementary file 3 [file DataSheet3.DOCX]

Supplementary Material

Integrated genetic analysis of racial differences of common GBA variants in parkinson's disease: a Meta-analysis

Yuan Zhang^1 †^, Li Shu^1 †^, Qiying Sun^2,3,4^, Xun Zhou^1^, Hongxu Pan^1^, Jifeng Guo^1, 3,4^, Beisha Tang^1, 3,4,5*^

**^†^** These authors have contributed equally to this work and are co-first authors.

^*^ Correspondence: Beisha Tang [bstang7398@163.com](mailto:bstang7398@163.com)

**Supplementary Table 3**: Genotypes’ attributions of 18 *GBA* variants in all publications included. Abbreviation: PD, Parkinson’s disease. AA, wild type. Aa, heterozygous carriers. Aa, homozygous carriers. (n)*, No. of Ashkenazi Jewish. #, RecNciI is recombinant allele which used AA described the wild type and Aa for the recombinant one (L444P-A456P-V460V). NA, not available.

| Year and Author | Variants | Genotype NO. of PD patients | | | Genotype NO. of Controls | | |
| --- | --- | --- | --- | --- | --- | --- | --- |
|  |  | AA | Aa | aa | AA | Aa | aa |
| 2004Judith Aharon-Peretz | 84insGG | 95 | 4 | 0 | 1540 | 3 | 0 |
| 2007Jose Bras | 84insGG | 230 | 0 | 0 | 430 | 0 | 0 |
| 2007L.N. Clark | 84insGG | 273(173) * | 5(5) * | 0 | 179(85) * | 0 | 0 |
| 2007Shira G. Ziegler | 84insGG | 92 | 0 | 0 | 92 | 0 | 0 |
| 2008Z. Gan-Or | 84insGG | 412 | 8 | 0 | 4131 | 7 | 0 |
| 2009Juliane Neumann | 84insGG | 790 | 0 | 0 | 257 | 0 | 0 |
| 2009Jun Mitsui | 84insGG | 534 | 0 | 0 | 544 | 0 | 0 |
| 2009Kallirhoe Kalinderi | 84insGG | 172 | 0 | 0 | 132 | 0 | 0 |
| 2010Adriana Vaz dos Santos | 84insGG | 110 | 0 | 0 | 155 | 0 | 0 |
| 2011Nu´ ria Seto´ -Salvia, BS | 84insGG | 225 | 0 | 0 | 186 | 0 | 0 |
| 2011S. Lesage(2) | 84insGG | 194 | 0 | 0 | 177 | 0 | 0 |
| 2011Suzanne Lesage(1) | 84insGG | 1291 | 0 | 0 | 391 | 0 | 0 |
| 2012Jung Mi Choi | 84insGG | 277 | 0 | 0 | 100 | 0 | 0 |
| 2013Raquel Duran | 84insGG | 185 | 0 | 0 | 283 | 0 | 0 |
| 2014Teeratorn Pulkes | 84insGG | 480 | 0 | 0 | 395 | 0 | 0 |
| 2014Yuanzhe Li | 84insGG | 147 | 0 | 0 | 100 | 0 | 0 |
| 2014Zhe Yu | 84insGG | 184 | 0 | 0 | 130 | 0 | 0 |
| 2015E. Dagan | 84insGG | 278(278) * | 9(9)* | 0 | 398(398) * | 2(2)* | 0 |
| 2015Fabin Han | 84insGG | 225 | 0 | 0 | 110 | 0 | 0 |
| 2016David Crosiers | 84insGG | 266 | 0 | 0 | 536 | 0 | 0 |
| 2016Silvia Jesu´s | 84insGG | 532 | 0 | 0 | 542 | 0 | 0 |
| 2007Jose Bras | IVS2+1G＞A | 230 | 0 | 0 | 430 | 0 | 0 |
| 2007L.N. Clark | IVS2+1G＞A | 278(178) * | 0 | 0 | 179(85) * | 0 | 0 |
| 2007Shira G. Ziegler | IVS2+1G＞A | 92 | 0 | 0 | 92 | 0 | 0 |
| 2008Z. Gan-Or | IVS2+1G＞A | 416 | 4 | 0 | 4137 | 1 | 0 |
| 2009Juliane Neumann | IVS2+1G＞A | 790 | 0 | 0 | 257 | 0 | 0 |
| 2009Jun Mitsui | IVS2+1G＞A | 534 | 0 | 0 | 544 | 0 | 0 |
| 2009Kallirhoe Kalinderi | IVS2+1G＞A | 172 | 0 | 0 | 132 | 0 | 0 |
| 2010Adriana Vaz dos Santos | IVS2+1G＞A | 109 | 1 | 0 | 155 | 0 | 0 |
| 2011Nu´ ria Seto´ -Salvia, BS | IVS2+1G＞A | 225 | 0 | 0 | 186 | 0 | 0 |
| 2011S. Lesage(2) | IVS2+1G＞A | 194 | 0 | 0 | 177 | 0 | 0 |
| 2011Suzanne Lesage(1) | IVS2+1G＞A | 1291 | 0 | 0 | 391 | 0 | 0 |
| 2012Jung Mi Choi | IVS2+1G＞A | 277 | 0 | 0 | 100 | 0 | 0 |
| 2013Michael A. Nalls | IVS2+1G＞A | 149 | 2 | 0 | 1962 | 0 | 0 |
| 2013Raquel Duran | IVS2+1G＞A | 184 | 1 | 0 | 283 | 0 | 0 |
| 2014Teeratorn Pulkes | IVS2+1G＞A | 479 | 1 | 0 | 395 | 0 | 0 |
| 2014Yuanzhe Li | IVS2+1G＞A | 147 | 0 | 0 | 100 | 0 | 0 |
| 2014Zhe Yu | IVS2+1G＞A | 184 | 0 | 0 | 130 | 0 | 0 |
| 2015. Dagan | IVS2+1G＞A | 287(287) * | 0 | 0 | 400(400) * | 0 | 0 |
| 2015Fabin Han | IVS2+1G＞A | 225 | 0 | 0 | 110 | 0 | 0 |
| 2016David Crosiers | IVS2+1G＞A | 266 | 0 | 0 | 536 | 0 | 0 |
| 2016Silvia Jesu´s | IVS2+1G＞A | 532 | 0 | 0 | 542 | 0 | 0 |
| 2007Jose Bras | R120W | 230 | 0 | 0 | 430 | 0 | 0 |
| 2007Yih-Ru Wu | R120W | 517 | 1 | 0 | 339 | 0 | 0 |
| 2007L.N. Clark | R120W | 278(178) * | 0 | 0 | 179(85) * | 0 | 0 |
| 2007Shira G. Ziegler | R120W | 92 | 0 | 0 | 92 | 0 | 0 |
| 2009Juliane Neumann | R120W | 790 | 0 | 0 | 257 | 0 | 0 |
| 2009Jun Mitsui | R120W | 519 | 15 | 0 | 544 | 0 | 0 |
| 2009Kallirhoe Kalinderi | R120W | 172 | 0 | 0 | 132 | 0 | 0 |
| 2011C.-L. Huang | R120W | 967 | 0 | 0 | 780 | 0 | 0 |
| 2011Marina Moraitou | R120W | 205 | 0 | 0 | 206 | 0 | 0 |
| 2011Nu´ ria Seto´ -Salvia, BS | R120W | 225 | 0 | 0 | 186 | 0 | 0 |
| 2011S. Lesage(2) | R120W | 194 | 0 | 0 | 177 | 0 | 0 |
| 2011Suzanne Lesage(1) | R120W | 1289 | 2 | 0 | 391 | 0 | 0 |
| 2012Jung Mi Choi | R120W | 277 | 0 | 0 | 100 | 0 | 0 |
| 2012Xiong Zhang | R120W | 195 | 0 | 0 | 443 | 0 | 0 |
| 2012Youpei Wang | R120W | 208 | 0 | 0 | 298 | 0 | 0 |
| 2013Raquel Duran | R120W | 185 | 0 | 0 | 283 | 0 | 0 |
| 2014Teeratorn Pulkes | R120W | 480 | 0 | 0 | 395 | 0 | 0 |
| 2014Yuanzhe Li | R120W | 138 | 9 | 0 | 100 | 0 | 0 |
| 2014Zhe Yu | R120W | 184 | 0 | 0 | 130 | 0 | 0 |
| 2015Fabin Han | R120W | 224 | 1 | 0 | 110 | 0 | 0 |
| 2016David Crosiers | R120W | 266 | 0 | 0 | 536 | 0 | 0 |
| 2016Rita Török | R120W | 124 | 0 | 0 | 122 | 0 | 0 |
| 2016Silvia Jesu´s | R120W | 532 | 0 | 0 | 542 | 0 | 0 |
| 2007Jose Bras | R131C | 230 | 0 | 0 | 430 | 0 | 0 |
| 2007L.N. Clark | R131C | 278(178) * | 0 | 0 | 179(85) * | 0 | 0 |
| 2007Shira G. Ziegler | R131C | 92 | 0 | 0 | 92 | 0 | 0 |
| 2009Juliane Neumann | R131C | 789 | 1 | 0 | 257 | 0 | 0 |
| 2009Jun Mitsui | R131C | 533 | 1 | 0 | 544 | 0 | 0 |
| 2009Kallirhoe Kalinderi | R131C | 172 | 0 | 0 | 132 | 0 | 0 |
| 2011Nu´ ria Seto´ -Salvia, BS | R131C | 225 | 0 | 0 | 186 | 0 | 0 |
| 2011S. Lesage(2) | R131C | 192 | 1 | 1 | 177 | 0 | 0 |
| 2011Suzanne Lesage(1) | R131C | 1390 | 1 | 0 | 391 | 0 | 0 |
| 2012Jung Mi Choi | R131C | 277 | 0 | 0 | 100 | 0 | 0 |
| 2013Raquel Duran | R131C | 184 | 1 | 0 | 283 | 0 | 0 |
| 2014Teeratorn Pulkes | R131C | 480 | 0 | 0 | 395 | 0 | 0 |
| 2014Yuanzhe Li | R131C | 147 | 0 | 0 | 100 | 0 | 0 |
| 2014Zhe Yu | R131C | 184 | 0 | 0 | 130 | 0 | 0 |
| 2015Fabin Han | R131C | 225 | 0 | 0 | 110 | 0 | 0 |
| 2016David Crosiers | R131C | 266 | 0 | 0 | 536 | 0 | 0 |
| 2016Silvia Jesu´s | R131C | 532 | 0 | 0 | 542 | 0 | 0 |
| 2007Jose Bras | R163Q | 230 | 0 | 0 | 430 | 0 | 0 |
| 2007L.N. Clark | R163Q | 278(178) * | 0 | 0 | 179(85) * | 0 | 0 |
| 2007Shira G. Ziegler | R163Q | 92 | 0 | 0 | 92 | 0 | 0 |
| 2008Usha Gutt | R163Q | 183 | 1 | 0 | 92 | 0 | 0 |
| 2009Juliane Neumann | R163Q | 790 | 0 | 0 | 257 | 0 | 0 |
| 2009Jun Mitsui | R163Q | 530 | 4 | 0 | 537 | 7 | 0 |
| 2009Kallirhoe Kalinderi | R163Q | 172 | 0 | 0 | 132 | 0 | 0 |
| 2011Nu´ ria Seto´ -Salvia, BS | R163Q | 225 | 0 | 0 | 186 | 0 | 0 |
| 2011S. Lesage(2) | R163Q | 194 | 0 | 0 | 177 | 0 | 0 |
| 2011Suzanne Lesage(1) | R163Q | 1291 | 0 | 0 | 391 | 0 | 0 |
| 2012Jung Mi Choi | R163Q | 276 | 1 | 0 | 291 | 0 | 0 |
| 2013Raquel Duran | R163Q | 185 | 0 | 0 | 283 | 0 | 0 |
| 2014Teeratorn Pulkes | R163Q | 480 | 0 | 0 | 395 | 0 | 0 |
| 2014Yuanzhe Li | R163Q | 147 | 0 | 0 | 100 | 0 | 0 |
| 2014Zhe Yu | R163Q | 183 | 1 | 0 | 130 | 0 | 0 |
| 2015Fabin Han | R163Q | 225 | 0 | 0 | 110 | 0 | 0 |
| 2016David Crosiers | R163Q | 266 | 0 | 0 | 536 | 0 | 0 |
| 2016Silvia Jesu´s | R163Q | 532 | 0 | 0 | 542 | 0 | 0 |
| 2007Jose Bras | H255Q | 230 | 0 | 0 | 430 | 0 | 0 |
| 2007L.N. Clark | H255Q | 278(178) * | 0 | 0 | 179(85) * | 0 | 0 |
| 2007Shira G. Ziegler | H255Q | 92 | 0 | 0 | 92 | 0 | 0 |
| 2009Juliane Neumann | H255Q | 790 | 0 | 0 | 257 | 0 | 0 |
| 2009Jun Mitsui | H255Q | 534 | 0 | 0 | 544 | 0 | 0 |
| 2009Kallirhoe Kalinderi | H255Q | 168 | 4 | 0 | 132 | 0 | 0 |
| 2011Marina Moraitou | H255Q | 198 | 7 | 0 | 205 | 1 | 0 |
| 2011Nu´ ria Seto´ -Salvia, BS | H255Q | 225 | 0 | 0 | 186 | 0 | 0 |
| 2011S. Lesage(2) | H255Q | 194 | 0 | 0 | 177 | 0 | 0 |
| 2011Suzanne Lesage(1) | H255Q | 1291 | 0 | 0 | 391 | 0 | 0 |
| 2012Jung Mi Choi | H255Q | 277 | 0 | 0 | 100 | 0 | 0 |
| 2012K. R. Kumar | H255Q | 354 | 6 | 0 | 346 | 2 | 0 |
| 2013Raquel Duran | H255Q | 184 | 1 | 0 | 283 | 0 | 0 |
| 2014Teeratorn Pulkes | H255Q | 480 | 0 | 0 | 395 | 0 | 0 |
| 2014Yuanzhe Li | H255Q | 147 | 0 | 0 | 100 | 0 | 0 |
| 2014Zhe Yu | H255Q | 184 | 0 | 0 | 130 | 0 | 0 |
| 2015Fabin Han | H255Q | 225 | 0 | 0 | 110 | 0 | 0 |
| 2016David Crosiers | H255Q | 266 | 0 | 0 | 536 | 0 | 0 |
| 2016Silvia Jesu´s | H255Q | 532 | 0 | 0 | 542 | 0 | 0 |
| 2007Jose Bras | E326K | 228 | 2 | 0 | 427 | 3 | 0 |
| 2007L.N. Clark | E326K | 277(177) * | 1(1) * | 0 | 178(84) * | 1(1) * | 0 |
| 2007Mariana Spitz | E326K | 64 | 1 | 0 | 267 | 0 | 0 |
| 2007Shira G. Ziegler | E326K | 74 | 18 | 0 | 92 | 0 | 0 |
| 2008W.C. Nichols | E326K | 422 | 28 | 0 | 348 | 11 | 0 |
| 2009Juliane Neumann | E326K | 790 | 0 | 0 | 257 | 0 | 0 |
| 2009Jun Mitsui | E326K | 534 | 0 | 0 | 544 | 0 | 0 |
| 2009Kallirhoe Kalinderi | E326K | 171 | 1 | 0 | 131 | 1 | 0 |
| 2011Nu´ ria Seto´ -Salvia, BS | E326K | 225 | 0 | 0 | 186 | 0 | 0 |
| 2011S. Lesage(2) | E326K | 194 | 0 | 0 | 177 | 0 | 0 |
| 2011Suzanne Lesage(1) | E326K | 1342 | 43 | 6 | 383 | 8 | 0 |
| 2012Jung Mi Choi | E326K | 277 | 0 | 0 | 100 | 0 | 0 |
| 2013Raquel Duran | E326K | 171 | 12 | 2 | 283 | 0 | 0 |
| 2013Raquel Duran | E326K | 185 | 0 | 0 | 283 | 0 | 0 |
| 2014Teeratorn Pulkes | E326K | 480 | 0 | 0 | 395 | 0 | 0 |
| 2014Yuanzhe Li | E326K | 147 | 0 | 0 | 100 | 0 | 0 |
| 2014Zhe Yu | E326K | 183 | 1 | 0 | 130 | 0 | 0 |
| 2015Fabin Han | E326K | 221 | 4 | 0 | 106 | 4 | 0 |
| 2016Caroline Ran | E326K | 1450 | 90 | 0 | 1872 | 65 | 0 |
| 2016David Crosiers | E326K | 254 | 12 | 0 | 521 | 15 | 0 |
| 2016Silvia Jesu´s | E326K | 516 | 16 | 0 | 529 | 13 | 0 |
| 2017Melinda Barkhuizen | E326K | 100 | 5 | 0 | 39 | 1 | 0 |
| 2007Jose Bras | T369M | 228 | 2 | 0 | 425 | 5 | 0 |
| 2007L.N. Clark | T369M | 275(177)* | 3(1) * | 0 | 176(87) * | 3(2) * | 0 |
| 2007Shira G. Ziegler | T369M | 74 | 18 | 0 | 92 | 0 | 0 |
| 2008W.C. Nichols | T369M | 440 | 10 | 0 | 355 | 4 | 0 |
| 2009Juliane Neumann | T369M | 790 | 0 | 0 | 257 | 0 | 0 |
| 2009Jun Mitsui | T369M | 534 | 0 | 0 | 544 | 0 | 0 |
| 2009Kallirhoe Kalinderi | T369M | 172 | 0 | 0 | 132 | 0 | 0 |
| 2011Marina Moraitou | T369M | 205 | 0 | 0 | 205 | 1 | 0 |
| 2011Nu´ ria Seto´ -Salvia, BS | T369M | 223 | 2 | 0 | 186 | 0 | 0 |
| 2011S. Lesage(2) | T369M | 192 | 2 | 0 | 176 | 1 | 0 |
| 2011Suzanne Lesage(1) | T369M | 1374 | 14 | 3 | 390 | 1 | 0 |
| 2012Jung Mi Choi | T369M | 277 | 0 | 0 | 100 | 0 | 0 |
| 2012K. R. Kumar | T369M | 353 | 7 | 0 | 342 | 6 | 0 |
| 2013Raquel Duran | T369M | 184 | 1 | 0 | 282 | 1 | 0 |
| 2014Teeratorn Pulkes | T369M | 480 | 0 | 0 | 395 | 0 | 0 |
| 2014Yuanzhe Li | T369M | 147 | 0 | 0 | 100 | 0 | 0 |
| 2014Zhe Yu | T369M | 184 | 0 | 0 | 130 | 0 | 0 |
| 2015Fabin Han | T369M | 214 | 11 | 0 | 106 | 4 | 0 |
| 2016David Crosiers | T369M | 263 | 3 | 0 | 525 | 11 | 0 |
| 2016Silvia Jesu´s | T369M | 527 | 5 | 0 | 540 | 2 | 0 |
| 2016Silvia Jesu´s | T369M | 532 | 0 | 0 | 542 | 0 | 0 |
| 2017Melinda Barkhuizen | T369M | 103 | 2 | 0 | 39 | 1 | 0 |
| 2004Judith Aharon-Peretz | N370S | 73 | 23 | 3 | 1451 | 92 | 0 |
| 2005Christine Sato | N370S | 87 | 1 | 0 | 121 | 1 | 0 |
| 2005Lorraine N. Clark | N370S | 143 | 15 | 2 | 88 | 4 | 0 |
| 2007Jose Bras | N370S | 225 | 5 | 0 | 427 | 3 | 0 |
| 2007L.N. Clark | N370S | 255(178)* | 21(19) * | 2 | 176(86)* | 3(3) * | 0 |
| 2008W.C. Nichols | N370S | 444 | 6 | 0 | 356 | 3 | 0 |
| 2008Z. Gan-Or | N370S | 368 | 52 | 2 | 3901 | 235 | 2 |
| 2009Juliane Neumann | N370S | 782 | 8 | 0 | 256 | 1 | 0 |
| 2009Jun Mitsui | N370S | 534 | 0 | 0 | 544 | 0 | 0 |
| 2009Kallirhoe Kalinderi | N370S | 172 | 0 | 0 | 132 | 0 | 0 |
| 2010Adriana Vaz dos Santos | N370S | 108 | 1 | 1 | 155 | 0 | 0 |
| 2010Adriana Vaz dos Santos | N370S | 110 | 0 | 0 | 155 | 0 | 0 |
| 2010F.-Y. Hu | N370S | 322 | 6 | 0 | 298 | 2 | 0 |
| 2010Kenya Nishioka | N370S | 394 | 1 | 0 | 369 | 3 | 0 |
| 2010Qi-Ying Sun | N370S | 402 | 0 | 0 | 413 | 0 | 0 |
| 2011Marina Moraitou | N370S | 199 | 6 | 0 | 202 | 4 | 0 |
| 2011Nu´ ria Seto´ -Salvia, BS | N370S | 220 | 4 | 1 | 186 | 0 | 0 |
| 2011S. Lesage(2) | N370S | 192 | 2 | 0 | 177 | 0 | 0 |
| 2011Suzanne Lesage(1) | N370S | 1346 | 42 | 3 | 389 | 2 | 0 |
| 2012Anton Emelyanov | N370S | 327 | 2 | 0 | 240 | 0 | 0 |
| 2012Beatriz de Carvalho Guimarães | N370S | 234 | 3 | 0 | 186 | 0 | 0 |
| 2012Jung Mi Choi | N370S | 277 | 0 | 0 | 291 | 0 | 0 |
| 2012K. R. Kumar | N370S | 351 | 8 | 1 | 348 | 0 | 0 |
| 2012Xiong Zhang | N370S | 195 | 0 | 0 | 443 | 0 | 0 |
| 2012Youpei Wang | N370S | 208 | 0 | 0 | 298 | 0 | 0 |
| 2013M de L Gonz´ alez-del Rinc´ on | N370S | 128 | 0 | 0 | 252 | 0 | 0 |
| 2013Raquel Duran | N370S | 180 | 4 | 1 | 282 | 1 | 0 |
| 2014Rosanna Asselta | N370S | 2281 | 69 | 0 | 1107 | 4 | 0 |
| 2014Teeratorn Pulkes | N370S | 480 | 0 | 0 | 395 | 0 | 0 |
| 2014Yuanzhe Li | N370S | 147 | 0 | 0 | 100 | 0 | 0 |
| 2014Zhe Yu | N370S | 184 | 0 | 0 | 130 | 0 | 0 |
| 2015E. Dagan | N370S | 233(233) * | 47(47)* | 7 | 386(386) * | 14(14)* | 0 |
| 2015Fabin Han | N370S | 223 | 2 | 0 | 110 | 0 | 0 |
| 2016Caroline Ran | N370S | 1566 | 10 | 0 | 1920 | 2 | 0 |
| 2016David Crosiers | N370S | 263 | 3 | 0 | 535 | 1 | 0 |
| 2016David Crosiers | N370S | 266 | 0 | 0 | 536 | 0 | 0 |
| 2016Silvia Jesu´s | N370S | 527 | 5 | 0 | 542 | 0 | 0 |
| 2017Melinda Barkhuizen | N370S | 104 | 1 | 0 | 40 | 0 | 0 |
| 2017Thomas R. Barber | N370S | 105 | 1 | 0 | 282 | 1 | 0 |
| 2006M. Toft | N370S | 307 | 4 | 0 | 467 | 7 | 0 |
| 2007Elvira V. De Marco | N370S | 392 | 3 | 0 | 483 | 0 | 0 |
| 2007Shira G. Ziegler | N370S | 92 | 0 | 0 | 92 | 0 | 0 |
| 2008Ignacio F. Mata | N370S | 710 | 11 | 0 | 552 | 2 | 0 |
| 2013Michael A. Nalls | N370S | 151 | 0 | 0 | 1962 | 0 | 0 |
| 2016Rita Török | N370S | 124 | 0 | 0 | 122 | 0 | 0 |
| 2007Jose Bras | E388K | 230 | 0 | 0 | 428 | 2 | 0 |
| 2011Suzanne Lesage(1) | E388K | 1390 | 1 | 0 | 390 | 1 | 0 |
| 2012K. R. Kumar | E388K | 360 | 0 | 0 | 347 | 1 | 0 |
| 2013Raquel Duran | E388K | 184 | 1 | 0 | 283 | 0 | 0 |
| 2013Michael A. Nalls | E388K | 151 | 0 | 0 | 1961 | 1 | 0 |
| 2014Rosanna Asselta | E388K | 2345 | 5 | 0 | 1110 | 1 | 0 |
| 2007L.N. Clark | E388K | 278(178) * | 0 | 0 | 179(85) * | 0 | 0 |
| 2007Shira G. Ziegler | E388K | 92 | 0 | 0 | 92 | 0 | 0 |
| 2009Kallirhoe Kalinderi | E388K | 172 | 0 | 0 | 132 | 0 | 0 |
| 2009Jun Mitsui | E388K | 534 | 0 | 0 | 544 | 0 | 0 |
| 2009Juliane Neumann | E388K | 790 | 0 | 0 | 257 | 0 | 0 |
| 2011S. Lesage(2) | E388K | 194 | 0 | 0 | 177 | 0 | 0 |
| 2011Nu´ ria Seto´ -Salvia, BS | E388K | 225 | 0 | 0 | 186 | 0 | 0 |
| 2012Jung Mi Choi | E388K | 277 | 0 | 0 | 100 | 0 | 0 |
| 2014Teeratorn Pulkes | E388K | 480 | 0 | 0 | 395 | 0 | 0 |
| 2014Zhe Yu | E388K | 184 | 0 | 0 | 130 | 0 | 0 |
| 2014Yuanzhe Li | E388K | 147 | 0 | 0 | 100 | 0 | 0 |
| 2015Fabin Han | E388K | 225 | 0 | 0 | 110 | 0 | 0 |
| 2016David Crosiers | E388K | 266 | 0 | 0 | 536 | 0 | 0 |
| 2016Silvia Jesu´s | E388K | 532 | 0 | 0 | 542 | 0 | 0 |
| 2007Jose Bras | D409H | 229 | 1 | 0 | 430 | 0 | 0 |
| 2007L.N. Clark | D409H | 277(178) * | 1 | 0 | 179(85) * | 0 | 0 |
| 2007Shira G. Ziegler | D409H | 91 | 1 | 0 | 92 | 0 | 0 |
| 2008Usha Gutt | D409H | 183 | 1 | 0 | 91 | 1 | 0 |
| 2008Z. Gan-Or | D409H | 418 | 2 | 0 | 4138 | 0 | 0 |
| 2009Juliane Neumann | D409H | 789 | 1 | 0 | 257 | 0 | 0 |
| 2009Jun Mitsui | D409H | 534 | 0 | 0 | 544 | 0 | 0 |
| 2009Kallirhoe Kalinderi | D409H | 171 | 1 | 0 | 132 | 0 | 0 |
| 2011C.-L. Huang | D409H | 965 | 2 | 0 | 780 | 0 | 0 |
| 2011Marina Moraitou | D409H | 198 | 7 | 0 | 206 | 0 | 0 |
| 2011Nu´ ria Seto´ -Salvia | D409H | 223 | 2 | 0 | 186 | 0 | 0 |
| 2011S. Lesage(2) | D409H | 194 | 0 | 0 | 177 | 0 | 0 |
| 2011Suzanne Lesage(1) | D409H | 1390 | 1 | 0 | 391 | 0 | 0 |
| 2012Jung Mi Choi | D409H | 277 | 0 | 0 | 100 | 0 | 0 |
| 2012K. R. Kumar | D409H | 354 | 6 | 0 | 346 | 2 | 0 |
| 2013Raquel Duran | D409H | 184 | 1 | 0 | 283 | 0 | 0 |
| 2014Teeratorn Pulkes | D409H | 480 | 0 | 0 | 395 | 0 | 0 |
| 2014Yuanzhe Li | D409H | 143 | 4 | 0 | 100 | 0 | 0 |
| 2014Zhe Yu | D409H | 184 | 0 | 0 | 130 | 0 | 0 |
| 2015Fabin Han | D409H | 225 | 0 | 0 | 110 | 0 | 0 |
| 2016David Crosiers | D409H | 266 | 0 | 0 | 536 | 0 | 0 |
| 2016Silvia Jesu´s | D409H | 532 | 0 | 0 | 542 | 0 | 0 |
| 2007Jose Bras | D443N | 230 | 0 | 0 | 430 | 0 | 0 |
| 2007L.N. Clark | D443N | 278(178) * | 0 | 0 | 179(85) * | 0 | 0 |
| 2007Shira G. Ziegler | D443N | 92 | 0 | 0 | 92 | 0 | 0 |
| 2009Juliane Neumann | D443N | 789 | 1 | 0 | 257 | 0 | 0 |
| 2009Jun Mitsui | D443N | 534 | 0 | 0 | 544 | 0 | 0 |
| 2009Kallirhoe Kalinderi | D443N | 172 | 0 | 0 | 132 | 0 | 0 |
| 2011Nu´ ria Seto´ -Salvia, BS | D443N | 225 | 0 | 0 | 186 | 0 | 0 |
| 2011S. Lesage(2) | D443N | 194 | 0 | 0 | 176 | 1 | 0 |
| 2011Suzanne Lesage(1) | D443N | 1291 | 0 | 0 | 391 | 0 | 0 |
| 2012Jung Mi Choi | D443N | 277 | 0 | 0 | 100 | 0 | 0 |
| 2013Michael A. Nalls | D443N | 151 | 0 | 0 | 1960 | 2 | 0 |
| 2013Raquel Duran | D443N | 185 | 0 | 0 | 283 | 0 | 0 |
| 2013Raquel Duran | D443N | 185 | 0 | 0 | 283 | 0 | 0 |
| 2014Rosanna Asselta | D443N | 2349 | 1 | 0 | 1111 | 0 | 0 |
| 2014Teeratorn Pulkes | D443N | 480 | 0 | 0 | 395 | 0 | 0 |
| 2014Yuanzhe Li | D443N | 147 | 0 | 0 | 100 | 0 | 0 |
| 2014Zhe Yu | D443N | 184 | 0 | 0 | 130 | 0 | 0 |
| 2015Fabin Han | D443N | 225 | 0 | 0 | 110 | 0 | 0 |
| 2016David Crosiers | D443N | 266 | 0 | 0 | 536 | 0 | 0 |
| 2016Silvia Jesu´s | D443N | 532 | 0 | 0 | 542 | 0 | 0 |
| 2005Christine Sato | L444P | 87 | 1 | 0 | 122 | 0 | 0 |
| 2006M. Toft | L444P | 308 | 3 | 0 | 473 | 1 | 0 |
| 2007Elvira V. De Marco | L444P | 387 | 8 | 0 | 482 | 1 | 0 |
| 2007Eng-King Tan | L444P | 323 | 8 | 0 | 347 | 0 | 0 |
| 2007Jose Bras | L444P | 227 | 3 | 0 | 430 | 0 | 0 |
| 2007L.N. Clark | L444P | 276(178) * | 2 | 0 | 179(85) * | 0 | 0 |
| 2007Mariana Spitz | L444P | 63 | 2 | 0 | 267 | 0 | 0 |
| 2007Shira G. Ziegler | L444P | 91 | 1 | 0 | 92 | 0 | 0 |
| 2007Yih-Ru Wu | L444P | 505 | 13 | 0 | 337 | 2 | 0 |
| 2008Ignacio F. Mata | L444P | 711 | 10 | 0 | 553 | 1 | 0 |
| 2008Usha Gutt | L444P | 180 | 4 | 0 | 91 | 1 | 0 |
| 2008W.C. Nichols | L444P | 441 | 9 | 0 | 359 | 0 | 0 |
| 2008Z. Gan-Or | L444P | 418 | 2 | 0 | 4134 | 4 | 0 |
| 2009Juliane Neumann | L444P | 779 | 11 | 0 | 257 | 0 | 0 |
| 2009Jun Mitsui | L444P | 526 | 8 | 0 | 544 | 0 | 0 |
| 2009Kallirhoe Kalinderi | L444P | 170 | 2 | 0 | 132 | 0 | 0 |
| 2010Adriana Vaz dos Santos | L444P | 108 | 2 | 0 | 155 | 0 | 0 |
| 2010X.-Y. Mao | L444P | 596 | 20 | 0 | 410 | 1 | 0 |
| 2011C.-L. Huang | L444P | 940 | 27 | 0 | 779 | 1 | 0 |
| 2011Marina Moraitou | L444P | 199 | 6 | 0 | 205 | 1 | 0 |
| 2011Nu´ ria Seto´ -Salvia, BS | L444P | 219 | 6 | 0 | 186 | 0 | 0 |
| 2011Qi-Ying Sun | L444P | 391 | 11 | 0 | 413 | 0 | 0 |
| 2011S. Lesage(2) | L444P | 192 | 2 | 0 | 177 | 0 | 0 |
| 2011Suzanne Lesage(1) | L444P | 1372 | 18 | 1 | 391 | 0 | 0 |
| 2011Suzanne Lesage(1) | L444P | 1291 | 0 | 0 | 391 | 0 | 0 |
| 2012Anton Emelyanov | L444P | 324 | 6 | 0 | 239 | 2 | 0 |
| 2012Beatriz de Carvalho Guimarães | L444P | 231 | 6 | 0 | 186 | 0 | 0 |
| 2012Jung Mi Choi | L444P | 275 | 2 | 0 | 291 | 0 | 0 |
| 2012K. R. Kumar | L444P | 358 | 2 | 0 | 347 | 1 | 0 |
| 2012Xiong Zhang | L444P | 189 | 6 | 0 | 443 | 0 | 0 |
| 2012Youpei Wang | L444P | 200 | 7 | 1 | 297 | 1 | 0 |
| 2013M de L Gonz´ alez-del Rinc´ on | L444P | 121 | 7 | 0 | 252 | 0 | 0 |
| 2013Michael A. Nalls | L444P | 148 | 3 | 0 | 1959 | 3 | 0 |
| 2013Raquel Duran | L444P | 183 | 2 | 0 | 283 | 0 | 0 |
| 2014Rosanna Asselta | L444P | 2303 | 47 | 0 | 1108 | 3 | 0 |
| 2014Teeratorn Pulkes | L444P | 465 | 15 | 0 | 394 | 1 | 0 |
| 2014Yuanzhe Li | L444P | 135 | 12 | 0 | 100 | 0 | 0 |
| 2014Zhe Yu | L444P | 182 | 2 | 0 | 130 | 0 | 0 |
| 2015E.Dagan | L444P | 287(287) * | 0 | 0 | 400(400) * | 0 | 0 |
| 2015Fabin Han | L444P | 221 | 4 | 0 | 110 | 0 | 0 |
| 2016Caroline Ran | L444P | 1554 | 35 | 0 | 1964 | 3 | 0 |
| 2016David Crosiers | L444P | 263 | 3 | 0 | 535 | 1 | 0 |
| 2016Rita Török | L444P | 121 | 3 | 0 | 122 | 0 | 0 |
| 2016Silvia Jesu´s | L444P | 519 | 13 | 0 | 536 | 6 | 0 |
| 2017Thomas R. Barber | L444P | 106 | 0 | 0 | 283 | 0 | 0 |
| 2007Jose Bras | V460V/M/L | 230 | 0 | 0 | 430 | 0 | 0 |
| 2007L.N. Clark | V460V/M/L | 278(178) * | 0 | 0 | 179(85) * | 0 | 0 |
| 2007Shira G. Ziegler | V460M | 92 | 0 | 0 | 91 | 1 | 0 |
| 2008Usha Gutt | V460M | 184 | 0 | 0 | 91 | 1 | 0 |
| 2009Juliane Neumann | V460V/M/L | 790 | 0 | 0 | 257 | 0 | 0 |
| 2009Jun Mitsui | V460V | 532 | 2 | 0 | 543 | 1 | 0 |
| 2009Kallirhoe Kalinderi | V460L | 172 | 0 | 0 | 130 | 2 | 0 |
| 2011Nu´ ria Seto´ -Salvia, BS | V460V/M/L | 225 | 0 | 0 | 186 | 0 | 0 |
| 2011S. Lesage(2) | V460L | 193 | 1 | 0 | 177 | 0 | 0 |
| 2011S. Lesage(2) | V460V/M/L | 194 | 0 | 0 | 177 | 0 | 0 |
| 2011Suzanne Lesage(1) | V460L | 1390 | 1 | 0 | 391 | 0 | 0 |
| 2012Jung Mi Choi | V460V/M/L | 277 | 0 | 0 | 100 | 0 | 0 |
| 2013Raquel Duran | V460V/M/L | 185 | 0 | 0 | 283 | 0 | 0 |
| 2014Teeratorn Pulkes | V460V/M/L | 480 | 0 | 0 | 395 | 0 | 0 |
| 2014Yuanzhe Li | V460V/M/L | 147 | 0 | 0 | 100 | 0 | 0 |
| 2014Zhe Yu | V460V/M/L | 184 | 0 | 0 | 130 | 0 | 0 |
| 2015Fabin Han | V460V/M/L | 225 | 0 | 0 | 110 | 0 | 0 |
| 2016David Crosiers | V460V/M/L | 266 | 0 | 0 | 536 | 0 | 0 |
| 2016Silvia Jesu´s | V460V/M/L | 532 | 0 | 0 | 542 | 0 | 0 |
| 2007Jose Bras | R463C | 230 | 0 | 0 | 430 | 0 | 0 |
| 2007L.N. Clark | R463C | 278(178) * | 0 | 0 | 179(85) * | 0 | 0 |
| 2007Shira G. Ziegler | R463C | 92 | 0 | 0 | 92 | 0 | 0 |
| 2009Juliane Neumann | R463C | 787 | 3 | 0 | 257 | 0 | 0 |
| 2009Jun Mitsui | R463C | 534 | 0 | 0 | 544 | 0 | 0 |
| 2009Kallirhoe Kalinderi | R463C | 172 | 0 | 0 | 132 | 0 | 0 |
| 2011Nu´ ria Seto´ -Salvia, BS | R463C | 225 | 0 | 0 | 186 | 0 | 0 |
| 2011S. Lesage(2) | R463C | 194 | 0 | 0 | 177 | 0 | 0 |
| 2011Suzanne Lesage(1) | R463C | 1389 | 2 | 0 | 391 | 0 | 0 |
| 2012K. R. Kumar | R463C | 359 | 1 | 0 | 348 | 0 | 0 |
| 2013Michael A. Nalls | R463C | 151 | 0 | 0 | 1961 | 1 | 0 |
| 2013Raquel Duran | R463C | 182 | 3 | 0 | 283 | 0 | 0 |
| 2014Teeratorn Pulkes | R463C | 480 | 0 | 0 | 395 | 0 | 0 |
| 2014Yuanzhe Li | R463C | 147 | 0 | 0 | 100 | 0 | 0 |
| 2014Zhe Yu | R463C | 184 | 0 | 0 | 130 | 0 | 0 |
| 2015Fabin Han | R463C | 225 | 0 | 0 | 110 | 0 | 0 |
| 2016David Crosiers | R463C | 266 | 0 | 0 | 536 | 0 | 0 |
| 2016Silvia Jesu´s | R463C | 532 | 0 | 0 | 542 | 0 | 0 |
| 2004Judith Aharon-Peretz | R496H | 98 | 1 | 0 | 1543 | 0 | 0 |
| 2007Jose Bras | R496H | 230 | 0 | 0 | 430 | 0 | 0 |
| 2007L.N. Clark | R496H | 277(178)* | 1(1)* | 0 | 179(85) * | 0 | 0 |
| 2007Shira G. Ziegler | R496H | 92 | 0 | 0 | 92 | 0 | 0 |
| 2008Z. Gan-Or | R496H | 411 | 9 | 0 | 4137 | 1 | 0 |
| 2009Juliane Neumann | R496H | 790 | 0 | 0 | 257 | 0 | 0 |
| 2009Jun Mitsui | R496H | 534 | 0 | 0 | 544 | 0 | 0 |
| 2009Jun Mitsui | R496H | 534 | 0 | 0 | 544 | 0 | 0 |
| 2009Kallirhoe Kalinderi | R496H | 172 | 0 | 0 | 132 | 0 | 0 |
| 2011Nu´ ria Seto´ -Salvia, BS | R496H | 225 | 0 | 0 | 186 | 0 | 0 |
| 2011S. Lesage(2) | R496H | 194 | 0 | 0 | 177 | 0 | 0 |
| 2011Suzanne Lesage(1) | R496H | 1291 | 0 | 0 | 391 | 0 | 0 |
| 2013Raquel Duran | R496H | 185 | 0 | 0 | 283 | 0 | 0 |
| 2014Teeratorn Pulkes | R496H | 480 | 0 | 0 | 395 | 0 | 0 |
| 2014Yuanzhe Li | R496H | 147 | 0 | 0 | 100 | 0 | 0 |
| 2014Zhe Yu | R496H | 184 | 0 | 0 | 130 | 0 | 0 |
| 2015E. Dagan | R496H | 283(283) * | 4(4) * | 0 | 397(397) * | 3(3) * | 0 |
| 2015Fabin Han | R496H | 225 | 0 | 0 | 110 | 0 | 0 |
| 2015Fabin Han | R496H | 225 | 0 | 0 | 110 | 0 | 0 |
| 2016David Crosiers | R496H | 266 | 0 | 0 | 536 | 0 | 0 |
| 2016Silvia Jesu´s | R496H | 532 | 0 | 0 | 542 | 0 | 0 |
| 2007Jose Bras | Q497R | 230 | 0 | 0 | 430 | 0 | 0 |
| 2007L.N. Clark | Q497R | 278(178)* | 0 | 0 | 179(85) * | 0 | 0 |
| 2007Shira G. Ziegler | Q497R | 91 | 1 | 0 | 92 | 0 | 0 |
| 2008Usha Gutt | Q497R | 183 | 1 | 0 | 91 | 1 | 0 |
| 2009Juliane Neumann | Q497R | 790 | 0 | 0 | 257 | 0 | 0 |
| 2009Jun Mitsui | Q497R | 534 | 0 | 0 | 544 | 0 | 0 |
| 2009Kallirhoe Kalinderi | Q497R | 172 | 0 | 0 | 132 | 0 | 0 |
| 2011C.-L. Huang | Q497R | 967 | 0 | 0 | 780 | 0 | 0 |
| 2011Marina Moraitou | Q497R | 205 | 0 | 0 | 206 | 0 | 0 |
| 2011Nu´ ria Seto´ -Salvia, BS | Q497R | 225 | 0 | 0 | 186 | 0 | 0 |
| 2011S. Lesage(2) | Q497R | 194 | 0 | 0 | 177 | 0 | 0 |
| 2011Suzanne Lesage(1) | Q497R | 1291 | 0 | 0 | 391 | 0 | 0 |
| 2012Jung Mi Choi | Q497R | 277 | 0 | 0 | 100 | 0 | 0 |
| 2013Raquel Duran | Q497R | 185 | 0 | 0 | 283 | 0 | 0 |
| 2014Teeratorn Pulkes | Q497R | 480 | 0 | 0 | 395 | 0 | 0 |
| 2014Yuanzhe Li | Q497R | 147 | 0 | 0 | 100 | 0 | 0 |
| 2014Zhe Yu | Q497R | 183 | 1 | 0 | 130 | 0 | 0 |
| 2015Fabin Han | Q497R | 225 | 0 | 0 | 110 | 0 | 0 |
| 2016David Crosiers | Q497R | 266 | 0 | 0 | 536 | 0 | 0 |
| 2016Silvia Jesu´s | Q497R | 532 | 0 | 0 | 542 | 0 | 0 |
| 2007Jose Bras | RecNciI# | 230 | 0 | NA | 430 | 0 | NA |
| 2007L.N. Clark | RecNciI# | 277(178)* | 1(1)* | NA | 179(85) * | 0 | NA |
| 2007Shira G. Ziegler | RecNciI# | 92 | 0 | NA | 92 | 0 | NA |
| 2007Yih-Ru Wu | RecNciI# | 516 | 2 | NA | 337 | 2 | NA |
| 2008W.C. Nichols | RecNciI# | 446 | 4 | NA | 358 | 1 | NA |
| 2009Juliane Neumann | RecNciI# | 788 | 2 | NA | 257 | 0 | NA |
| 2009Jun Mitsui | RecNciI# | 520 | 14 | NA | 542 | 2 | NA |
| 2009Kallirhoe Kalinderi | RecNciI# | 172 | 0 | NA | 132 | 0 | NA |
| 2010Adriana Vaz dos Santos | RecNciI# | 109 | 1 | NA | 155 | 0 | NA |
| 2011C.-L. Huang | RecNciI# | 960 | 7 | NA | 779 | 1 | NA |
| 2011Nu´ ria Seto´ -Salvia, BS | RecNciI# | 224 | 1 | NA | 186 | 0 | NA |
| 2011S. Lesage(2) | RecNciI# | 192 | 2 | NA | 177 | 0 | NA |
| 2011Suzanne Lesage(1) | RecNciI# | 1389 | 2 | NA | 391 | 0 | NA |
| 2012Jung Mi Choi | RecNciI# | 277 | 0 | NA | 100 | 0 | NA |
| 2012K. R. Kumar | RecNciI# | 359 | 1 | NA | 348 | 0 | NA |
| 2013Michael A. Nalls | RecNciI# | 151 | 0 | NA | 1959 | 3 | NA |
| 2013Raquel Duran | RecNciI# | 182 | 3 | NA | 283 | 0 | NA |
| 2014Teeratorn Pulkes | RecNciI# | 480 | 0 | NA | 395 | 0 | NA |
| 2014Yuanzhe Li | RecNciI# | 146 | 1 | NA | 99 | 1 | NA |
| 2014Zhe Yu | RecNciI# | 181 | 3 | NA | 130 | 0 | NA |
| 2015Fabin Han | RecNciI# | 224 | 1 | NA | 109 | 0 | NA |
| 2016David Crosiers | RecNciI# | 265 | 1 | NA | 536 | 0 | NA |
| 2016Silvia Jesu´s | RecNciI# | 532 | 0 | NA | 542 | 0 | NA |
