# Supplementary material for: Integrated Genetic Analysis of Racial Differences of Common GBA Variants in Parkinson's Disease: A Meta-Analysis
Source: Front Mol Neurosci. 2018 Feb 15;11:43. doi: 10.3389/fnmol.2018.00043 (PMC5829555; doi:10.3389/fnmol.2018.00043)
Supplement: Supplementary file 4 [file DataSheet4.DOCX]

Supplementary Material

Integrated genetic analysis of racial differences of common GBA variants in parkinson's disease: a Meta-analysis

Yuan Zhang^1 †^, Li Shu^1 †^, Qiying Sun^2,3,4^, Xun Zhou^1^, Hongxu Pan^1^, Jifeng Guo^1, 3,4^, Beisha Tang^1, 3,4,5*^

**^†^** These authors have contributed equally to this work and are co-first authors.

^*^ Correspondence: Beisha Tang [bstang7398@163.com](mailto:bstang7398@163.com)

**Supplementary Table 4**: Frequency attributions of 18 *GBA* variants analyzed. Abbreviation: PD, Parkinson’s disease. AA, wild type. Aa, heterozygous carriers. Aa, homozygous carriers. MAF, minor allele frequency. *, RecNciI is recombinant allele which used AA described the wild type and Aa for the recombinant one (L444P-A456P-V460V). NA, not available. AJ, Ashkenazi Jewish. Non-AJ, non-Ashkenazi Jewish. GNOMAD, the Genome Aggregation Database (<http://gnomad.broadinstitute.org/>).

| Variants | Groups | genotype% of PD | | | genotype% of controls | | | MAF | | |
| --- | --- | --- | --- | --- | --- | --- | --- | --- | --- | --- |
|  |  | AA | Aa | aa | AA | Aa | aa | PD | controls | GNOMAD |
| 84insGG | total | 99.6170% | 0.3830% | 0.0000% | 99.8781% | 0.1219% | 0.0000% | 0.1915% | 0.0609% | - |
|  | AJ | 97.3577% | 2.6423% | 0.0000% | 99.8054% | 0.1946% | 0.0000% | 1.3211% | 0.0973% | - |
|  | NON-AJ | 100.0000% | 0.0000% | 0.0000% | 100.0000% | 0.0000% | 0.0000% | 0.0000% | 0.0000% | - |
|  | East Asians | 100.0000% | 0.0000% | 0.0000% | 100.0000% | 0.0000% | 0.0000% | 0.0000% | 0.0000% | - |
|  | European/West Asians | 100.0000% | 0.0000% | 0.0000% | 100.0000% | 0.0000% | 0.0000% | 0.0000% | 0.0000% | - |
|  | Hispanics | 100.0000% | 0.0000% | 0.0000% | 100.0000% | 0.0000% | 0.0000% | 0.0000% | 0.0000% | - |
|  | Mix | 100.0000% | 0.0000% | 0.0000% | 100.0000% | 0.0000% | 0.0000% | 0.0000% | 0.0000% | - |
|  | African | 100.0000% | 0.0000% | 0.0000% | 100.0000% | 0.0000% | 0.0000% | 0.0000% | 0.0000% | - |
| IVS2+1G＞A | total | 99.8727% | 0.1273% | 0.0000% | 99.9911% | 0.0089% | 0.0000% | 0.0636% | 0.0044% | 0.0068% |
|  | AJ | 99.5480% | 0.4520% | 0.0000% | 99.9784% | 0.0216% | 0.0000% | 0.2260% | 0.0108% | 0.0197% |
|  | NON-AJ | 99.9192% | 0.0808% | 0.0000% | 100.0000% | 0.0000% | 0.0000% | 0.0404% | 0.0000% | - |
|  | East Asians | 99.9417% | 0.0583% | 0.0000% | 100.0000% | 0.0000% | 0.0000% | 0.0292% | 0.0000% | 0.0106% |
|  | European/West Asians | 99.9219% | 0.0781% | 0.0000% | 100.0000% | 0.0000% | 0.0000% | 0.0390% | 0.0000% | 0.0087% |
|  | Hispanics | 99.0909% | 0.9091% | 0.0000% | 100.0000% | 0.0000% | 0.0000% | 0.4545% | 0.0000% | 0.0029% |
|  | Mix | 100.0000% | 0.0000% | 0.0000% | 100.0000% | 0.0000% | 0.0000% | 0.0000% | 0.0000% | - |
|  | African | 100.0000% | 0.0000% | 0.0000% | 100.0000% | 0.0000% | 0.0000% | 0.0000% | 0.0000% | 0.0000% |
| R120W | total | 99.6634% | 0.3366% | 0.0000% | 100.0000% | 0.0000% | 0.0000% | 0.1683% | 0.0000% | 0.0008% |
|  | AJ | 100.0000% | 0.0000% | 0.0000% | 100.0000% | 0.0000% | 0.0000% | 0.0000% | 0.0000% | 0.0000% |
|  | NON-AJ | 99.6561% | 0.3439% | 0.0000% | 100.0000% | 0.0000% | 0.0000% | 0.1720% | 0.0000% | - |
|  | East Asians | 99.3059% | 0.6941% | 0.0000% | 100.0000% | 0.0000% | 0.0000% | 0.3470% | 0.0000% | 0.0000% |
|  | European/West Asians | 99.9341% | 0.0659% | 0.0000% | 100.0000% | 0.0000% | 0.0000% | 0.0330% | 0.0000% | 0.0000% |
|  | Hispanics | 100.0000% | 0.0000% | 0.0000% | 100.0000% | 0.0000% | 0.0000% | 0.0000% | 0.0000% | 0.0030% |
|  | Mix | 99.6923% | 0.3077% | 0.0000% | 100.0000% | 0.0000% | 0.0000% | 0.1538% | 0.0000% | - |
|  | African | 100.0000% | 0.0000% | 0.0000% | 100.0000% | 0.0000% | 0.0000% | 0.0000% | 0.0000% | 0.0000% |
| R131C | total | 99.9033% | 0.0806% | 0.0161% | 100.0000% | 0.0000% | 0.0000% | 0.0564% | 0.0000% | 0.0020% |
|  | AJ | 100.0000% | 0.0000% | 0.0000% | 100.0000% | 0.0000% | 0.0000% | 0.0000% | 0.0000% | 0.0000% |
|  | NON-AJ | 99.9004% | 0.0830% | 0.0166% | 100.0000% | 0.0000% | 0.0000% | 0.0581% | 0.0000% | 0.0000% |
|  | East Asians | 99.9417% | 0.0583% | 0.0000% | 100.0000% | 0.0000% | 0.0000% | 0.0292% | 0.0000% | 0.0000% |
|  | European/West Asians | 99.9209% | 0.0791% | 0.0000% | 100.0000% | 0.0000% | 0.0000% | 0.0396% | 0.0000% | 0.0018% |
|  | Hispanics | 100.0000% | 0.0000% | 0.0000% | 100.0000% | 0.0000% | 0.0000% | 0.0000% | 0.0000% | 0.0018% |
|  | Mix | 100.0000% | 0.0000% | 0.0000% | 100.0000% | 0.0000% | 0.0000% | 0.0000% | 0.0000% | 0.0018% |
|  | African | 98.9691% | 0.5155% | 0.5155% | 100.0000% | 0.0000% | 0.0000% | 0.7732% | 0.0000% | 0.0000% |
| R163Q | total | 99.8886% | 0.1114% | 0.0000% | 99.8562% | 0.1438% | 0.0000% | 0.0557% | 0.0719% | 0.0222% |
|  | AJ | 100.0000% | 0.0000% | 0.0000% | 100.0000% | 0.0000% | 0.0000% | 0.0000% | 0.0000% | 0.0000% |
|  | NON-AJ | 99.8854% | 0.1146% | 0.0000% | 99.8536% | 0.1464% | 0.0000% | 0.0573% | 0.0732% | - |
|  | East Asians | 99.6312% | 0.3688% | 0.0000% | 99.5742% | 0.4258% | 0.0000% | 0.1844% | 0.2129% | 0.2905% |
|  | European/West Asians | 100.0000% | 0.0000% | 0.0000% | 100.0000% | 0.0000% | 0.0000% | 0.0000% | 0.0000% | 0.0018% |
|  | Hispanics | 100.0000% | 0.0000% | 0.0000% | 100.0000% | 0.0000% | 0.0000% | 0.0000% | 0.0000% | 0.0035% |
|  | Mix | 100.0000% | 0.0000% | 0.0000% | 100.0000% | 0.0000% | 0.0000% | 0.0000% | 0.0000% | - |
|  | African | 100.0000% | 0.0000% | 0.0000% | 100.0000% | 0.0000% | 0.0000% | 0.0000% | 0.0000% | 0.0000% |
| H255Q | total | 99.7300% | 0.2700% | 0.0000% | 99.9416% | 0.0584% | 0.0000% | 0.1350% | 0.0292% | 0.0220% |
|  | AJ | 100.0000% | 0.0000% | 0.0000% | 100.0000% | 0.0000% | 0.0000% | 0.0000% | 0.0000% | 0.0493% |
|  | NON-AJ | 99.7226% | 0.2774% | 0.0000% | 99.9406% | 0.0594% | 0.0000% | 0.1387% | 0.0297% | - |
|  | East Asians | 100.0000% | 0.0000% | 0.0000% | 100.0000% | 0.0000% | 0.0000% | 0.0000% | 0.0000% | 0.0000% |
|  | European/West Asians | 99.5771% | 0.4229% | 0.0000% | 99.9094% | 0.0906% | 0.0000% | 0.2115% | 0.0453% | 0.0308% |
|  | Hispanics | 100.0000% | 0.0000% | 0.0000% | 100.0000% | 0.0000% | 0.0000% | 0.0000% | 0.0000% | 0.0116% |
|  | Mix | 100.0000% | 0.0000% | 0.0000% | 100.0000% | 0.0000% | 0.0000% | 0.0000% | 0.0000% | - |
|  | African | 100.0000% | 0.0000% | 0.0000% | 100.0000% | 0.0000% | 0.0000% | 0.0000% | 0.0000% | 0.0000% |
| E326K | total | 97.1686% | 2.7378% | 0.0936% | 98.3670% | 1.6330% | 0.0000% | 1.4625% | 0.8165% | 1.0910% |
|  | AJ | 99.4382% | 0.5618% | 0.0000% | 98.8235% | 1.1765% | 0.0000% | 0.2809% | 0.5882% | 0.5713% |
|  | NON-AJ | 97.1203% | 2.7841% | 0.0956% | 98.3618% | 1.6382% | 0.0000% | 1.4876% | 0.8191% | - |
|  | East Asians | 98.8915% | 1.1085% | 0.0000% | 100.0000% | 0.0000% | 0.0000% | 0.5543% | 0.0000% | 0.0053% |
|  | European/West Asians | 96.6642% | 3.1907% | 0.1450% | 97.8903% | 2.1097% | 0.0000% | 1.7404% | 1.0549% | 1.2340% |
|  | Hispanics | 98.4615% | 1.5385% | 0.0000% | 100.0000% | 0.0000% | 0.0000% | 0.7692% | 0.0000% | 0.2150% |
|  | Mix | 95.8710% | 4.1290% | 0.0000% | 97.3404% | 2.6596% | 0.0000% | 2.0645% | 1.3298% | - |
|  | African | 98.3278% | 1.6722% | 0.0000% | 99.5392% | 0.4608% | 0.0000% | 0.8361% | 0.2304% | 0.2206% |
| T369M | total | 98.8664% | 1.0926% | 0.0410% | 99.2776% | 0.7224% | 0.0000% | 0.5873% | 0.3612% | 0.0061% |
|  | AJ | 99.4382% | 0.5618% | 0.0000% | 97.6471% | 2.3529% | 0.0000% | 0.2809% | 1.1765% | 0.0023% |
|  | NON-AJ | 98.8522% | 1.1058% | 0.0420% | 99.3030% | 0.6970% | 0.0000% | 0.5949% | 0.3485% | - |
|  | East Asians | 98.9498% | 1.0502% | 0.0000% | 100.0000% | 0.0000% | 0.0000% | 0.5251% | 0.0000% | 0.0000% |
|  | European/West Asians | 99.1506% | 0.7805% | 0.0689% | 99.1845% | 0.8155% | 0.0000% | 0.4591% | 0.4077% | 0.0094% |
|  | Hispanics | 100.0000% | 0.0000% | 0.0000% | 100.0000% | 0.0000% | 0.0000% | 0.0000% | 0.0000% | 0.0026% |
|  | Mix | 97.0323% | 2.9677% | 0.0000% | 98.4014% | 1.5986% | 0.0000% | 1.4839% | 0.7993% | - |
|  | African | 98.6622% | 1.3378% | 0.0000% | 99.0783% | 0.9217% | 0.0000% | 0.6689% | 0.4608% | 0.0008% |
| N370S | total | 97.6873% | 2.1760% | 0.1367% | 98.2633% | 1.7277% | 0.0090% | 1.2247% | 0.8728% | 0.2248% |
|  | AJ | 84.9913% | 13.6126% | 1.3962% | 94.4072% | 5.5609% | 0.0320% | 8.2024% | 2.8124% | 2.7190% |
|  | NON-AJ | 98.6155% | 1.3398% | 0.0447% | 99.7691% | 0.2309% | 0.0000% | 0.7146% | 0.1154% | - |
|  | East Asians | 99.7893% | 0.2107% | 0.0000% | 99.9335% | 0.0665% | 0.0000% | 0.1054% | 0.0333% | 0.0000% |
|  | European/West Asians | 98.2040% | 1.7393% | 0.0567% | 99.7480% | 0.2520% | 0.0000% | 0.9264% | 0.1260% | 0.2013% |
|  | Hispanics | 99.1453% | 0.6838% | 0.1709% | 100.0000% | 0.0000% | 0.0000% | 0.5128% | 0.0000% | 0.0872% |
|  | Mix | 98.7616% | 1.2384% | 0.0000% | 99.4835% | 0.5165% | 0.0000% | 0.6192% | 0.2583% | - |
|  | African | 99.4236% | 0.5764% | 0.0000% | 99.4907% | 0.5093% | 0.0000% | 0.2882% | 0.2547% | 0.0167% |
| E388K | total | 99.9240% | 0.0760% | 0.0000% | 99.9260% | 0.0740% | 0.0000% | 0.0380% | 0.0370% | 0.0002% |
|  | AJ | 100.0000% | 0.0000% | 0.0000% | 100.0000% | 0.0000% | 0.0000% | 0.0000% | 0.0000% | 0.0000% |
|  | NON-AJ | 99.9225% | 0.0775% | 0.0000% | 99.9252% | 0.0748% | 0.0000% | 0.0388% | 0.0374% | - |
|  | East Asians | 100.0000% | 0.0000% | 0.0000% | 100.0000% | 0.0000% | 0.0000% | 0.0000% | 0.0000% | 0.0000% |
|  | European/West Asians | 99.8948% | 0.1052% | 0.0000% | 99.9029% | 0.0971% | 0.0000% | 0.0526% | 0.0486% | 0.0003% |
|  | Hispanics | 100.0000% | 0.0000% | 0.0000% | 100.0000% | 0.0000% | 0.0000% | 0.0000% | 0.0000% | 0.0001% |
|  | Mix | 100.0000% | 0.0000% | 0.0000% | 100.0000% | 0.0000% | 0.0000% | 0.0000% | 0.0000% | - |
|  | African | 100.0000% | 0.0000% | 0.0000% | 100.0000% | 0.0000% | 0.0000% | 0.0000% | 0.0000% | 0.0000% |
| D409H | total | 99.6282% | 0.3718% | 0.0000% | 99.9704% | 0.0296% | 0.0000% | 0.1859% | 0.0148% | 0.0004% |
|  | AJ | 99.6656% | 0.3344% | 0.0000% | 100.0000% | 0.0000% | 0.0000% | 0.1672% | 0.0000% | 0.0000% |
|  | NON-AJ | 99.6253% | 0.3747% | 0.0000% | 99.9494% | 0.0506% | 0.0000% | 0.1873% | 0.0253% | - |
|  | East Asians | 99.7208% | 0.2792% | 0.0000% | 99.9552% | 0.0448% | 0.0000% | 0.1396% | 0.0224% | 0.0000% |
|  | European/West Asians | 99.5409% | 0.4591% | 0.0000% | 99.9396% | 0.0604% | 0.0000% | 0.2296% | 0.0302% | 0.0009% |
|  | Hispanics | 100.0000% | 0.0000% | 0.0000% | 100.0000% | 0.0000% | 0.0000% | 0.0000% | 0.0000% | 0.0000% |
|  | Mix | 99.6923% | 0.3077% | 0.0000% | 100.0000% | 0.0000% | 0.0000% | 0.1538% | 0.0000% | - |
|  | African | 100.0000% | 0.0000% | 0.0000% | 100.0000% | 0.0000% | 0.0000% | 0.0000% | 0.0000% | 0.0000% |
| D443N | total | 99.9772% | 0.0228% | 0.0000% | 99.9622% | 0.0378% | 0.0000% | 0.0114% | 0.0189% | 0.0701% |
|  | AJ | 100.0000% | 0.0000% | 0.0000% | 100.0000% | 0.0000% | 0.0000% | 0.0000% | 0.0000% | 0.0000% |
|  | NON-AJ | 99.9768% | 0.0232% | 0.0000% | 99.9618% | 0.0382% | 0.0000% | 0.0116% | 0.0191% | - |
|  | East Asians | 100.0000% | 0.0000% | 0.0000% | 100.0000% | 0.0000% | 0.0000% | 0.0000% | 0.0000% | 0.0000% |
|  | European/West Asians | 99.9686% | 0.0314% | 0.0000% | 99.9673% | 0.0327% | 0.0000% | 0.0157% | 0.0164% | 0.0000% |
|  | Hispanics | 100.0000% | 0.0000% | 0.0000% | 100.0000% | 0.0000% | 0.0000% | 0.0000% | 0.0000% | 0.0203% |
|  | Mix | 100.0000% | 0.0000% | 0.0000% | 100.0000% | 0.0000% | 0.0000% | 0.0000% | 0.0000% | - |
|  | African | 100.0000% | 0.0000% | 0.0000% | 99.4350% | 0.5650% | 0.0000% | 0.0000% | 0.2825% | 0.7698% |
| L444P | total | 98.1238% | 1.8664% | 0.0098% | 99.8475% | 0.1525% | 0.0000% | 0.9430% | 0.0763% | 0.0008% |
|  | AJ | 99.7740% | 0.2260% | 0.0000% | 99.9135% | 0.0865% | 0.0000% | 0.1130% | 0.0433% | 0.0000% |
|  | NON-AJ | 98.0488% | 1.9409% | 0.0103% | 99.8308% | 0.1692% | 0.0000% | 0.9807% | 0.0846% | - |
|  | East Asians | 97.3513% | 2.6324% | 0.0162% | 99.8598% | 0.1402% | 0.0000% | 1.3325% | 0.0701% | 0.0058% |
|  | European/West Asians | 98.4335% | 1.5579% | 0.0086% | 99.7833% | 0.2167% | 0.0000% | 0.7876% | 0.1083% | 0.0000% |
|  | Hispanics | 96.8519% | 3.1481% | 0.0000% | 100.0000% | 0.0000% | 0.0000% | 1.5741% | 0.0000% | 0.0030% |
|  | Mix | 98.3488% | 1.6512% | 0.0000% | 100.0000% | 0.0000% | 0.0000% | 0.8256% | 0.0000% | - |
|  | African | 98.9691% | 1.0309% | 0.0000% | 100.0000% | 0.0000% | 0.0000% | 0.5155% | 0.0000% | 0.0000% |
| V460L/M/V | total | 99.9392% | 0.0608% | 0.0000% | 99.8970% | 0.1030% | 0.0000% | 0.0304% | 0.0515% | 0.0137% |
|  | AJ | 100.0000% | 0.0000% | 0.0000% | 100.0000% | 0.0000% | 0.0000% | 0.0000% | 0.0000% | 0.0000% |
|  | NON-AJ | 99.9375% | 0.0625% | 0.0000% | 99.8951% | 0.1049% | 0.0000% | 0.0312% | 0.0524% | - |
|  | East Asians | 99.8946% | 0.1054% | 0.0000% | 99.7935% | 0.2065% | 0.0000% | 0.0527% | 0.1032% | 0.0424% |
|  | European/West Asians | 99.9736% | 0.0264% | 0.0000% | 99.9275% | 0.0725% | 0.0000% | 0.0132% | 0.0363% | 0.0127% |
|  | Hispanics | 100.0000% | 0.0000% | 0.0000% | 100.0000% | 0.0000% | 0.0000% | 0.0000% | 0.0000% | 0.0079% |
|  | Mix | 100.0000% | 0.0000% | 0.0000% | 100.0000% | 0.0000% | 0.0000% | 0.0000% | 0.0000% | - |
|  | African | 99.7423% | 0.2577% | 0.0000% | 100.0000% | 0.0000% | 0.0000% | 0.1289% | 0.0000% | 0.0069% |
| R463C | total | 99.8602% | 0.1398% | 0.0000% | 99.9853% | 0.0147% | 0.0000% | 0.0699% | 0.0074% | 0.0072% |
|  | AJ | 100.0000% | 0.0000% | 0.0000% | 100.0000% | 0.0000% | 0.0000% | 0.0000% | 0.0000% | 0.0000% |
|  | NON-AJ | 99.8562% | 0.1438% | 0.0000% | 99.9851% | 0.0149% | 0.0000% | 0.0719% | 0.0075% | - |
|  | East Asians | 100.0000% | 0.0000% | 0.0000% | 100.0000% | 0.0000% | 0.0000% | 0.0000% | 0.0000% | 0.0000% |
|  | European/West Asians | 99.7908% | 0.2092% | 0.0000% | 99.9803% | 0.0197% | 0.0000% | 0.1046% | 0.0099% | 0.0119% |
|  | Hispanics | 100.0000% | 0.0000% | 0.0000% | 100.0000% | 0.0000% | 0.0000% | 0.0000% | 0.0000% | 0.0000% |
|  | Mix | 100.0000% | 0.0000% | 0.0000% | 100.0000% | 0.0000% | 0.0000% | 0.0000% | 0.0000% | - |
|  | African | 100.0000% | 0.0000% | 0.0000% | 100.0000% | 0.0000% | 0.0000% | 0.0000% | 0.0000% | 0.0083% |
| R496H | total | 99.7970% | 0.2030% | 0.0000% | 99.9643% | 0.0357% | 0.0000% | 0.1015% | 0.0178% | 0.0012% |
|  | AJ | 98.4756% | 1.5244% | 0.0000% | 99.9351% | 0.0649% | 0.0000% | 0.7622% | 0.0324% | 0.0000% |
|  | NON-AJ | 100.0000% | 0.0000% | 0.0000% | 100.0000% | 0.0000% | 0.0000% | 0.0000% | 0.0000% | - |
|  | East Asians | 100.0000% | 0.0000% | 0.0000% | 100.0000% | 0.0000% | 0.0000% | 0.0000% | 0.0000% | 0.0524% |
|  | European/West Asians | 100.0000% | 0.0000% | 0.0000% | 100.0000% | 0.0000% | 0.0000% | 0.0000% | 0.0000% | 0.0000% |
|  | Hispanics | 100.0000% | 0.0000% | 0.0000% | 100.0000% | 0.0000% | 0.0000% | 0.0000% | 0.0000% | 0.0000% |
|  | Mix | 100.0000% | 0.0000% | 0.0000% | 100.0000% | 0.0000% | 0.0000% | 0.0000% | 0.0000% | 0.0000% |
|  | African | 100.0000% | 0.0000% | 0.0000% | 100.0000% | 0.0000% | 0.0000% | 0.0000% | 0.0000% | 0.0000% |
| Q497R | total | 99.9458% | 0.0542% | 0.0000% | 99.9801% | 0.0199% | 0.0000% | 0.0271% | 0.0100% | 0.0012% |
|  | AJ | 100.0000% | 0.0000% | 0.0000% | 100.0000% | 0.0000% | 0.0000% | 0.0000% | 0.0000% | 0.0000% |
|  | NON-AJ | 99.9440% | 0.0560% | 0.0000% | 99.9797% | 0.0203% | 0.0000% | 0.0280% | 0.0101% | - |
|  | East Asians | 99.8953% | 0.1047% | 0.0000% | 99.9552% | 0.0448% | 0.0000% | 0.0524% | 0.0224% | 0.0178% |
|  | European/West Asians | 100.0000% | 0.0000% | 0.0000% | 100.0000% | 0.0000% | 0.0000% | 0.0000% | 0.0000% | 0.0000% |
|  | Hispanics | 100.0000% | 0.0000% | 0.0000% | 100.0000% | 0.0000% | 0.0000% | 0.0000% | 0.0000% | 0.0000% |
|  | Mix | 100.0000% | 0.0000% | 0.0000% | 100.0000% | 0.0000% | 0.0000% | 0.0000% | 0.0000% | - |
|  | African | 100.0000% | 0.0000% | 0.0000% | 100.0000% | 0.0000% | 0.0000% | 0.0000% | 0.0000% | 0.0000% |
| RecNciI* | total | 99.4748% | 0.5252% | - | 99.8827% | 0.1173% | - | 0.2626% | 0.0586% | - |
|  | AJ | 100.0000% | 0.0000% | - | 100.0000% | 0.0000% | - | 0.0000% | 0.0000% | - |
|  | NON-AJ | 99.4639% | 0.5361% | - | 99.8815% | 0.1185% | - | 0.2681% | 0.0592% | - |
|  | East Asians | 99.1560% | 0.8440% | - | 99.7581% | 0.2419% | - | 0.4220% | 0.1210% | - |
|  | European/West Asians | 99.7675% | 0.2325% | - | 99.9408% | 0.0592% | - | 0.1162% | 0.0296% | - |
|  | Hispanics | 99.0909% | 0.9091% | - | 100.0000% | 0.0000% | - | 0.0000% | 0.0000% | - |
|  | Mix | 99.2258% | 0.7742% | - | 99.8221% | 0.1779% | - | 0.3871% | 0.0890% | - |
|  | African | 98.9691% | 1.0309% | - | 100.0000% | 0.0000% | - | 0.0000% | 0.0000% | - |
