# Supplementary material for: Integrated Genetic Analysis of Racial Differences of Common GBA Variants in Parkinson's Disease: A Meta-Analysis
Source: Front Mol Neurosci. 2018 Feb 15;11:43. doi: 10.3389/fnmol.2018.00043 (PMC5829555; doi:10.3389/fnmol.2018.00043)
Supplement: Supplementary file 5 [file Image1.PDF]

Supplementary Material

Integrated genetic analysis of racial differences of common GBA variants in Parkinson's disease: a Meta-analysis

Yuan Zhang[1 †], Li Shu[1 †], Qiying Sun[2,3,4], Xun Zhou[1], Hongxu Pan[1], Jifeng Guo[1, 3,4], Beisha Tang[1, 3,4,5*]

[†] These authors have contributed equally to this work and are co-first authors.

[*] **Correspondence: Beisha Tang bstang7398@163.com**

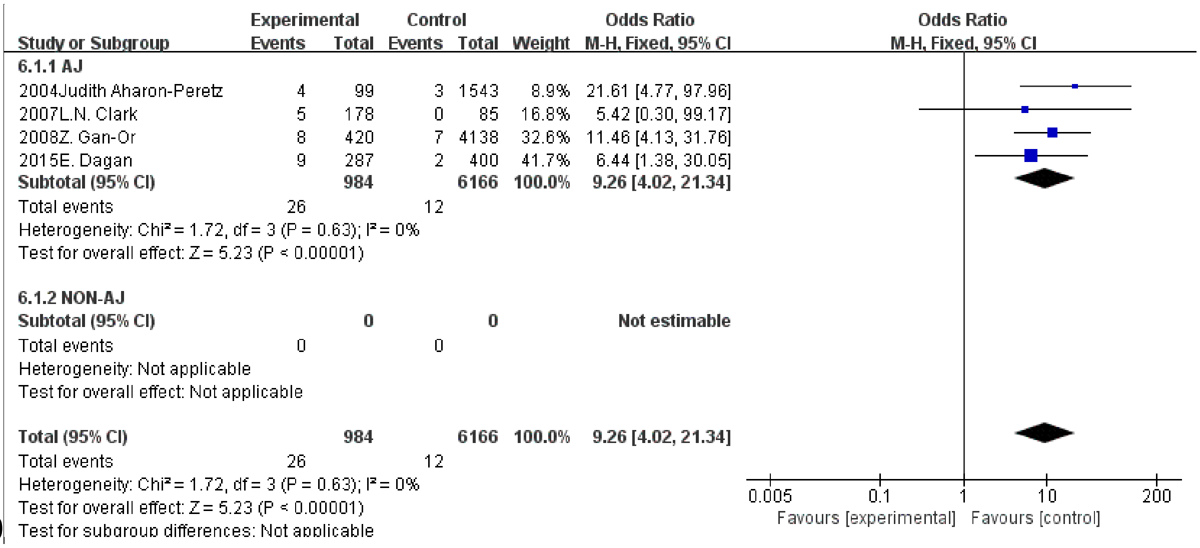

(A)

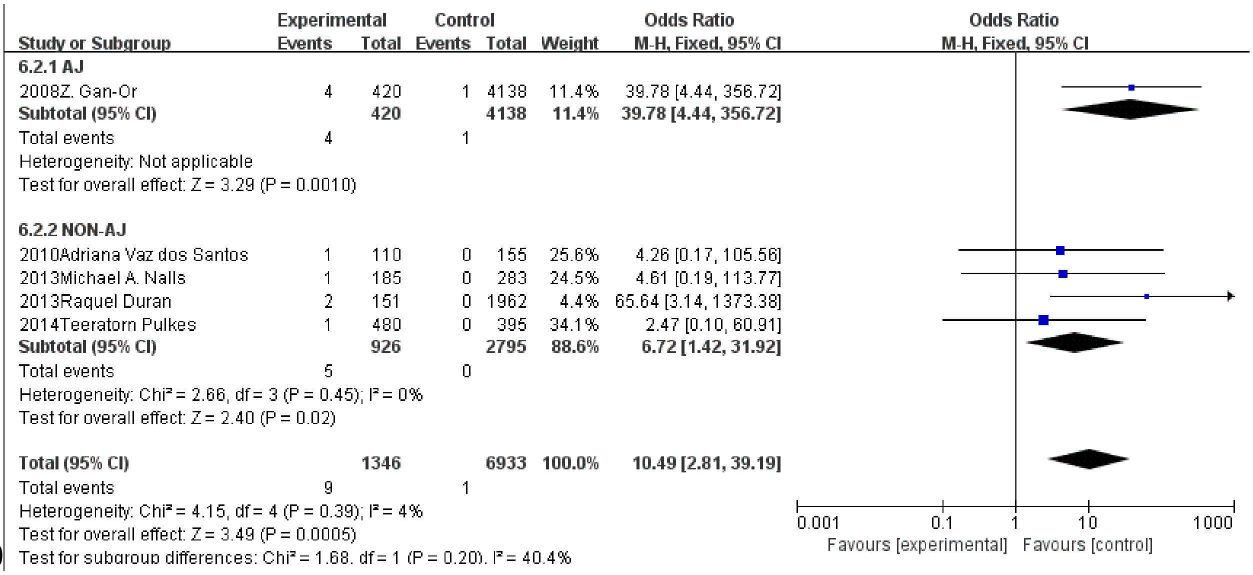

(B)

Study or Subgroup	Experimental Events	Experimental Total	Control Events	Control Total	Weight	Odds Ratio M-H, Fixed, 95% CI
**6.3.1 AJ**						
**Subtotal (95% CI)**		**0**		**0**		**Not estimable**
Total events	0		0			
Heterogeneity: Not applicable						
Test for overall effect: Not applicable						
**6.3.2 NON-AJ**						
2007Yih-Ru Wu	1	518	0	339	19.5%	1.97 [0.08, 48.45]
2009Jun Mitsui	15	534	0	544	15.6%	32.49 [1.94, 544.42]
2011Suzanne Lesage(1)	2	1391	0	391	25.2%	1.41 [0.07, 29.40]
2014Yuanzhe Li	9	147	0	100	18.0%	13.79 [0.79, 239.63]
2015Fabin Han	1	225	0	110	21.6%	1.48 [0.06, 36.54]
**Subtotal (95% CI)**		**2815**		**1484**	**100.0%**	**8.61 [2.46, 30.14]**
Total events	28		0			
Heterogeneity: Chi² = 4.30, df = 4 (P = 0.37); I² = 7%						
Test for overall effect: Z = 3.37 (P = 0.0008)						
**Total (95% CI)**		**2815**		**1484**	**100.0%**	**8.61 [2.46, 30.14]**
Total events	28		0			
Heterogeneity: Chi² = 4.30, df = 4 (P = 0.37); I² = 7%						
Test for overall effect: Z = 3.37 (P = 0.0008)						
Test for subgroup differences: Not applicable						

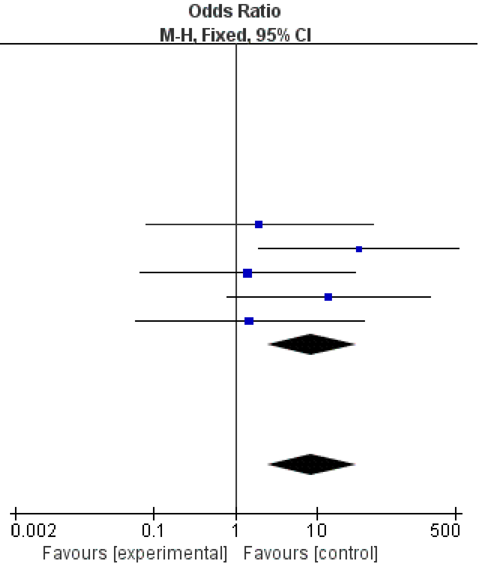

(C)

Study or Subgroup	Experimental Events	Experimental Total	Control Events	Control Total	Weight	Odds Ratio M-H, Fixed, 95% CI
**6.4.1 AJ**						
**Subtotal (95% CI)**		**0**		**0**		**Not estimable**
Total events	0		0			
Heterogeneity: Not applicable						
Test for overall effect: Not applicable						
**6.4.2 NON-AJ**						
2009Juliane Neumann	1	790	0	257	25.6%	0.98 [0.04, 24.09]
2009Jun Mitsui	1	534	0	544	16.8%	3.06 [0.12, 75.33]
2011S. Lesage(2)	2	194	0	177	17.6%	4.61 [0.22, 96.69]
2011Suzanne Lesage(1)	1	1391	0	391	26.6%	0.84 [0.03, 20.78]
2013Raquel Duran	1	185	0	283	13.4%	4.61 [0.19, 113.77]
**Subtotal (95% CI)**		**3094**		**1652**	**100.0%**	**2.42 [0.59, 9.84]**
Total events	6		0			
Heterogeneity: Chi² = 1.07, df = 4 (P = 0.90); I² = 0%						
Test for overall effect: Z = 1.23 (P = 0.22)						
**Total (95% CI)**		**3094**		**1652**	**100.0%**	**2.42 [0.59, 9.84]**
Total events	6		0			
Heterogeneity: Chi² = 1.07, df = 4 (P = 0.90); I² = 0%						
Test for overall effect: Z = 1.23 (P = 0.22)						
Test for subgroup differences: Not applicable						

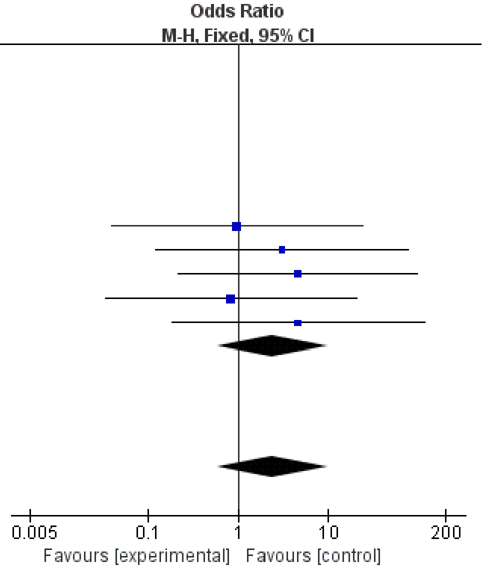

(D)

Study or Subgroup	Experimental Events	Experimental Total	Control Events	Control Total	Weight	Odds Ratio M-H, Fixed, 95% CI
**6.5.1 AJ**						
**Subtotal (95% CI)**		**0**		**0**		**Not estimable**
Total events	0		0			
Heterogeneity: Not applicable						
Test for overall effect: Not applicable						
**6.5.2 NON-AJ**						
2008Usha Gutt	1	184	0	92	7.7%	1.51 [0.06, 37.49]
2009Jun Mitsui	4	534	7	544	80.0%	0.58 [0.17, 1.99]
2012Jung Mi Choi	1	277	0	299	5.6%	3.25 [0.13, 80.10]
2014Zhe Yu	1	184	0	130	6.8%	2.13 [0.09, 52.79]
**Subtotal (95% CI)**		**1179**		**1065**	**100.0%**	**0.90 [0.34, 2.38]**
Total events	7		7			
Heterogeneity: Chi² = 1.49, df = 3 (P = 0.69); I² = 0%						
Test for overall effect: Z = 0.20 (P = 0.84)						
**Total (95% CI)**		**1179**		**1065**	**100.0%**	**0.90 [0.34, 2.38]**
Total events	7		7			
Heterogeneity: Chi² = 1.49, df = 3 (P = 0.69); I² = 0%						
Test for overall effect: Z = 0.20 (P = 0.84)						
Test for subgroup differences: Not applicable						

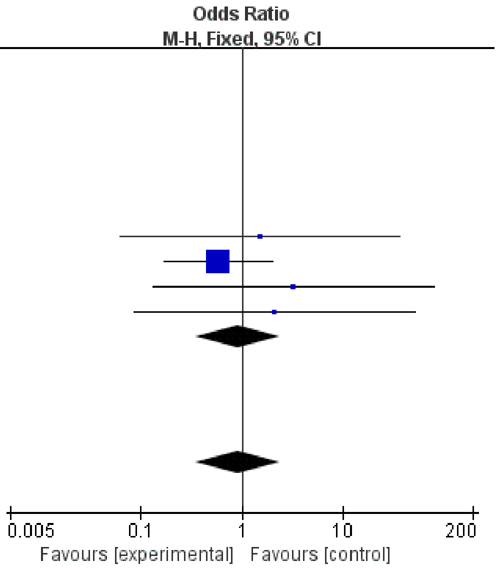

(E)

Study or Subgroup	Experimental Events	Experimental Total	Control Events	Control Total	Weight	Odds Ratio M-H, Fixed, 95% CI
**6.7.1 AJ**						
**Subtotal (95% CI)**		**0**		**0**		**Not estimable**
Total events	0		0			
Heterogeneity: Not applicable						
Test for overall effect: Not applicable						
**6.7.2 NON-AJ**						
2009 Kallirhoe Kalinderi	4	172	0	132	14.1%	7.08 [0.38, 132.62]
2011Marina Moraitou	7	205	1	206	24.7%	7.25 [0.88, 59.44]
2012K. R. Kumar	6	360	2	348	51.2%	2.93 [0.59, 14.63]
2013Raquel Duran	1	185	0	283	10.0%	4.61 [0.19, 113.77]
**Subtotal (95% CI)**		**922**		**969**	**100.0%**	**4.75 [1.60, 14.09]**
Total events	18		3			
Heterogeneity: Chi² = 0.57, df = 3 (P = 0.90); I² = 0%						
Test for overall effect: Z = 2.81 (P = 0.005)						
**Total (95% CI)**		**922**		**969**	**100.0%**	**4.75 [1.60, 14.09]**
Total events	18		3			
Heterogeneity: Chi² = 0.57, df = 3 (P = 0.90); I² = 0%						
Test for overall effect: Z = 2.81 (P = 0.005)						
Test for subgroup differences: Not applicable						

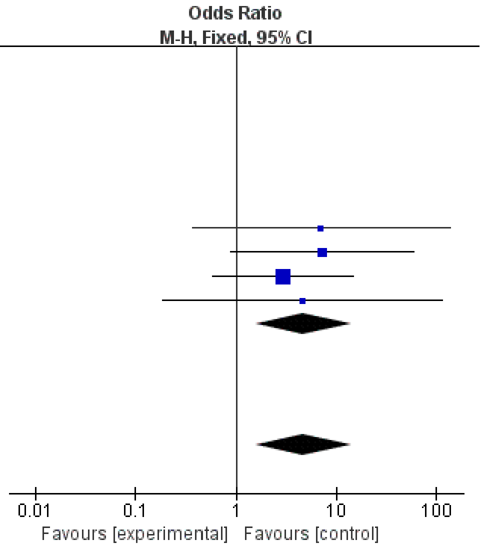

(F)

(G)

Study or Subgroup	Experimental Events	Experimental Total	Control Events	Control Total	Weight	Odds Ratio M-H, Fixed, 95% CI
**6.8.1 AJ**						
2007L.N. Clark	1	178	1	85	1.2%	0.47 [0.03, 7.68]
**Subtotal (95% CI)**		**178**		**85**	**1.2%**	**0.47 [0.03, 7.68]**
Total events	1		1			
Heterogeneity: Not applicable						
Test for overall effect: Z = 0.52 (P = 0.60)						
**6.8.2 NON-AJ**						
2007Jose Bras	2	230	3	430	1.8%	1.25 [0.21, 7.53]
2007Mariana Spitz	1	65	0	267	0.2%	12.44 [0.50, 308.96]
2007Shira G. Ziegler	18	92	0	92	0.4%	45.94 [2.72, 775.00]
2008W.C. Nichols	28	450	11	359	10.2%	2.10 [1.03, 4.28]
2009 Kallirhoe Kalinderi	1	172	1	132	1.0%	0.77 [0.05, 12.36]
2011Suzanne Lesage(1)	49	1391	8	391	10.7%	1.75 [0.82, 3.72]
2013Raquel Duran	14	185	0	283	0.3%	47.94 [2.84, 808.75]
2014Zhe Yu	1	184	0	130	0.5%	2.13 [0.09, 52.79]
2015Fabin Han	4	225	4	110	4.7%	0.48 [0.12, 1.96]
2016Caroline Ran	90	1625	65	2025	48.4%	1.77 [1.28, 2.45]
2016David Crosiers	12	266	15	536	8.4%	1.64 [0.76, 3.56]
2016Silvia Jesús	16	532	13	542	11.1%	1.26 [0.60, 2.65]
2017Melinda Barkhuizen	5	105	1	40	1.2%	1.95 [0.22, 17.23]
**Subtotal (95% CI)**		**5522**		**5337**	**98.8%**	**1.98 [1.58, 2.49]**
Total events	241		121			
Heterogeneity: Chi² = 17.79, df = 12 (P = 0.12); I² = 33%						
Test for overall effect: Z = 5.94 (P < 0.00001)						
**Total (95% CI)**		**5700**		**5422**	**100.0%**	**1.97 [1.57, 2.46]**
Total events	242		122			
Heterogeneity: Chi² = 18.63, df = 13 (P = 0.13); I² = 30%						
Test for overall effect: Z = 5.88 (P < 0.00001)						
Test for subgroup differences: Chi² = 1.01, df = 1 (P = 0.32), I² = 0.7%						

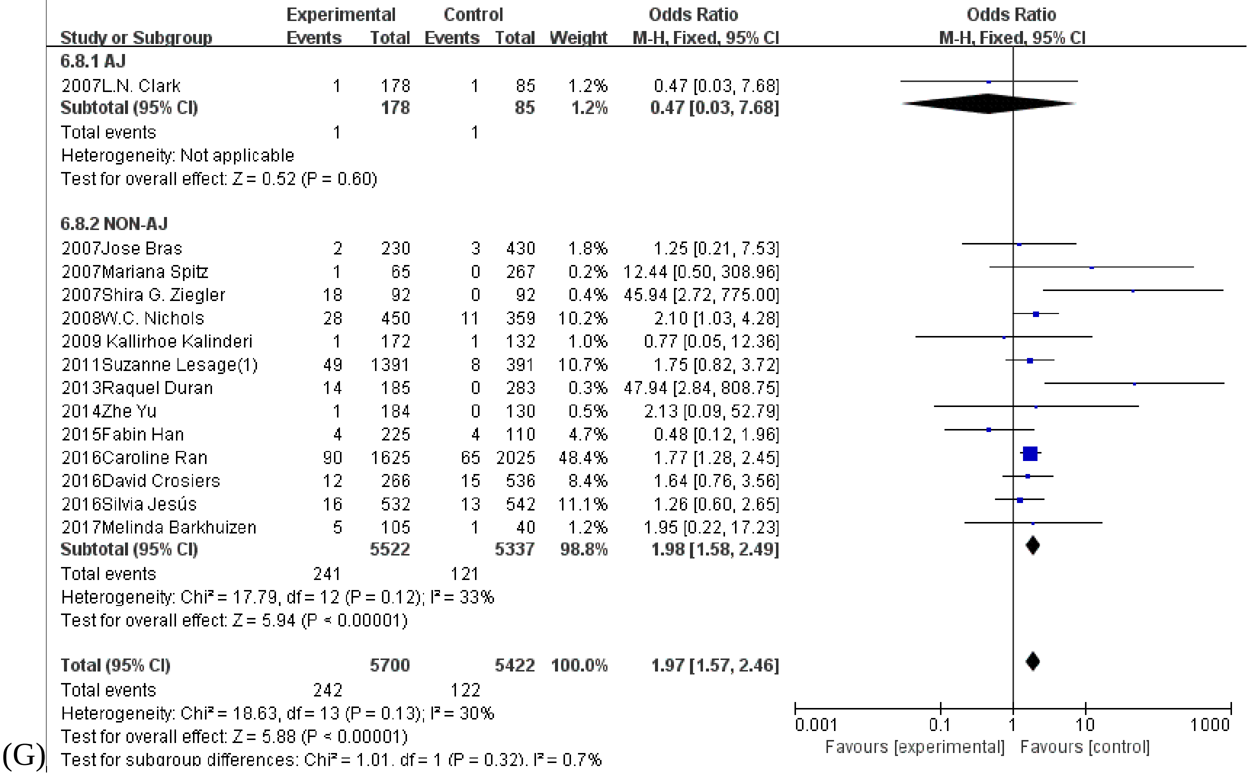

(H)

Study or Subgroup	Experimental Events	Experimental Total	Control Events	Control Total	Weight	Odds Ratio M-H, Fixed, 95% CI
**6.9.1 AJ**						
2007L.N. Clark	1	178	2	85	6.9%	0.23 [0.02, 2.62]
**Subtotal (95% CI)**		**178**		**85**	**6.9%**	**0.23 [0.02, 2.62]**
Total events	1		2			
Heterogeneity: Not applicable						
Test for overall effect: Z = 1.18 (P = 0.24)						
**6.9.2 NON-AJ**						
2007Jose Bras	2	230	5	430	8.9%	0.75 [0.14, 3.87]
2007L.N. Clark	2	100	1	94	2.6%	1.90 [0.17, 21.28]
2007Shira G. Ziegler	18	92	0	92	1.0%	45.94 [2.72, 775.00]
2008W.C. Nichols	10	450	4	359	11.2%	2.02 [0.63, 6.49]
2011Marina Moraitou	0	205	1	206	3.8%	0.33 [0.01, 8.23]
2011Nu´ ria Seto´ -Salvia	2	225	0	186	1.4%	4.17 [0.20, 87.45]
2011S. Lesage(2)	2	194	1	177	2.7%	1.83 [0.16, 20.39]
2011Suzanne Lesage(1)	17	1391	1	391	4.0%	4.83 [0.64, 36.37]
2012K. R. Kumar	7	360	6	348	15.3%	1.13 [0.38, 3.40]
2013Raquel Duran	1	185	1	283	2.0%	1.53 [0.10, 24.66]
2015Fabin Han	11	225	4	110	13.1%	1.36 [0.42, 4.38]
2016David Crosiers	3	266	11	536	18.5%	0.54 [0.15, 1.97]
2016Silvia Jesús	5	532	2	542	5.0%	2.56 [0.49, 13.26]
2017Melinda Barkhuizen	2	105	1	40	3.6%	0.76 [0.07, 8.59]
**Subtotal (95% CI)**		**4560**		**3794**	**93.1%**	**1.89 [1.27, 2.83]**
Total events	82		38			
Heterogeneity: Chi² = 13.80, df = 13 (P = 0.39); I² = 6%						
Test for overall effect: Z = 3.10 (P = 0.002)						
**Total (95% CI)**		**4738**		**3879**	**100.0%**	**1.78 [1.20, 2.64]**
Total events	83		40			
Heterogeneity: Chi² = 15.98, df = 14 (P = 0.31); I² = 12%						
Test for overall effect: Z = 2.86 (P = 0.004)						
Test for subgroup differences: Chi² = 2.80, df = 1 (P = 0.09), I² = 64.2%						

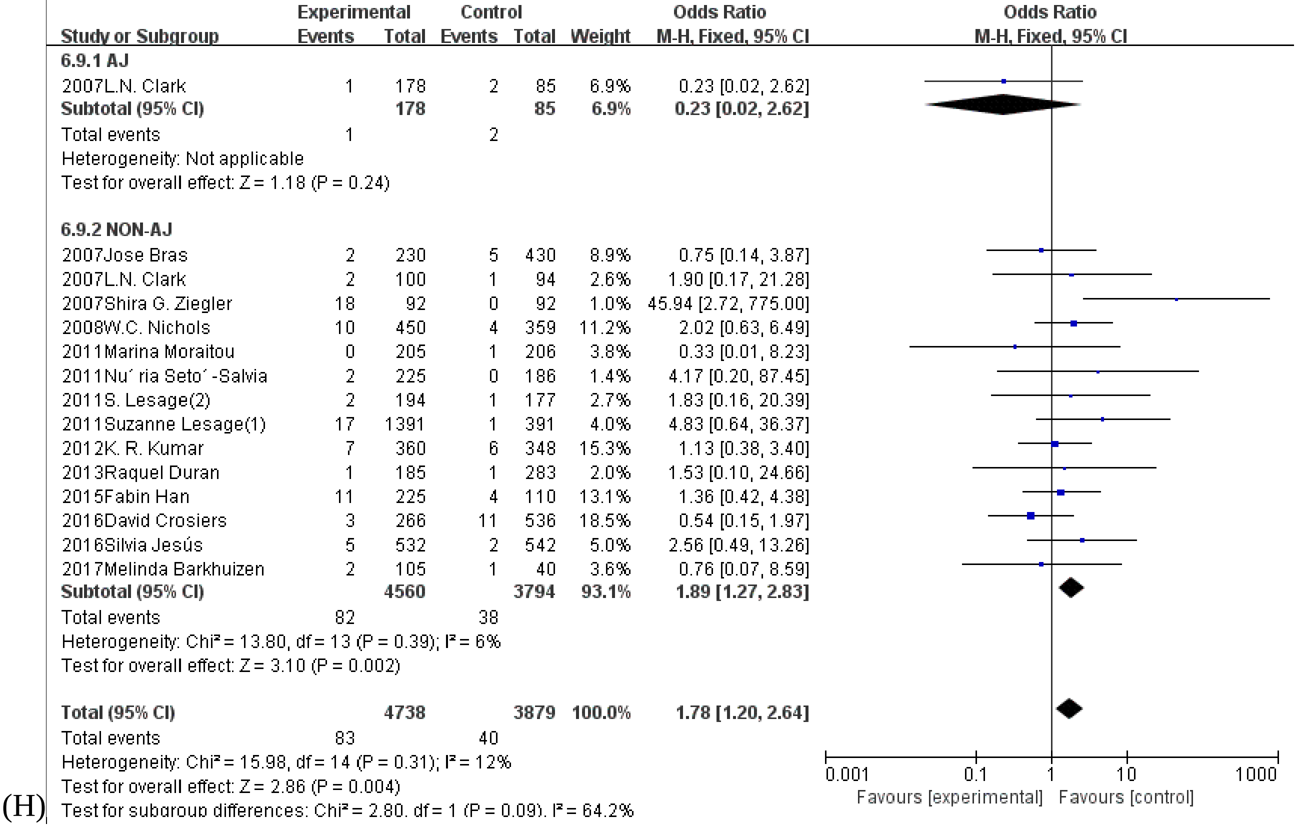

Study or Subgroup	Experimental Events	Experimental Total	Control Events	Control Total	Weight	Odds Ratio M-H, Fixed, 95% CI
**6.12.1 AJ**						
2004Judith Aharon-Peretz	26	99	92	1543	7.7%	5.62 [3.43, 9.21]
2005Lorraine N. Clark	17	160	4	92	4.3%	2.62 [0.85, 8.02]
2007L.N. Clark	21	178	3	85	3.4%	3.66 [1.06, 12.62]
2008Z. Gan-Or	54	420	237	4138	35.8%	2.43 [1.77, 3.33]
2015E. Dagan	54	287	14	400	8.9%	6.39 [3.47, 11.76]
**Subtotal (95% CI)**		**1144**		**6258**	**60.1%**	**3.51 [2.78, 4.43]**
Total events	172		350			
Heterogeneity: Chi² = 12.72, df = 4 (P = 0.01); I² = 69%						
Test for overall effect: Z = 10.58 (P < 0.00001)						
**6.12.2 NON-AJ**						
2005Christine Sato	1	88	1	122	0.8%	1.39 [0.09, 22.54]
2006M. Toft	4	311	7	474	5.2%	0.87 [0.25, 2.99]
2007Elvira V. De Marco	3	395	0	483	0.4%	8.62 [0.44, 167.44]
2007Jose Bras	5	230	3	430	1.9%	3.16 [0.75, 13.36]
2007L.N. Clark	2	100	0	94	0.5%	4.80 [0.23, 101.23]
2008Ignacio F. Mata	11	721	2	554	2.1%	4.28 [0.94, 19.37]
2008W.C. Nichols	6	450	3	359	3.1%	1.60 [0.40, 6.46]
2009Juliane Neumann	8	790	1	257	1.4%	2.62 [0.33, 21.04]
2010Adriana Vaz dos Santos	2	110	0	155	0.4%	7.17 [0.34, 150.74]
2010F.-Y. Hu	6	328	2	300	1.9%	2.78 [0.56, 13.86]
2010Kenya Nishioka	1	428	3	372	3.0%	0.29 [0.03, 2.78]
2011Marina Moraitou	6	205	4	206	3.6%	1.52 [0.42, 5.48]
2011Nu´ ria Seto´-Salvia	5	225	0	186	0.5%	9.30 [0.51, 169.36]
2011S. Lesage(2)	2	194	0	177	0.5%	4.61 [0.22, 96.69]
2011Suzanne Lesage(1)	45	1391	2	391	2.8%	6.50 [1.57, 26.93]
2012Anton Emelyanov	2	330	0	240	0.5%	3.66 [0.17, 76.60]
2012Beatriz de Carvalho Guimarães	3	237	0	186	0.5%	5.57 [0.29, 108.45]
2012K. R. Kumar	9	360	0	348	0.5%	18.84 [1.09, 324.91]
2013Raquel Duran	5	185	1	283	0.7%	7.83 [0.91, 67.60]
2014Rosanna Asselta	69	2350	4	1111	5.0%	8.37 [3.05, 23.00]
2015Fabin Han	2	225	0	110	0.6%	2.47 [0.12, 51.93]
2016Caroline Ran	10	1625	2	2025	1.7%	6.26 [1.37, 28.62]
2016David Crosiers	3	266	1	536	0.6%	6.10 [0.63, 58.95]
2016Silvia Jesús	5	532	0	542	0.5%	11.31 [0.62, 205.09]
2017Melinda Barkhuizen	1	105	0	40	0.7%	1.16 [0.05, 29.13]
2017Thomas R. Barber	1	106	1	283	0.5%	2.69 [0.17, 43.33]
**Subtotal (95% CI)**		**12287**		**10264**	**39.9%**	**3.98 [2.82, 5.63]**
Total events	217		37			
Heterogeneity: Chi² = 22.36, df = 25 (P = 0.62); I² = 0%						
Test for overall effect: Z = 7.83 (P < 0.00001)						
**Total (95% CI)**		**13431**		**16522**	**100.0%**	**3.70 [3.04, 4.50]**
Total events	389		387			
Heterogeneity: Chi² = 34.89, df = 30 (P = 0.25); I² = 14%						
Test for overall effect: Z = 13.04 (P < 0.00001)						
Test for subgroup differences: Chi² = 0.36, df = 1 (P = 0.55), I² = 0%						

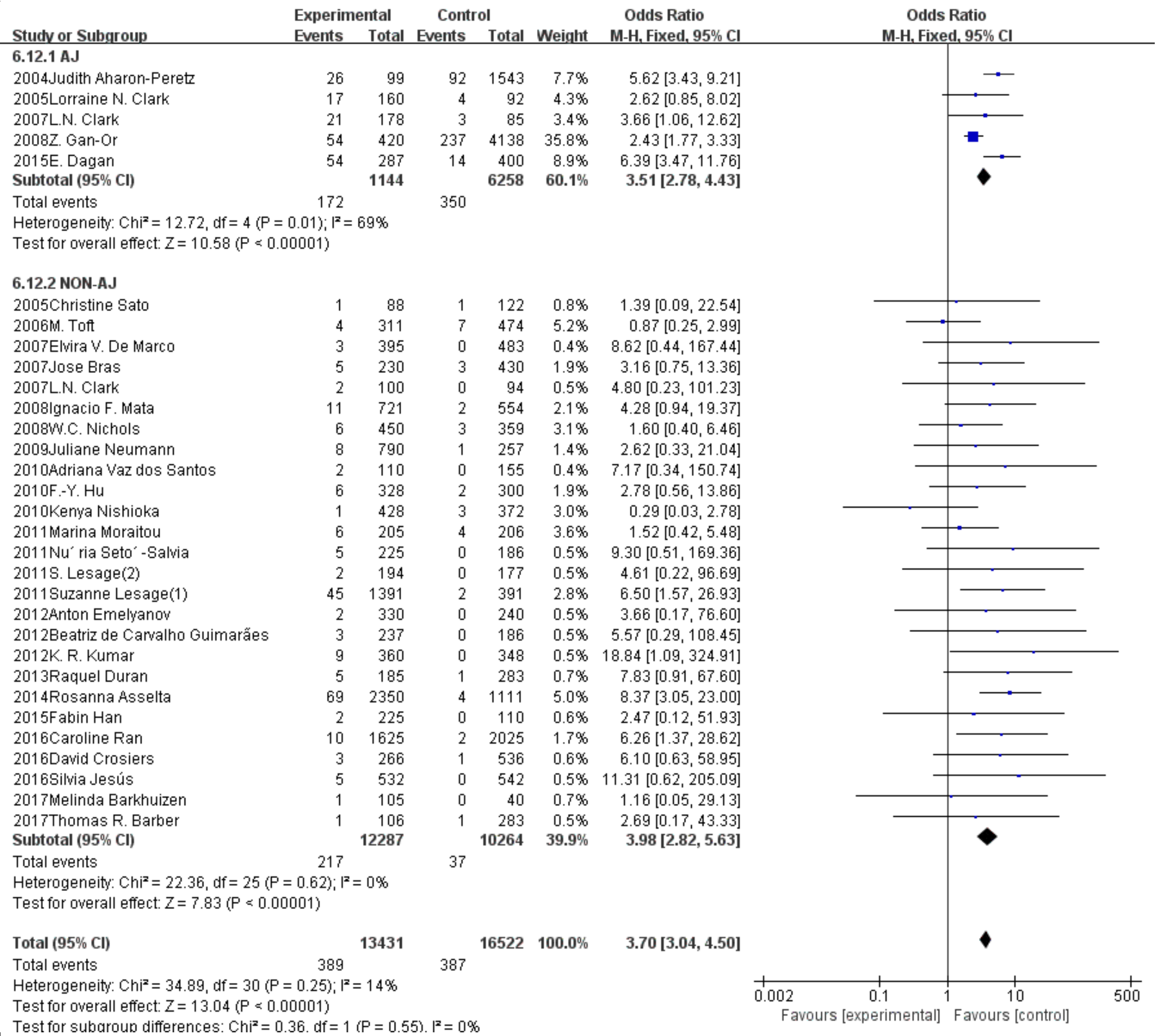

(I)

Study or Subgroup	Experimental Events	Experimental Total	Control Events	Control Total	Weight	Odds Ratio M-H, Fixed, 95% CI
**6.10.1 AJ**						
**Subtotal (95% CI)**		**0**		**0**		**Not estimable**
Total events	0		0			
Heterogeneity: Not applicable						
Test for overall effect: Not applicable						
**6.10.2 NON-AJ**						
2007Jose Bras	0	230	2	430	25.7%	0.37 [0.02, 7.78]
2011Suzanne Lesage(1)	1	1391	1	391	23.0%	0.28 [0.02, 4.50]
2012K. R. Kumar	0	360	1	348	22.4%	0.32 [0.01, 7.91]
2013Michael A. Nalls	0	151	1	1962	3.2%	4.32 [0.18, 106.39]
2013Raquel Duran	1	185	0	283	5.8%	4.61 [0.19, 113.77]
2014Rosanna Asselta	5	2350	1	1111	20.0%	2.37 [0.28, 20.28]
**Subtotal (95% CI)**		**4667**		**4525**	**100.0%**	**1.11 [0.39, 3.13]**
Total events	7		6			
Heterogeneity: Chi² = 3.94, df = 5 (P = 0.56); I² = 0%						
Test for overall effect: Z = 0.19 (P = 0.85)						
**Total (95% CI)**		**4667**		**4525**	**100.0%**	**1.11 [0.39, 3.13]**
Total events	7		6			
Heterogeneity: Chi² = 3.94, df = 5 (P = 0.56); I² = 0%						
Test for overall effect: Z = 0.19 (P = 0.85)						
Test for subgroup differences: Not applicable						

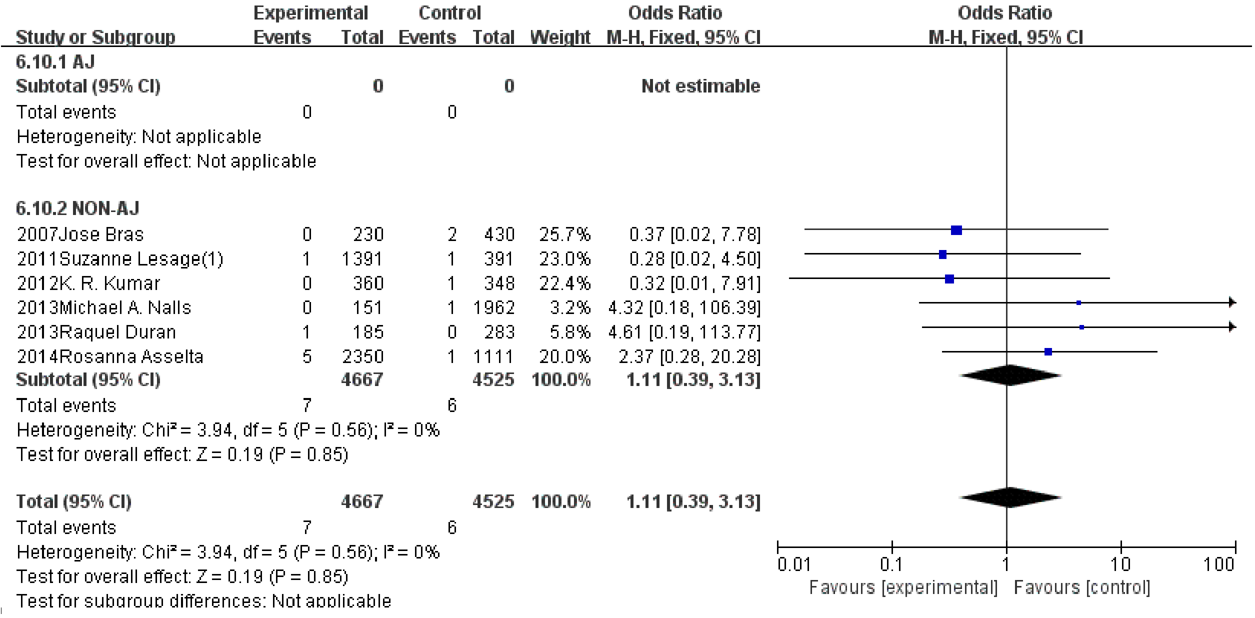

(J)

(K)

Study or Subgroup	Experimental Events	Experimental Total	Control Events	Control Total	Weight	Odds Ratio M-H, Fixed, 95% CI
**6.13.1 AJ**						
2008Z. Gan-Or	2	420	0	4138	1.0%	49.44 [2.37, 1031.61]
**Subtotal (95% CI)**		**420**		**4138**	**1.0%**	**49.44 [2.37, 1031.61]**
Total events	2		0			
Heterogeneity: Not applicable						
Test for overall effect: Z = 2.52 (P = 0.01)						
**6.13.2 NON-AJ**						
2007Jose Bras	1	230	0	430	3.7%	5.63 [0.23, 138.69]
2007L.N. Clark	1	100	0	94	5.4%	2.85 [0.11, 70.81]
2007Shira G. Ziegler	1	92	0	92	5.2%	3.03 [0.12, 75.42]
2008Usha Gutt	1	184	1	92	14.1%	0.50 [0.03, 8.04]
2009 Kallirhoe Kalinderi	1	172	0	132	6.0%	2.32 [0.09, 57.35]
2009Juliane Neumann	1	790	0	257	8.0%	0.98 [0.04, 24.09]
2011C.-L. Huang	2	967	0	780	5.9%	4.04 [0.19, 84.31]
2011Marina Moraitou	7	205	0	206	5.1%	15.60 [0.89, 275.03]
2011Nu´ ria Seto´ -Salvia	2	225	0	186	5.8%	4.17 [0.20, 87.45]
2011Suzanne Lesage(1)	1	1391	0	391	8.3%	0.84 [0.03, 20.78]
2012K. R. Kumar	6	360	2	348	21.3%	2.93 [0.59, 14.63]
2013Raquel Duran	1	185	0	283	4.2%	4.61 [0.19, 113.77]
2014Yuanzhe Li	4	147	0	100	6.1%	6.30 [0.34, 118.38]
**Subtotal (95% CI)**		**5048**		**3391**	**99.0%**	**3.39 [1.60, 7.16]**
Total events	29		3			
Heterogeneity: Chi² = 4.65, df = 12 (P = 0.97); I² = 0%						
Test for overall effect: Z = 3.20 (P = 0.001)						
**Total (95% CI)**		**5468**		**7529**	**100.0%**	**3.84 [1.86, 7.91]**
Total events	31		3			
Heterogeneity: Chi² = 7.70, df = 13 (P = 0.86); I² = 0%						
Test for overall effect: Z = 3.64 (P = 0.0003)						
Test for subgroup differences: Chi² = 2.82, df = 1 (P = 0.09), I² = 64.5%						

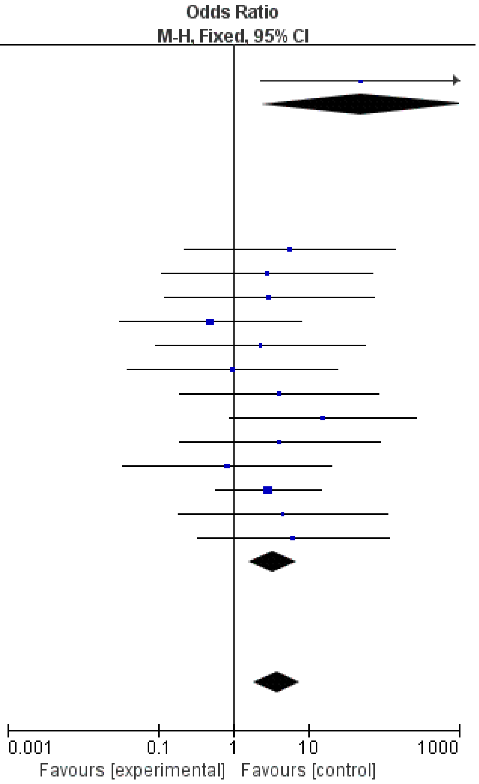

(L)

Study or Subgroup	Experimental Events	Experimental Total	Control Events	Control Total	Weight	Odds Ratio M-H, Fixed, 95% CI
**6.14.1 AJ**						
**Subtotal (95% CI)**		**0**		**0**		**Not estimable**
Total events	0		0			
Heterogeneity: Not applicable						
Test for overall effect: Not applicable						
**6.14.2 NON-AJ**						
2009Juliane Neumann	1	790	0	257	22.4%	0.98 [0.04, 24.09]
2011S. Lesage(2)	0	194	1	177	46.6%	0.30 [0.01, 7.47]
2013Michael A. Nalls	0	151	2	1962	10.7%	2.59 [0.12, 54.15]
2014Rosanna Asselta	1	2350	0	1111	20.2%	1.42 [0.06, 34.87]
**Subtotal (95% CI)**		**3485**		**3507**	**100.0%**	**0.92 [0.20, 4.32]**
Total events	2		3			
Heterogeneity: Chi² = 0.98, df = 3 (P = 0.81); I² = 0%						
Test for overall effect: Z = 0.10 (P = 0.92)						
**Total (95% CI)**		**3485**		**3507**	**100.0%**	**0.92 [0.20, 4.32]**
Total events	2		3			
Heterogeneity: Chi² = 0.98, df = 3 (P = 0.81); I² = 0%						
Test for overall effect: Z = 0.10 (P = 0.92)						
Test for subgroup differences: Not applicable						

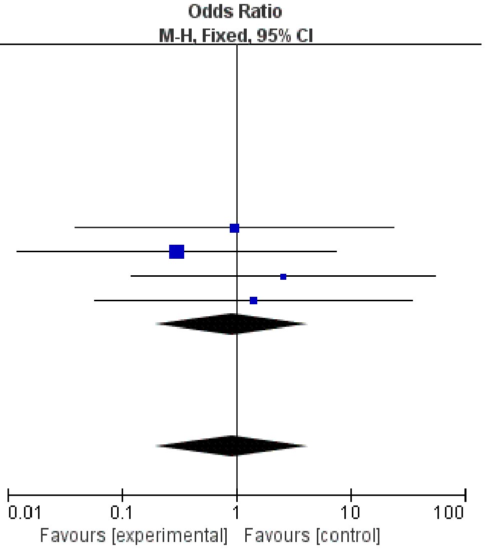

Study or Subgroup	Experimental Events	Experimental Total	Control Events	Control Total	Weight	Odds Ratio M-H, Fixed, 95% CI
**6.15.1 AJ**						
2008Z. Gan-Or	2	420	4	4138	1.8%	4.94 [0.90, 27.08]
**Subtotal (95% CI)**		**420**		**4138**	**1.8%**	**4.94 [0.90, 27.08]**
Total events	2		4			
Heterogeneity: Not applicable						
Test for overall effect: Z = 1.84 (P = 0.07)						
**6.15.2 NON-AJ**						
2005Christine Sato	1	88	0	122	1.0%	4.20 [0.17, 104.32]
2006M. Toft	3	311	1	474	1.9%	4.61 [0.48, 44.49]
2007Elvira V. De Marco	8	395	1	483	2.1%	9.96 [1.24, 80.01]
2007Eng-King Tan	8	331	0	347	1.2%	18.26 [1.05, 317.65]
2007Jose Bras	3	230	0	430	0.8%	13.25 [0.68, 257.56]
2007L.N. Clark	2	100	0	94	1.2%	4.80 [0.23, 101.23]
2007Mariana Spitz	2	65	0	267	0.5%	21.06 [1.00, 444.16]
2007Shira G. Ziegler	1	92	0	92	1.2%	3.03 [0.12, 75.42]
2007Yih-Ru Wu	13	518	2	339	5.7%	4.34 [0.97, 19.34]
2008Ignacio F. Mata	10	721	1	554	2.7%	7.78 [0.99, 60.94]
2008Usha Gutt	4	184	1	92	3.2%	2.02 [0.22, 18.36]
2008W.C. Nichols	9	450	0	359	1.3%	15.47 [0.90, 266.72]
2009 Kallirhoe Kalinderi	2	172	0	132	1.4%	3.89 [0.18, 81.62]
2009Juliane Neumann	11	790	0	257	1.8%	7.60 [0.45, 129.38]
2009Jun Mitsui	8	534	0	544	1.2%	17.58 [1.01, 305.37]
2010Adriana Vaz dos Santos	2	110	0	155	1.0%	7.17 [0.34, 150.74]
2010Qi-Ying Sun	11	402	0	413	1.2%	24.29 [1.43, 413.63]
2010X.-Y. Mao	20	616	1	411	2.8%	13.76 [1.84, 102.92]
2011C.-L. Huang	27	967	1	780	2.6%	22.38 [3.03, 165.03]
2011Marina Moraitou	6	205	1	206	2.3%	6.18 [0.74, 51.80]
2011Nu´ ria Seto´ -Salvia	6	225	0	186	1.3%	11.05 [0.62, 197.37]
2011Suzanne Lesage(1)	19	1391	0	391	1.9%	11.12 [0.67, 184.66]
2012Anton Emelyanov	6	330	2	240	5.5%	2.20 [0.44, 11.01]
2012Beatriz de Carvalho Guimarães	6	237	0	186	1.3%	10.47 [0.59, 187.11]
2012Jung Mi Choi	2	277	0	291	1.2%	5.29 [0.25, 110.69]
2012K. R. Kumar	2	360	1	348	2.5%	1.94 [0.17, 21.48]
2012Xiong Zhang	6	195	0	443	0.7%	30.42 [1.71, 542.79]
2012Youpei Wang	8	208	1	298	1.9%	11.88 [1.47, 95.72]
2013M de L Gonz´ alez-del Rinc´ on	7	128	0	252	0.8%	31.17 [1.77, 550.28]
2013Michael A. Nalls	3	151	3	1962	1.0%	13.24 [2.65, 66.15]
2013Raquel Duran	2	185	0	283	0.9%	7.72 [0.37, 161.82]
2014Rosanna Asselta	47	2350	3	1111	9.7%	7.54 [2.34, 24.27]
2014Teeratorn Pulkes	15	480	1	395	2.6%	12.71 [1.67, 96.65]
2014Yuanzhe Li	12	147	0	100	1.3%	18.54 [1.09, 316.88]
2014Zhe Yu	2	184	0	130	1.4%	3.58 [0.17, 75.09]
2015Fabin Han	4	225	0	110	1.6%	4.49 [0.24, 84.14]
2015Guo ji-feng	26	1019	1	1030	2.4%	26.94 [3.65, 198.92]
2016Caroline Ran	35	1625	3	2025	6.3%	14.84 [4.55, 48.33]
2016David Crosiers	3	266	1	536	1.6%	6.10 [0.63, 58.95]
2016Rita Török	3	124	0	122	1.2%	7.06 [0.36, 138.09]
2016Silvia Jesús	13	532	6	542	14.1%	2.24 [0.84, 5.93]
**Subtotal (95% CI)**		**17920**		**17532**	**98.2%**	**8.72 [6.27, 12.11]**
Total events	378		31			
Heterogeneity: Chi² = 23.50, df = 40 (P = 0.98); I² = 0%						
Test for overall effect: Z = 12.91 (P < 0.00001)						
**Total (95% CI)**		**18340**		**21670**	**100.0%**	**8.65 [6.25, 11.96]**
Total events	380		35			
Heterogeneity: Chi² = 23.83, df = 41 (P = 0.99); I² = 0%						
Test for overall effect: Z = 13.04 (P < 0.00001)						
Test for subgroup differences: Chi² = 0.41, df = 1 (P = 0.52), I² = 0%						

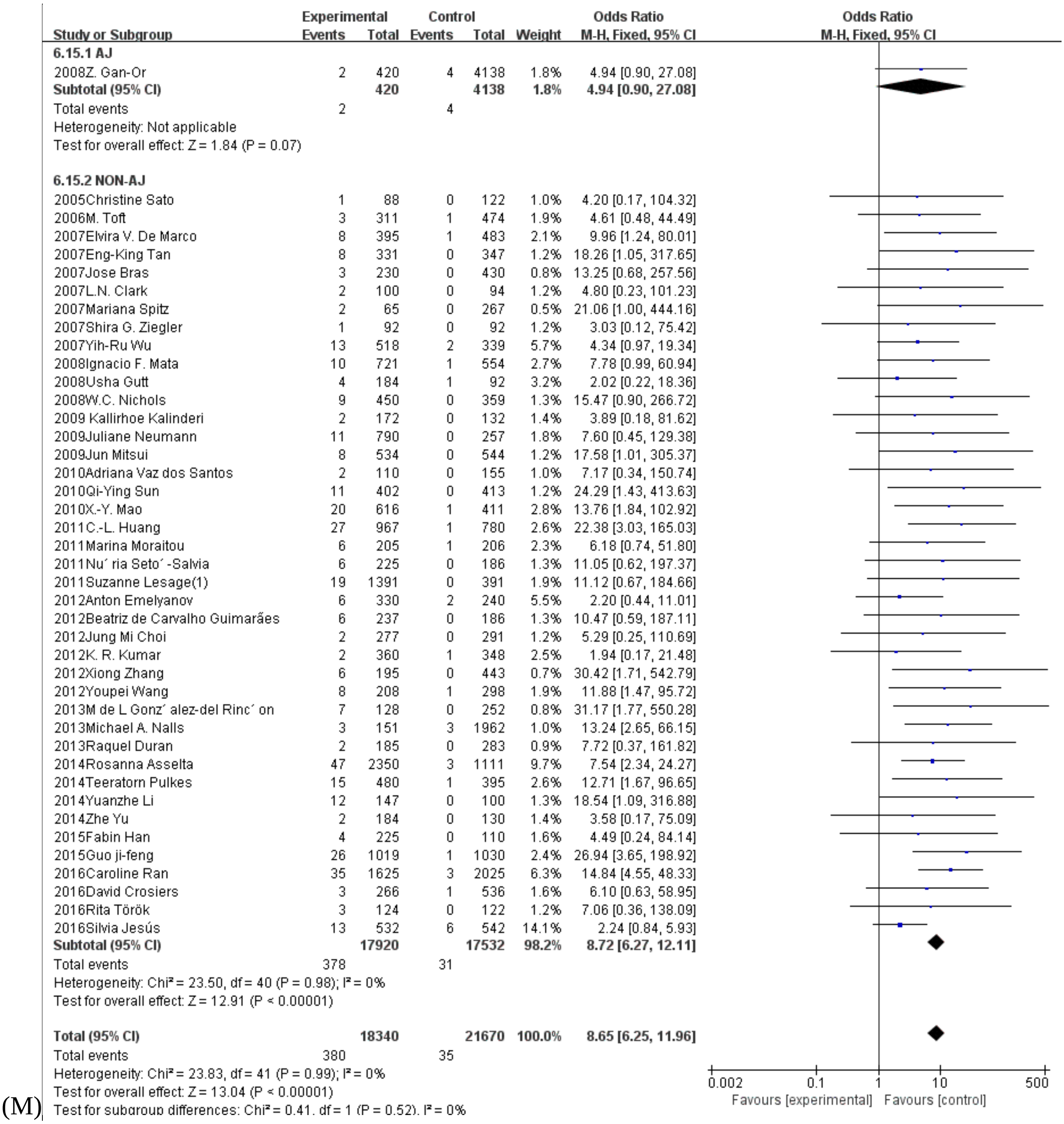

(M)

Study or Subgroup	Experimental Events	Experimental Total	Control Events	Control Total	Weight	Odds Ratio M-H, Fixed, 95% CI
**6.16.1 AJ**						
**Subtotal (95% CI)**		**0**		**0**		**Not estimable**
Total events	0		0			
Heterogeneity: Not applicable						
Test for overall effect: Not applicable						
**6.16.2 NON-AJ**						
2007Shira G. Ziegler	0	92	1	92	17.4%	0.33 [0.01, 8.20]
2008Usha Gutt	0	184	1	92	23.2%	0.17 [0.01, 4.10]
2009 Kallirhoe Kalinderi	0	172	2	132	32.8%	0.15 [0.01, 3.18]
2009Jun Mitsui	2	534	1	544	11.5%	2.04 [0.18, 22.58]
2011S. Lesage(2)	1	194	0	177	6.0%	2.75 [0.11, 67.99]
2011Suzanne Lesage(1)	1	1391	0	391	9.1%	0.84 [0.03, 20.78]
**Subtotal (95% CI)**		**2567**		**1428**	**100.0%**	**0.62 [0.22, 1.80]**
Total events	4		5			
Heterogeneity: Chi² = 3.43, df = 5 (P = 0.63); I² = 0%						
Test for overall effect: Z = 0.87 (P = 0.38)						
**Total (95% CI)**		**2567**		**1428**	**100.0%**	**0.62 [0.22, 1.80]**
Total events	4		5			
Heterogeneity: Chi² = 3.43, df = 5 (P = 0.63); I² = 0%						
Test for overall effect: Z = 0.87 (P = 0.38)						
Test for subgroup differences: Not applicable						

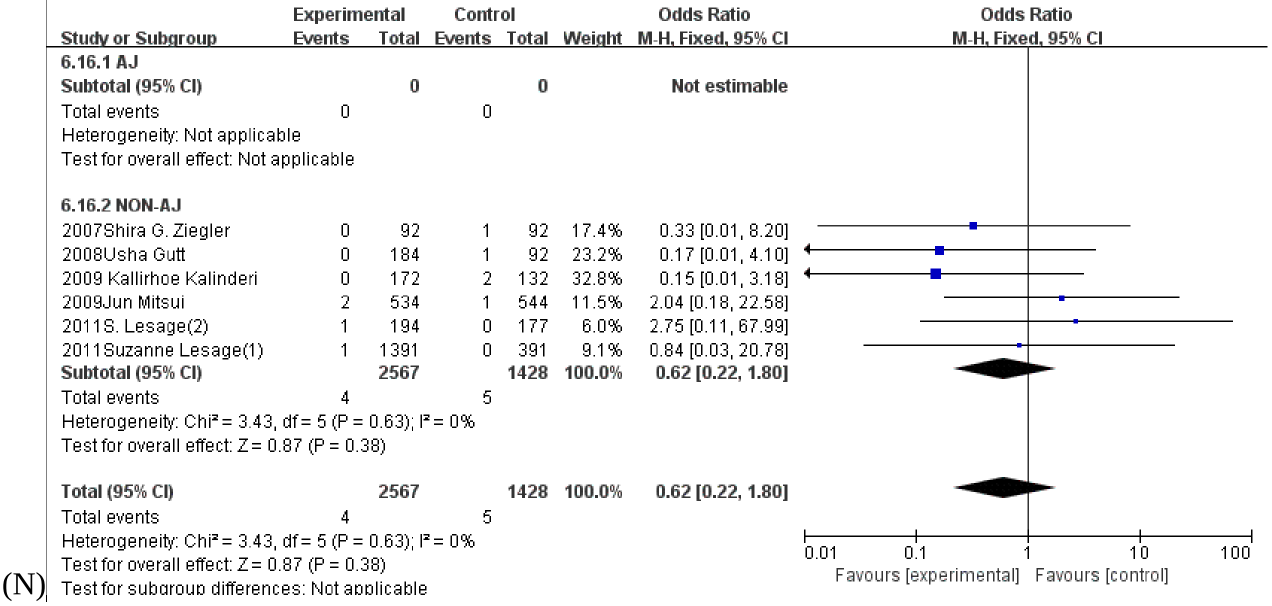

(N)

Study or Subgroup	Experimental Events	Experimental Total	Control Events	Control Total	Weight	Odds Ratio M-H, Fixed, 95% CI
**6.17.1 AJ**						
**Subtotal (95% CI)**		**0**		**0**		**Not estimable**
Total events	0		0			
Heterogeneity: Not applicable						
Test for overall effect: Not applicable						
**6.17.2 NON-AJ**						
2009Juliane Neumann	3	790	0	257	28.4%	2.29 [0.12, 44.46]
2011Suzanne Lesage(1)	2	1391	0	391	29.5%	1.41 [0.07, 29.40]
2012K. R. Kumar	1	360	0	348	19.2%	2.91 [0.12, 71.63]
2013Michael A. Nalls	0	151	1	1962	8.1%	4.32 [0.18, 106.39]
2013Raquel Duran	3	185	0	283	14.7%	10.87 [0.56, 211.75]
**Subtotal (95% CI)**		**2877**		**3241**	**100.0%**	**3.58 [0.88, 14.50]**
Total events	9		1			
Heterogeneity: Chi² = 1.02, df = 4 (P = 0.91); I² = 0%						
Test for overall effect: Z = 1.78 (P = 0.07)						
**Total (95% CI)**		**2877**		**3241**	**100.0%**	**3.58 [0.88, 14.50]**
Total events	9		1			
Heterogeneity: Chi² = 1.02, df = 4 (P = 0.91); I² = 0%						
Test for overall effect: Z = 1.78 (P = 0.07)						
Test for subgroup differences: Not applicable						

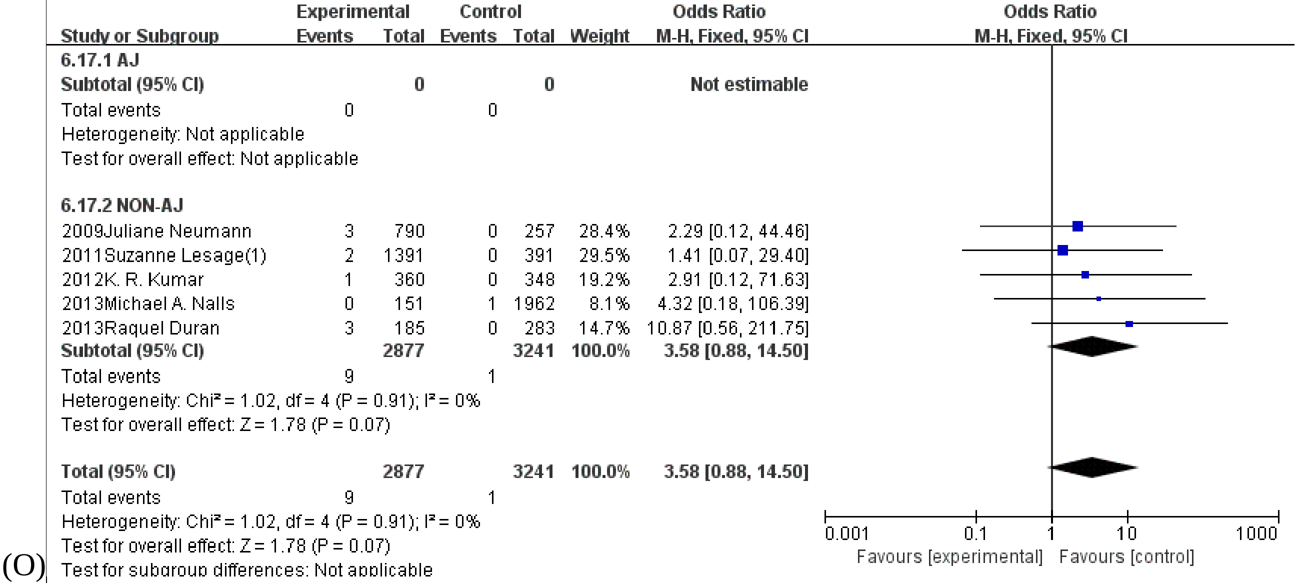

(O)

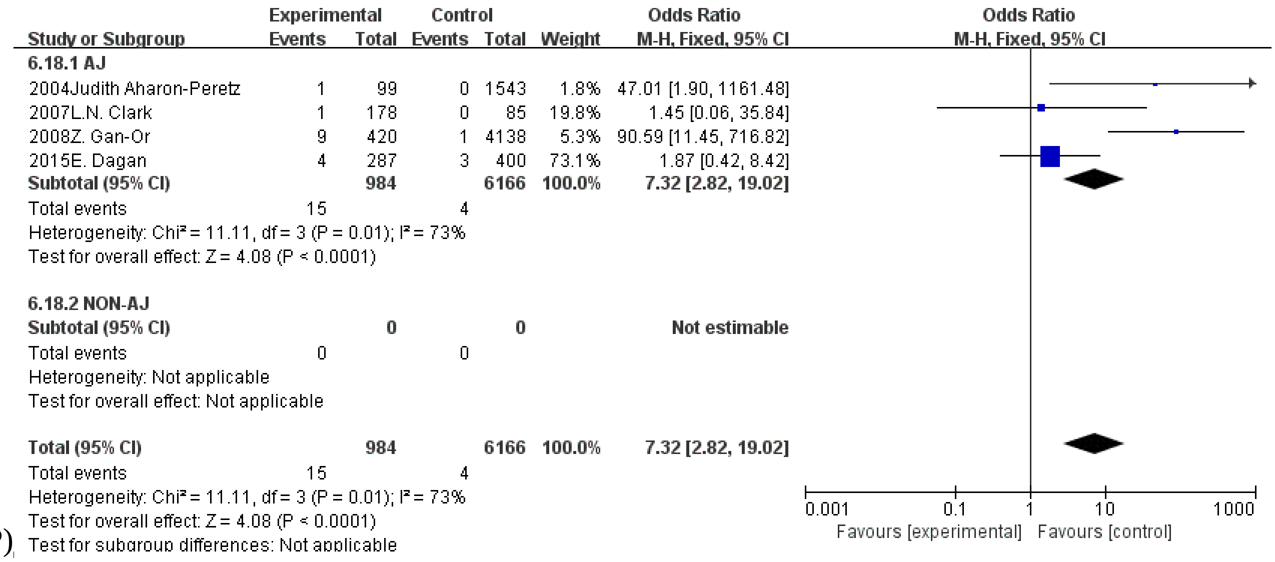
Experimental Control Odds Ratio Odds Ratio
Study or Subgroup Events Total Events Total Weight M-H, Fixed, 95% CI M-H, Fixed, 95% CI
6.18.1 AJ
2004Judith Aharon-Peretz 1 99 0 1543 1.8% 47.01 [1.90, 1161.48]
2007L.N. Clark 1 178 0 85 19.8% 1.45 [0.06, 35.84]
2008Z. Gan-Or 9 420 1 4138 5.3% 90.59 [11.45, 716.82]
2015E. Dagan 4 287 3 400 73.1% 1.87 [0.42, 8.42]
Subtotal (95% CI) 984 6166 100.0% 7.32 [2.82, 19.02]
Total events 15 4
Heterogeneity: Chi² = 11.11, df = 3 (P = 0.01); I² = 73%
Test for overall effect: Z = 4.08 (P < 0.0001)
6.18.2 NON-AJ
Subtotal (95% CI) 0 0 Not estimable
Total events 0 0
Heterogeneity: Not applicable
Test for overall effect: Not applicable
Total (95% CI) 984 6166 100.0% 7.32 [2.82, 19.02]
Total events 15 4
Heterogeneity: Chi² = 11.11, df = 3 (P = 0.01); I² = 73%
Test for overall effect: Z = 4.08 (P < 0.0001)
Test for subgroup differences: Not applicable
0.001 0.1 1 10 1000
Favours [experimental] Favours [control]
(P)

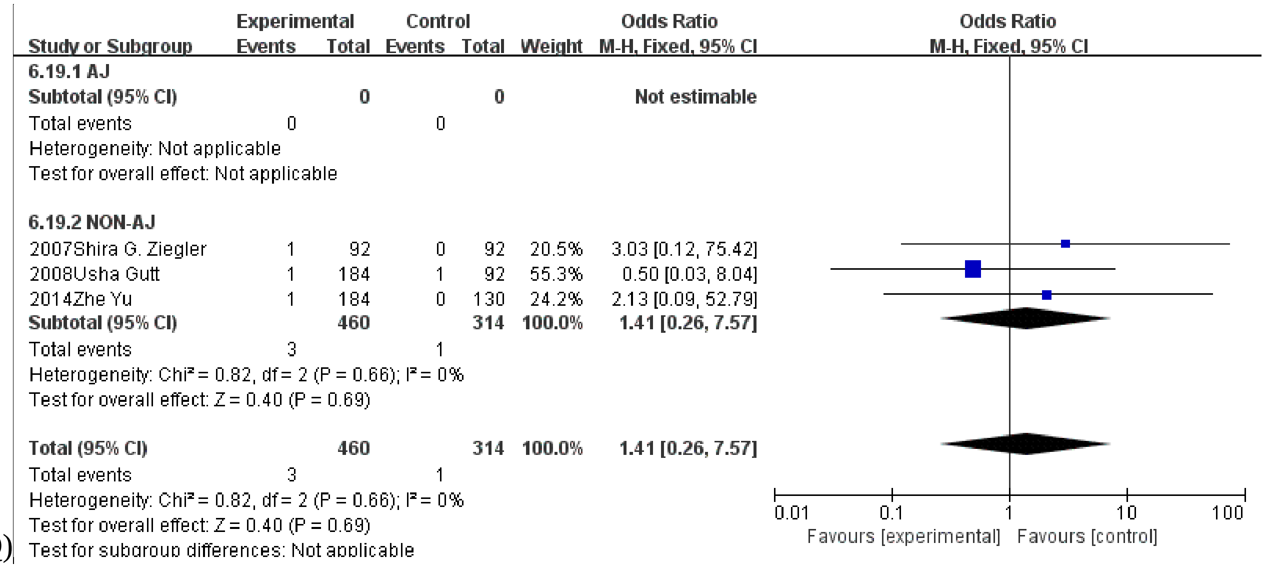
Experimental Control Odds Ratio Odds Ratio
Study or Subgroup Events Total Events Total Weight M-H, Fixed, 95% CI M-H, Fixed, 95% CI
6.19.1 AJ
Subtotal (95% CI) 0 0 Not estimable
Total events 0 0
Heterogeneity: Not applicable
Test for overall effect: Not applicable
6.19.2 NON-AJ
2007Shira G. Ziegler 1 92 0 92 20.5% 3.03 [0.12, 75.42]
2008Usha Gutt 1 184 1 92 55.3% 0.50 [0.03, 8.04]
2014Zhe Yu 1 184 0 130 24.2% 2.13 [0.09, 52.79]
Subtotal (95% CI) 460 314 100.0% 1.41 [0.26, 7.57]
Total events 3 1
Heterogeneity: Chi² = 0.82, df = 2 (P = 0.66); I² = 0%
Test for overall effect: Z = 0.40 (P = 0.69)
Total (95% CI) 460 314 100.0% 1.41 [0.26, 7.57]
Total events 3 1
Heterogeneity: Chi² = 0.82, df = 2 (P = 0.66); I² = 0%
Test for overall effect: Z = 0.40 (P = 0.69)
Test for subgroup differences: Not applicable
0.01 0.1 1 10 100
Favours [experimental] Favours [control]
(Q)

(R) 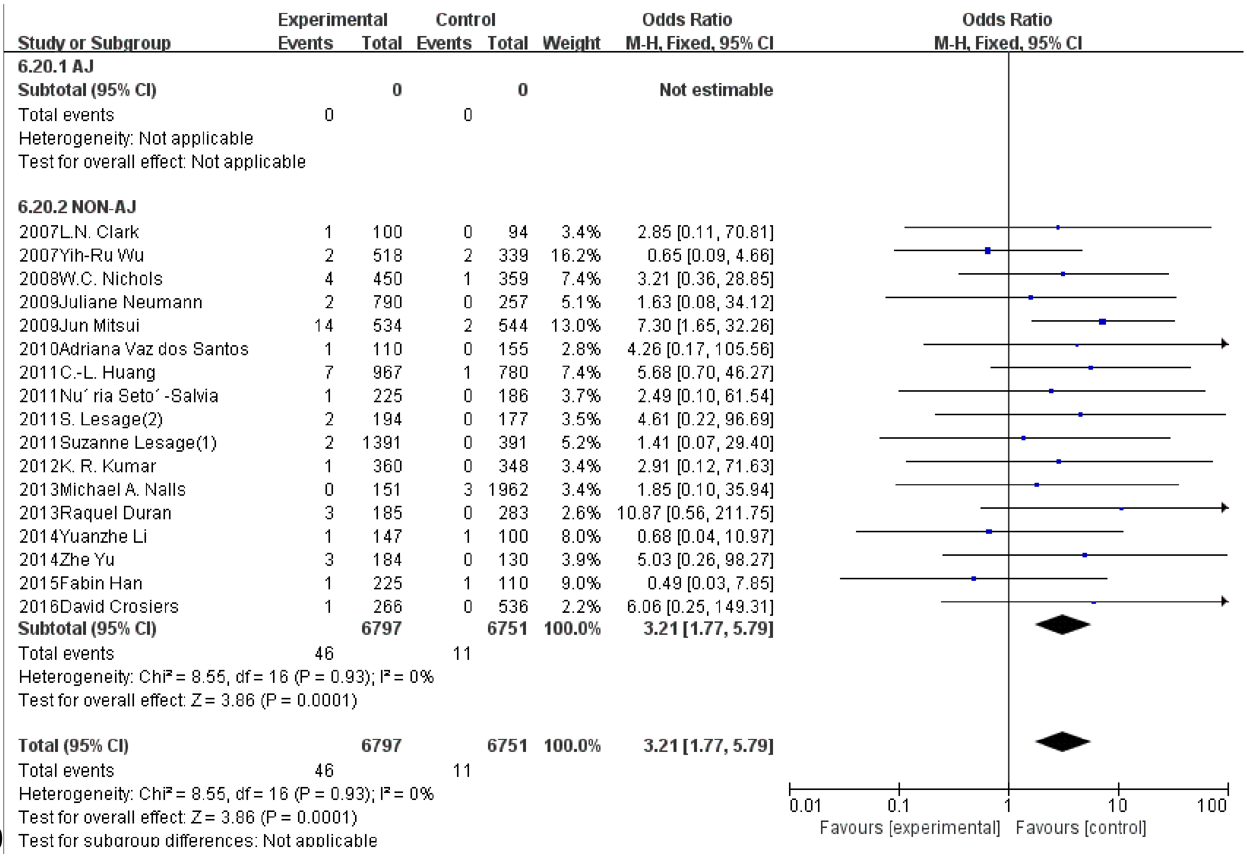

Supplementary Figure 1: Forest plots of the association between each *GBA* variant included and PD risks in total and by ethnicity (AJ and non-AJ). (A)-(R) were individually responsive to the variants 84insGG, IVS2+1G＞A, R120W, R131C, R163Q, H255Q, E326K, T369M, N370S, E388K, D409H, D443N, L444P, V460L/M/V, R463C, R496H, Q497R and RecNciI.
