# Supplementary material for: Integrated Genetic Analysis of Racial Differences of Common GBA Variants in Parkinson's Disease: A Meta-Analysis
Source: Front Mol Neurosci. 2018 Feb 15;11:43. doi: 10.3389/fnmol.2018.00043 (PMC5829555; doi:10.3389/fnmol.2018.00043)
Supplement: Supplementary file 6 [file Image2.pdf]

Supplementary Material

Integrated genetic analysis of racial differences of common GBA variants in parkinson's disease: a Meta-analysis

Yuan Zhang[1 †], Li Shu[1 †], Qiying Sun[2,3,4], Xun Zhou[1], Hongxu Pan[1], Jifeng Guo[1, 3,4], Beisha Tang[1, 3,4,5*]

[†] These authors have contributed equally to this work and are co-first authors.

[*] Correspondence: Beisha Tang bstang7398@163.com

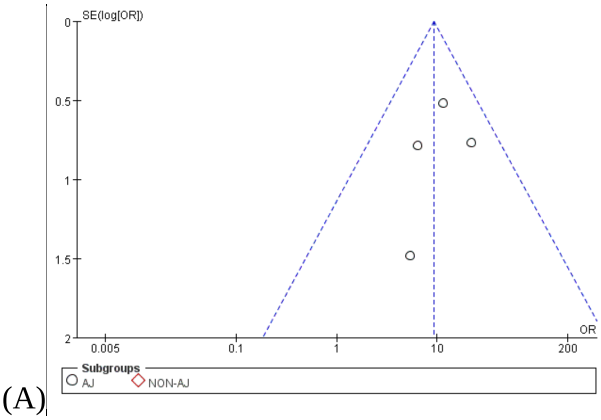

(A)

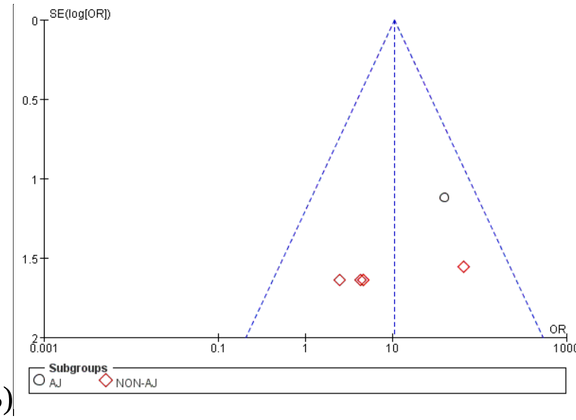

(B)

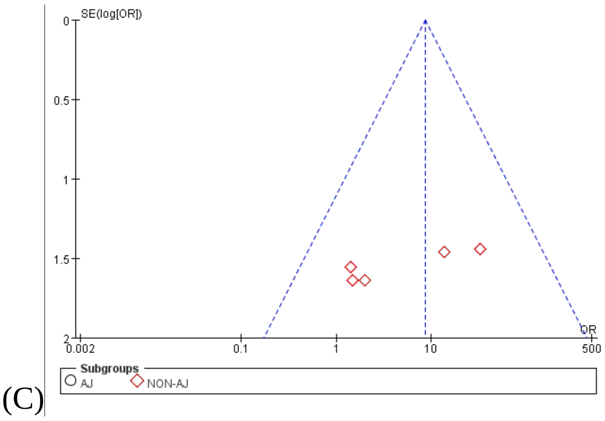

(C)

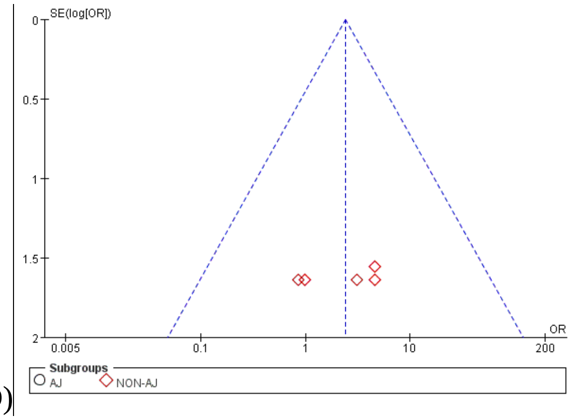

(D)

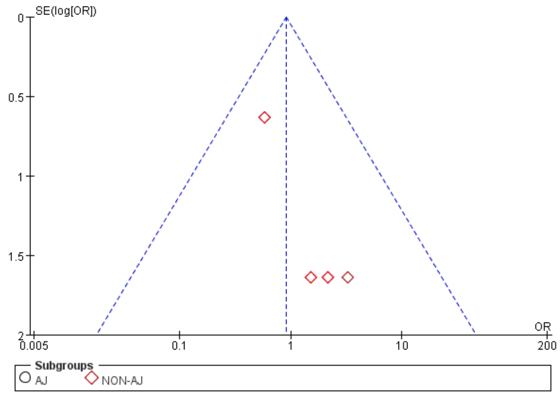
SE(log[OR])
0
0.5
1
1.5
2
0.005
0.1
1
10
200
OR
Subgroups
AJ
NON-AJ

(E)

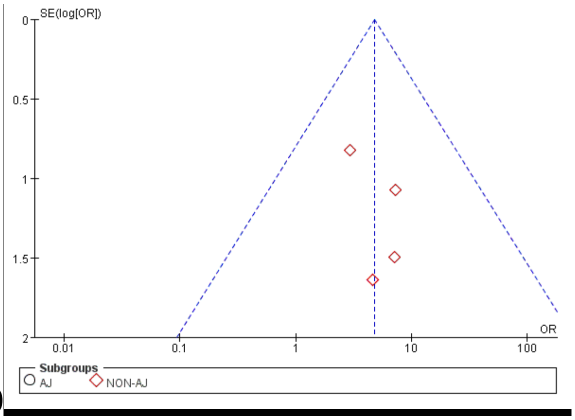
SE(log[OR])
0
0.5
1
1.5
2
0.01
0.1
1
10
100
OR
Subgroups
AJ
NON-AJ

(F)

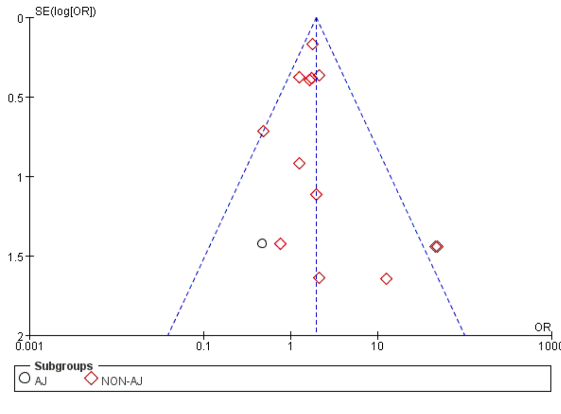
SE(log[OR])
0
0.5
1
1.5
2
0.001
0.1
1
10
1000
OR
Subgroups
AJ
NON-AJ

(G)

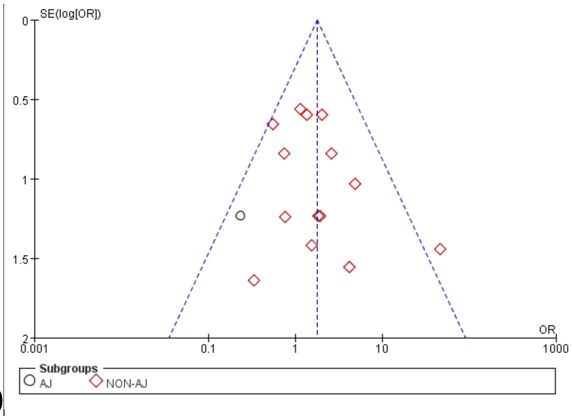
SE(log[OR])
0
0.5
1
1.5
2
0.001
0.1
1
10
1000
OR
Subgroups
AJ
NON-AJ

(H)

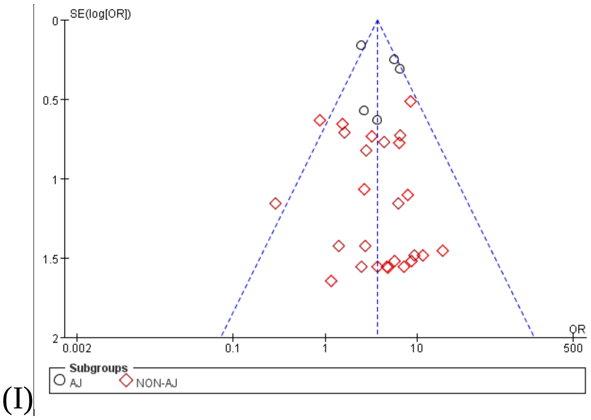
SE(log[OR])
0
0.5
1
1.5
2
0.002
0.1
1
10
500
OR
Subgroups
AJ
NON-AJ

(I)

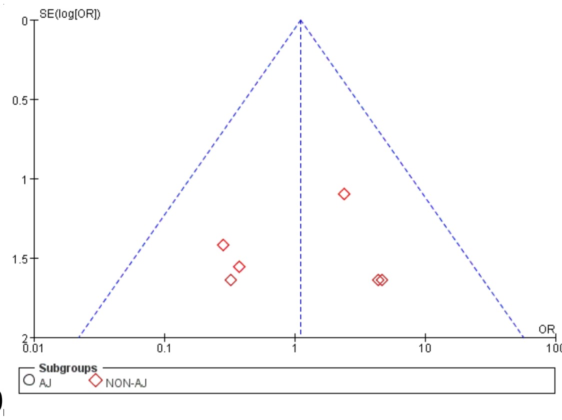
SE(log[OR])
0
0.5
1
1.5
2
0.01
0.1
1
10
100
OR
Subgroups
AJ
NON-AJ

(J)

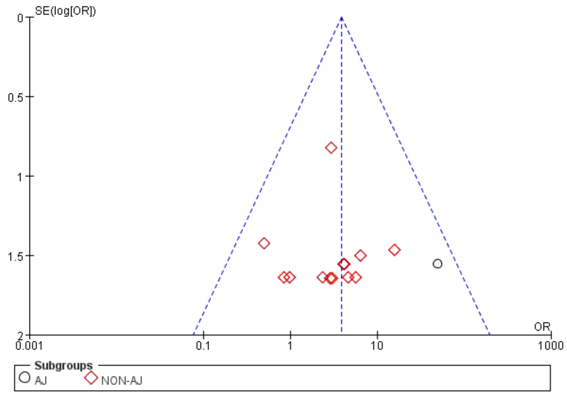
SE(log[OR])
0
0.5
1
1.5
2
0.001
0.1
1
10
1000
OR
Subgroups
AJ
NON-AJ

(K)

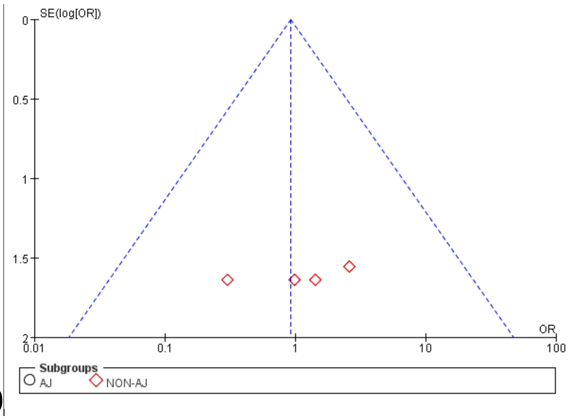
SE(log[OR])
0
0.5
1
1.5
2
0.01
0.1
1
10
100
OR
Subgroups
AJ
NON-AJ

(L)

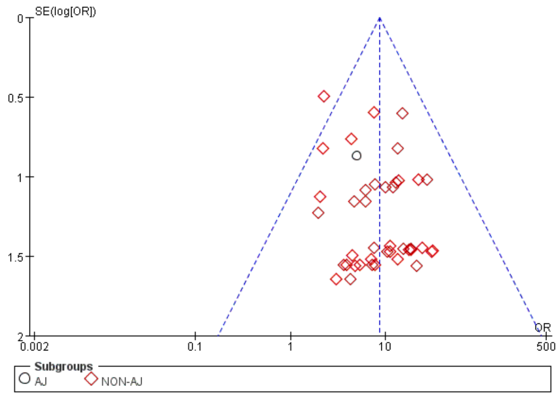

(M)

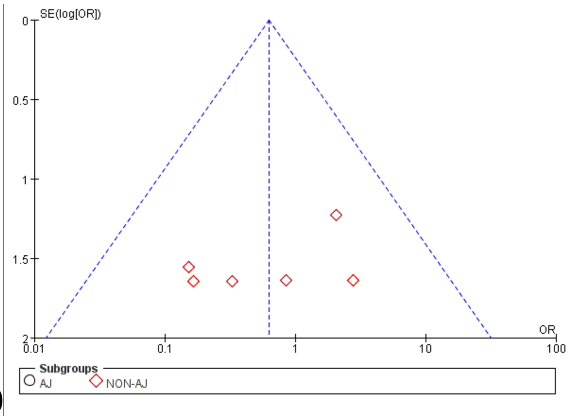

(N)

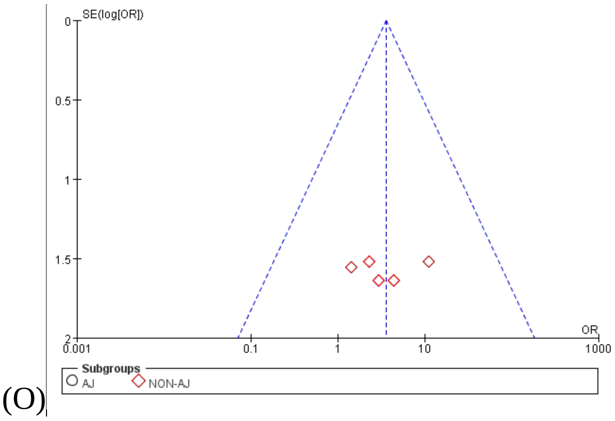

(O)

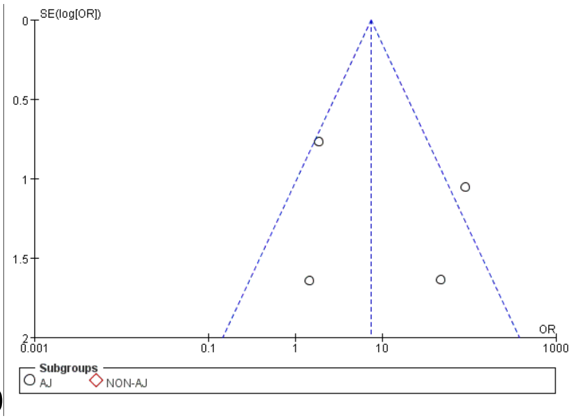

(P)

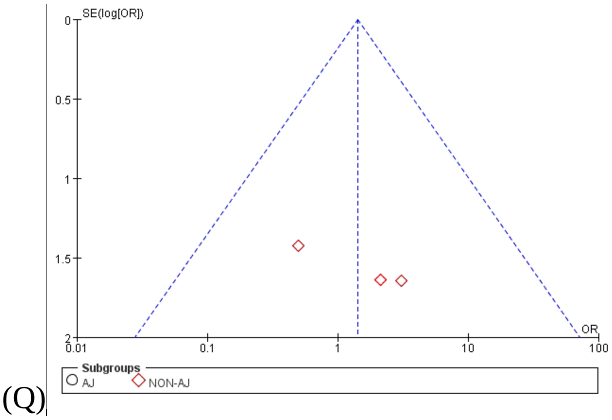

(Q)

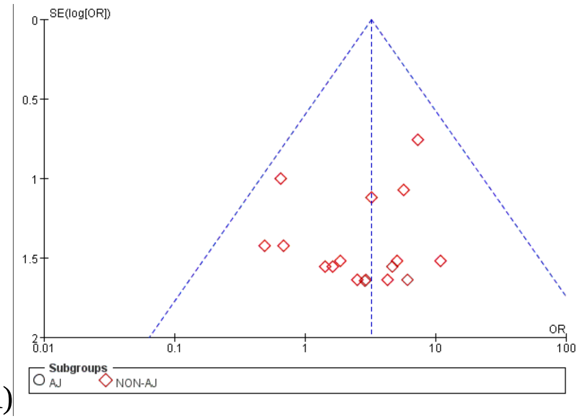

(R)

Supplementary Figure 2: Funnel plots of the association between each *GBA* variant and PD risks in total and by ethnicity (AJ and non-AJ). (A)-(R) were individually responsive to the variants 84insGG, IVS2+1G＞A, R120W, R131C, R163Q, H255Q, E326K, T369M, N370S, E388K, D409H, D443N, L444P, V460L/M/V, R463C, R496H, Q497R and RecNciI.
