# Supplementary material for: Integrated Genetic Analysis of Racial Differences of Common GBA Variants in Parkinson's Disease: A Meta-Analysis
Source: Front Mol Neurosci. 2018 Feb 15;11:43. doi: 10.3389/fnmol.2018.00043 (PMC5829555; doi:10.3389/fnmol.2018.00043)
Supplement: Supplementary file 7 [file Image3.pdf]

# *Supplementary Material*

# Integrated genetic analysis of racial differences of common GBA variants in Parkinson's disease: a Meta-analysis

**Yuan Zhang[1 †], Li Shu[1 †], Qiying Sun[2,3,4], Xun Zhou[1], Hongxu Pan[1], Jifeng Guo[1, 3,4], Beisha Tang[1, 3,4,5*]**

[†] These authors have contributed equally to this work and are co-first authors.

[*] **Correspondence: Beisha Tang bstang7398@163.com**

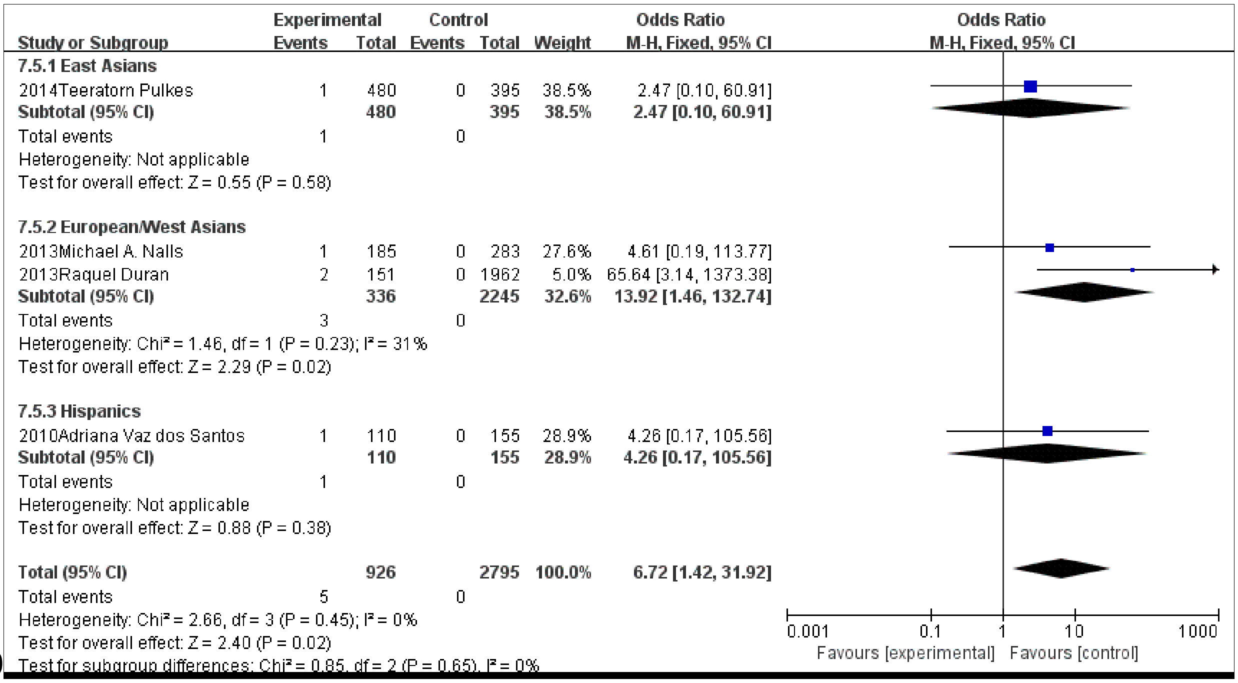

(A)

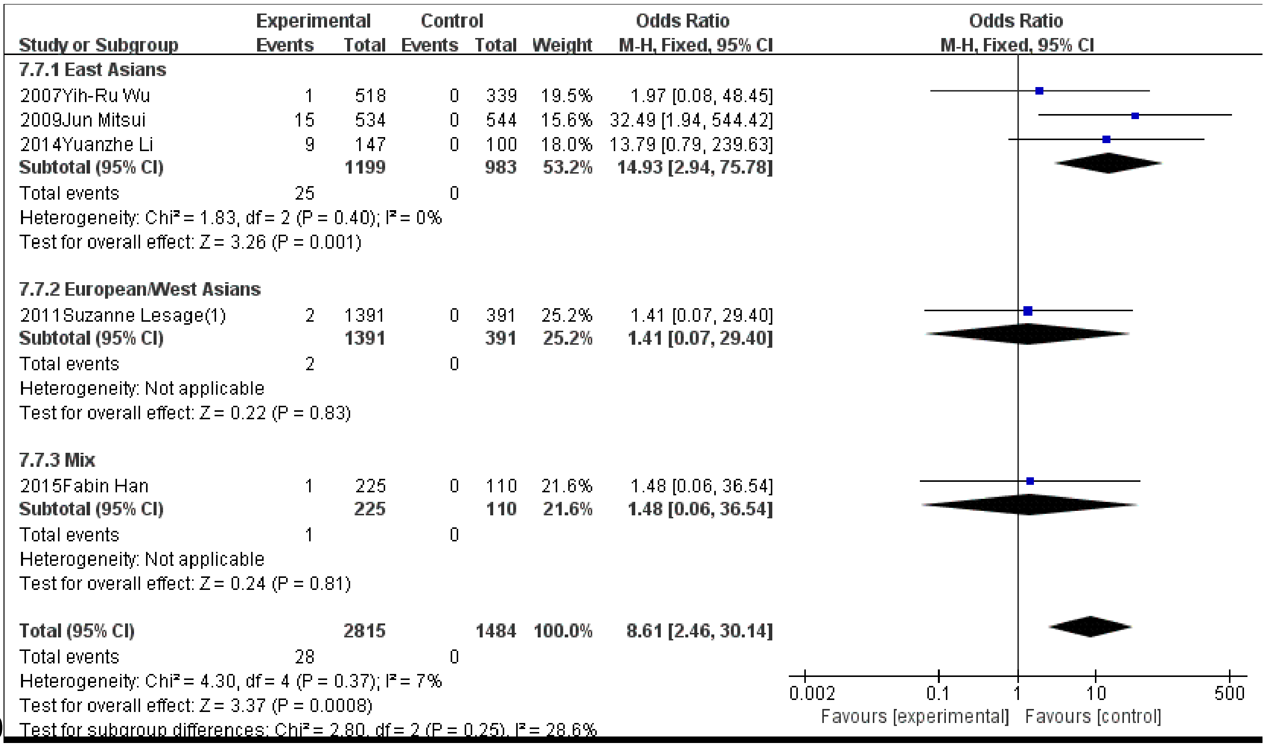
Experimental Control Odds Ratio Odds Ratio
Study or Subgroup Events Total Events Total Weight M-H, Fixed, 95% CI M-H, Fixed, 95% CI
7.7.1 East Asians
2007Yih-Ru Wu 1 518 0 339 19.5% 1.97 [0.08, 48.45]
2009Jun Mitsui 15 534 0 544 15.6% 32.49 [1.94, 544.42]
2014Yuanzhe Li 9 147 0 100 18.0% 13.79 [0.79, 239.63]
Subtotal (95% CI) 1199 983 53.2% 14.93 [2.94, 75.78]
Total events 25 0
Heterogeneity: Chi² = 1.83, df = 2 (P = 0.40); I² = 0%
Test for overall effect: Z = 3.26 (P = 0.001)
7.7.2 European/West Asians
2011Suzanne Lesage(1) 2 1391 0 391 25.2% 1.41 [0.07, 29.40]
Subtotal (95% CI) 1391 391 25.2% 1.41 [0.07, 29.40]
Total events 2 0
Heterogeneity: Not applicable
Test for overall effect: Z = 0.22 (P = 0.83)
7.7.3 Mix
2015Fabin Han 1 225 0 110 21.6% 1.48 [0.06, 36.54]
Subtotal (95% CI) 225 110 21.6% 1.48 [0.06, 36.54]
Total events 1 0
Heterogeneity: Not applicable
Test for overall effect: Z = 0.24 (P = 0.81)
Total (95% CI) 2815 1484 100.0% 8.61 [2.46, 30.14]
Total events 28 0
Heterogeneity: Chi² = 4.30, df = 4 (P = 0.37); I² = 7%
Test for overall effect: Z = 3.37 (P = 0.0008)
Test for subgroup differences: Chi² = 2.80, df = 2 (P = 0.25), I² = 28.6%
0.002 0.1 1 10 500
Favours [experimental] Favours [control]
(B)

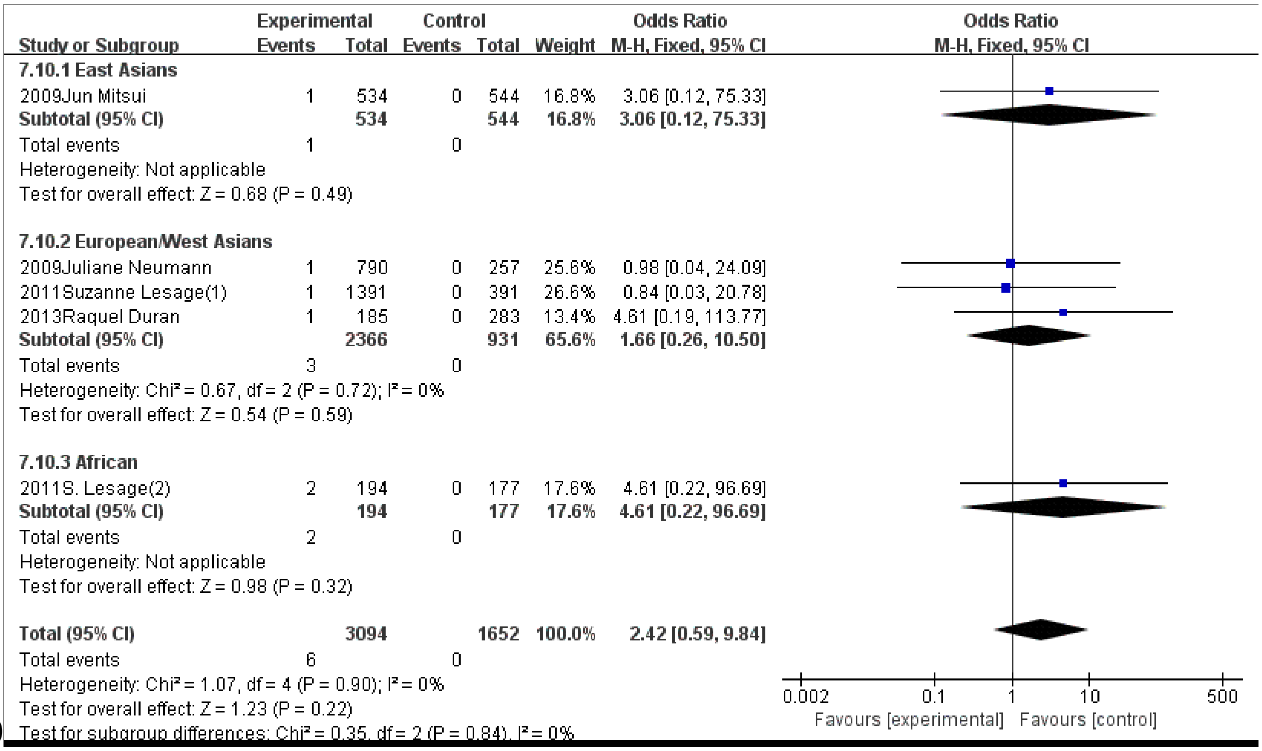
Experimental Control Odds Ratio Odds Ratio
Study or Subgroup Events Total Events Total Weight M-H, Fixed, 95% CI M-H, Fixed, 95% CI
7.10.1 East Asians
2009Jun Mitsui 1 534 0 544 16.8% 3.06 [0.12, 75.33]
Subtotal (95% CI) 534 544 16.8% 3.06 [0.12, 75.33]
Total events 1 0
Heterogeneity: Not applicable
Test for overall effect: Z = 0.68 (P = 0.49)
7.10.2 European/West Asians
2009Juliane Neumann 1 790 0 257 25.6% 0.98 [0.04, 24.09]
2011Suzanne Lesage(1) 1 1391 0 391 26.6% 0.84 [0.03, 20.78]
2013Raquel Duran 1 185 0 283 13.4% 4.61 [0.19, 113.77]
Subtotal (95% CI) 2366 931 65.6% 1.66 [0.26, 10.50]
Total events 3 0
Heterogeneity: Chi² = 0.67, df = 2 (P = 0.72); I² = 0%
Test for overall effect: Z = 0.54 (P = 0.59)
7.10.3 African
2011S. Lesage(2) 2 194 0 177 17.6% 4.61 [0.22, 96.69]
Subtotal (95% CI) 194 177 17.6% 4.61 [0.22, 96.69]
Total events 2 0
Heterogeneity: Not applicable
Test for overall effect: Z = 0.98 (P = 0.32)
Total (95% CI) 3094 1652 100.0% 2.42 [0.59, 9.84]
Total events 6 0
Heterogeneity: Chi² = 1.07, df = 4 (P = 0.90); I² = 0%
Test for overall effect: Z = 1.23 (P = 0.22)
Test for subgroup differences: Chi² = 0.35, df = 2 (P = 0.84), I² = 0%
0.002 0.1 1 10 500
Favours [experimental] Favours [control]
(C)

| Study or Subgroup | Experimental Events | Experimental Total | Control Events | Control Total | Weight | Odds Ratio M-H, Fixed, 95% CI |
|---|---|---|---|---|---|---|
| **7.12.1 East Asians** | | | | | | |
| 2008Usha Gutt | 1 | 184 | 0 | 92 | 7.7% | 1.51 [0.06, 37.49] |
| 2009Jun Mitsui | 4 | 534 | 7 | 544 | 80.0% | 0.58 [0.17, 1.99] |
| 2012Jung Mi Choi | 1 | 277 | 0 | 299 | 5.6% | 3.25 [0.13, 80.10] |
| 2014Zhe Yu | 1 | 184 | 0 | 130 | 6.8% | 2.13 [0.09, 52.79] |
| **Subtotal (95% CI)** | | **1179** | | **1065** | **100.0%** | **0.90 [0.34, 2.38]** |
| Total events | 7 | | 7 | | | |
| Heterogeneity: Chi² = 1.49, df = 3 (P = 0.69); I² = 0% | | | | | | |
| Test for overall effect: Z = 0.20 (P = 0.84) | | | | | | |
| **Total (95% CI)** | | **1179** | | **1065** | **100.0%** | **0.90 [0.34, 2.38]** |
| Total events | 7 | | 7 | | | |
| Heterogeneity: Chi² = 1.49, df = 3 (P = 0.69); I² = 0% | | | | | | |
| Test for overall effect: Z = 0.20 (P = 0.84) | | | | | | |
| Test for subgroup differences: Not applicable | | | | | | |

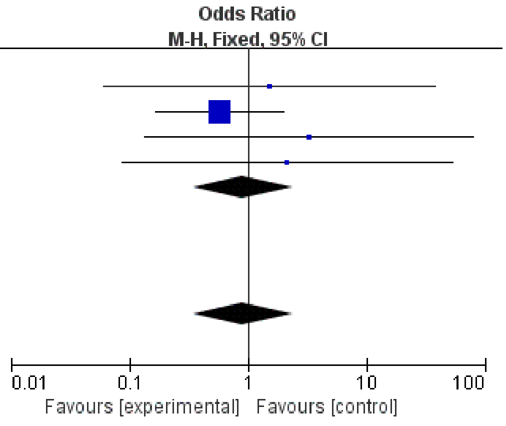


(D)

| Study or Subgroup | Experimental Events | Experimental Total | Control Events | Control Total | Weight | Odds Ratio M-H, Fixed, 95% CI |
|---|---|---|---|---|---|---|
| **7.14.2 European/West Asians** | | | | | | |
| 2009 Kallirhoe Kalinderi | 4 | 172 | 0 | 132 | 14.1% | 7.08 [0.38, 132.62] |
| 2011Marina Moraitou | 7 | 205 | 1 | 206 | 24.7% | 7.25 [0.88, 59.44] |
| 2012K. R. Kumar | 6 | 360 | 2 | 348 | 51.2% | 2.93 [0.59, 14.63] |
| 2013Raquel Duran | 1 | 185 | 0 | 283 | 10.0% | 4.61 [0.19, 113.77] |
| **Subtotal (95% CI)** | | **922** | | **969** | **100.0%** | **4.75 [1.60, 14.09]** |
| Total events | 18 | | 3 | | | |
| Heterogeneity: Chi² = 0.57, df = 3 (P = 0.90); I² = 0% | | | | | | |
| Test for overall effect: Z = 2.81 (P = 0.005) | | | | | | |
| **Total (95% CI)** | | **922** | | **969** | **100.0%** | **4.75 [1.60, 14.09]** |
| Total events | 18 | | 3 | | | |
| Heterogeneity: Chi² = 0.57, df = 3 (P = 0.90); I² = 0% | | | | | | |
| Test for overall effect: Z = 2.81 (P = 0.005) | | | | | | |
| Test for subgroup differences: Not applicable | | | | | | |

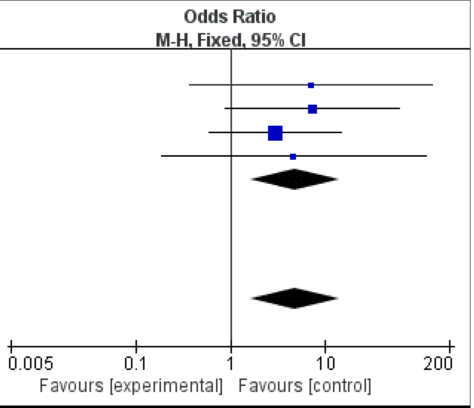


(E)

| Study or Subgroup | Experimental Events | Experimental Total | Control Events | Control Total | Weight | Odds Ratio M-H, Fixed, 95% CI |
|---|---|---|---|---|---|---|
| **7.17.1 East Asians** | | | | | | |
| 2007Shira G. Ziegler | 18 | 92 | 0 | 92 | 0.4% | 45.94 [2.72, 775.00] |
| 2014Zhe Yu | 1 | 184 | 0 | 130 | 0.5% | 2.13 [0.09, 52.79] |
| **Subtotal (95% CI)** | | **276** | | **222** | **0.9%** | **20.02 [2.83, 141.56]** |
| Total events | 19 | | 0 | | | |
| Heterogeneity: Chi² = 2.20, df = 1 (P = 0.14); I² = 55% | | | | | | |
| Test for overall effect: Z = 3.00 (P = 0.003) | | | | | | |
| **7.17.2 European/West Asians** | | | | | | |
| 2007Jose Bras | 2 | 230 | 3 | 430 | 1.9% | 1.25 [0.21, 7.53] |
| 2009 Kallirhoe Kalinderi | 1 | 172 | 1 | 132 | 1.0% | 0.77 [0.05, 12.36] |
| 2011Suzanne Lesage(1) | 49 | 1391 | 8 | 391 | 10.8% | 1.75 [0.82, 3.72] |
| 2013Raquel Duran | 14 | 185 | 0 | 283 | 0.3% | 47.94 [2.84, 808.75] |
| 2016Caroline Ran | 90 | 1625 | 65 | 2025 | 49.0% | 1.77 [1.28, 2.45] |
| 2016David Crosiers | 12 | 266 | 15 | 536 | 8.5% | 1.64 [0.76, 3.56] |
| 2016Silvia Jesús | 16 | 532 | 13 | 542 | 11.2% | 1.26 [0.60, 2.65] |
| **Subtotal (95% CI)** | | **4401** | | **4339** | **82.7%** | **1.84 [1.43, 2.37]** |
| Total events | 184 | | 105 | | | |
| Heterogeneity: Chi² = 6.84, df = 6 (P = 0.34); I² = 12% | | | | | | |
| Test for overall effect: Z = 4.77 (P < 0.00001) | | | | | | |
| **7.17.3 Hispanics** | | | | | | |
| 2007Mariana Spitz | 1 | 65 | 0 | 267 | 0.2% | 12.44 [0.50, 308.96] |
| **Subtotal (95% CI)** | | **65** | | **267** | **0.2%** | **12.44 [0.50, 308.96]** |
| Total events | 1 | | 0 | | | |
| Heterogeneity: Not applicable | | | | | | |
| Test for overall effect: Z = 1.54 (P = 0.12) | | | | | | |
| **7.17.4 Mix** | | | | | | |
| 2008W.C. Nichols | 28 | 450 | 11 | 359 | 10.3% | 2.10 [1.03, 4.28] |
| 2015Fabin Han | 4 | 225 | 4 | 110 | 4.7% | 0.48 [0.12, 1.96] |
| **Subtotal (95% CI)** | | **675** | | **469** | **15.0%** | **1.59 [0.85, 2.95]** |
| Total events | 32 | | 15 | | | |
| Heterogeneity: Chi² = 3.38, df = 1 (P = 0.07); I² = 70% | | | | | | |
| Test for overall effect: Z = 1.46 (P = 0.14) | | | | | | |
| **7.17.5 African** | | | | | | |
| 2017Melinda Barkhuizen | 5 | 105 | 1 | 40 | 1.2% | 1.95 [0.22, 17.23] |
| **Subtotal (95% CI)** | | **105** | | **40** | **1.2%** | **1.95 [0.22, 17.23]** |
| Total events | 5 | | 1 | | | |
| Heterogeneity: Not applicable | | | | | | |
| Test for overall effect: Z = 0.60 (P = 0.55) | | | | | | |
| **Total (95% CI)** | | **5522** | | **5337** | **100.0%** | **1.98 [1.58, 2.49]** |
| Total events | 241 | | 121 | | | |
| Heterogeneity: Chi² = 17.79, df = 12 (P = 0.12); I² = 33% | | | | | | |
| Test for overall effect: Z = 5.94 (P < 0.00001) | | | | | | |
| Test for subgroup differences: Chi² = 7.25, df = 4 (P = 0.12), I² = 44.9% | | | | | | |

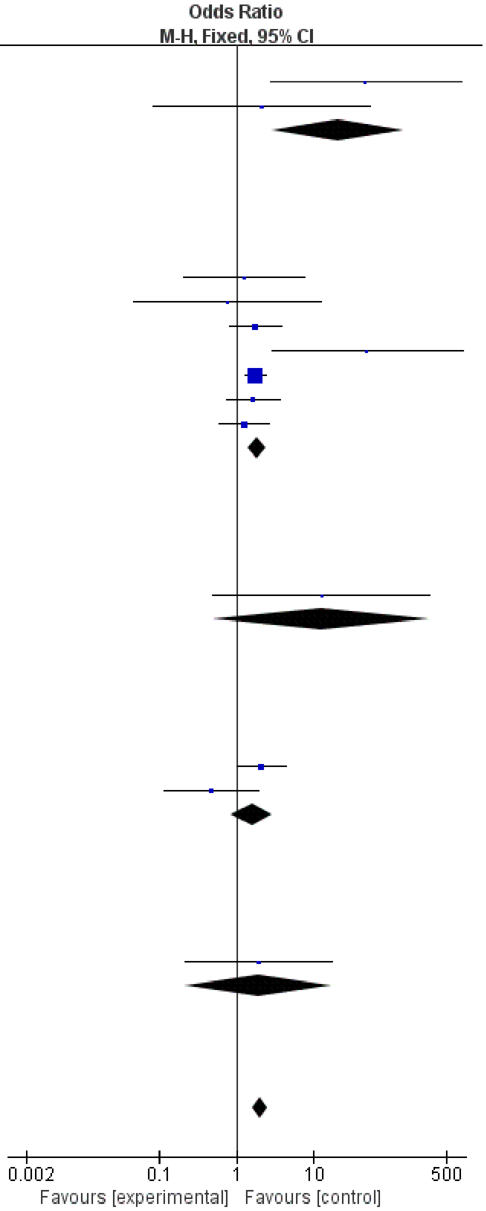


(F)

| | Experimental | | Control | | | Odds Ratio |
|---|---|---|---|---|---|---|
| **Study or Subgroup** | **Events** | **Total** | **Events** | **Total** | **Weight** | **M-H, Fixed, 95% CI** |
| **7.20.1 East Asians** | | | | | | |
| 2007Shira G. Ziegler | 18 | 92 | 0 | 92 | 1.1% | 45.94 [2.72, 775.00] |
| **Subtotal (95% CI)** | | **92** | | **92** | **1.1%** | **45.94 [2.72, 775.00]** |
| Total events | 18 | | 0 | | | |
| Heterogeneity: Not applicable | | | | | | |
| Test for overall effect: Z = 2.65 (P = 0.008) | | | | | | |
| **7.20.2 European/West Asians** | | | | | | |
| 2007Jose Bras | 2 | 230 | 5 | 430 | 9.5% | 0.75 [0.14, 3.87] |
| 2011Marina Moraitou | 0 | 205 | 1 | 206 | 4.1% | 0.33 [0.01, 8.23] |
| 2011Nu´ ria Seto´ -Salvia | 2 | 225 | 0 | 186 | 1.5% | 4.17 [0.20, 87.45] |
| 2011Suzanne Lesage(1) | 17 | 1391 | 1 | 391 | 4.2% | 4.83 [0.64, 36.37] |
| 2012K. R. Kumar | 7 | 360 | 6 | 348 | 16.5% | 1.13 [0.38, 3.40] |
| 2013Raquel Duran | 1 | 185 | 1 | 283 | 2.2% | 1.53 [0.10, 24.66] |
| 2016David Crosiers | 3 | 266 | 11 | 536 | 19.9% | 0.54 [0.15, 1.97] |
| 2016Silvia Jesús | 5 | 532 | 2 | 542 | 5.4% | 2.56 [0.49, 13.26] |
| **Subtotal (95% CI)** | | **3394** | | **2922** | **63.3%** | **1.29 [0.75, 2.23]** |
| Total events | 37 | | 27 | | | |
| Heterogeneity: Chi² = 5.79, df = 7 (P = 0.56); I² = 0% | | | | | | |
| Test for overall effect: Z = 0.92 (P = 0.36) | | | | | | |
| **7.20.3 Mix** | | | | | | |
| 2007L.N. Clark | 2 | 100 | 1 | 94 | 2.8% | 1.90 [0.17, 21.28] |
| 2008W.C. Nichols | 10 | 450 | 4 | 359 | 12.0% | 2.02 [0.63, 6.49] |
| 2015Fabin Han | 11 | 225 | 4 | 110 | 14.1% | 1.36 [0.42, 4.38] |
| **Subtotal (95% CI)** | | **775** | | **563** | **28.8%** | **1.69 [0.77, 3.68]** |
| Total events | 23 | | 9 | | | |
| Heterogeneity: Chi² = 0.23, df = 2 (P = 0.89); I² = 0% | | | | | | |
| Test for overall effect: Z = 1.31 (P = 0.19) | | | | | | |
| **7.20.4 African** | | | | | | |
| 2011S. Lesage(2) | 2 | 194 | 1 | 177 | 2.9% | 1.83 [0.16, 20.39] |
| 2017Melinda Barkhuizen | 2 | 105 | 1 | 40 | 3.9% | 0.76 [0.07, 8.59] |
| **Subtotal (95% CI)** | | **299** | | **217** | **6.8%** | **1.21 [0.22, 6.68]** |
| Total events | 4 | | 2 | | | |
| Heterogeneity: Chi² = 0.26, df = 1 (P = 0.61); I² = 0% | | | | | | |
| Test for overall effect: Z = 0.22 (P = 0.83) | | | | | | |
| **Total (95% CI)** | | **4560** | | **3794** | **100.0%** | **1.89 [1.27, 2.83]** |
| Total events | 82 | | 38 | | | |
| Heterogeneity: Chi² = 13.80, df = 13 (P = 0.39); I² = 6% | | | | | | |
| Test for overall effect: Z = 3.10 (P = 0.002) | | | | | | |
| Test for subgroup differences: Chi² = 6.07, df = 3 (P = 0.11), I² = 50.6% | | | | | | |

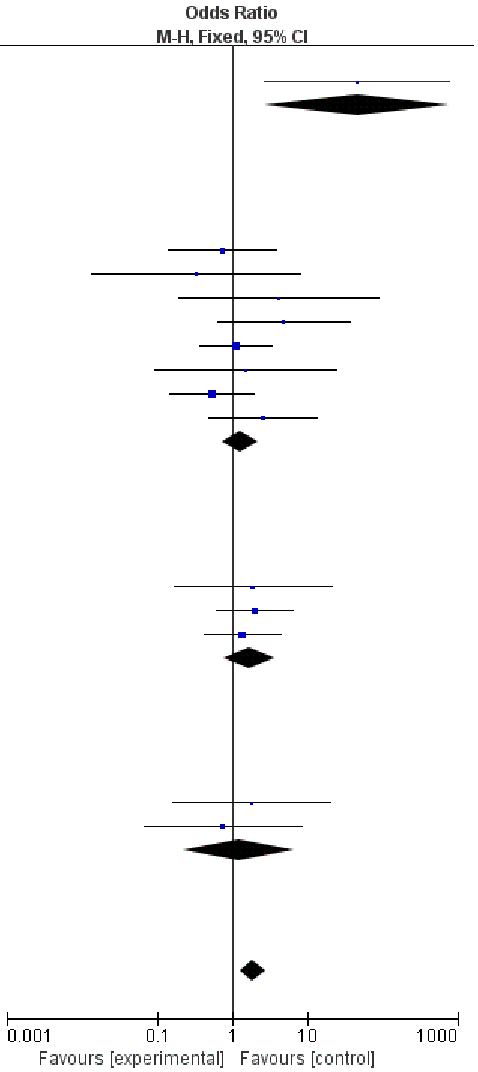


(G)

| Study or Subgroup | Experimental Events | Experimental Total | Control Events | Control Total | Weight | Odds Ratio M-H, Fixed, 95% CI |
|---|---|---|---|---|---|---|
| **7.23.1 East Asians** | | | | | | |
| 2010F.-Y. Hu | 6 | 328 | 2 | 300 | 4.8% | 2.78 [0.56, 13.86] |
| **Subtotal (95% CI)** | | **328** | | **300** | **4.8%** | **2.78 [0.56, 13.86]** |
| Total events | 6 | | 2 | | | |
| Heterogeneity: Not applicable | | | | | | |
| Test for overall effect: Z = 1.24 (P = 0.21) | | | | | | |
| **7.23.2 European/West Asians** | | | | | | |
| 2006M. Toft | 4 | 311 | 7 | 474 | 12.9% | 0.87 [0.25, 2.99] |
| 2007Elvira V. De Marco | 3 | 395 | 0 | 483 | 1.1% | 8.62 [0.44, 167.44] |
| 2007Jose Bras | 5 | 230 | 3 | 430 | 4.8% | 3.16 [0.75, 13.36] |
| 2008Ignacio F. Mata | 11 | 721 | 2 | 554 | 5.3% | 4.28 [0.94, 19.37] |
| 2009Juliane Neumann | 8 | 790 | 1 | 257 | 3.5% | 2.62 [0.33, 21.04] |
| 2011Marina Moraitou | 6 | 205 | 4 | 206 | 9.1% | 1.52 [0.42, 5.48] |
| 2011Nu´ ria Seto´ -Salvia | 5 | 225 | 0 | 186 | 1.3% | 9.30 [0.51, 169.36] |
| 2011Suzanne Lesage(1) | 45 | 1391 | 2 | 391 | 7.1% | 6.50 [1.57, 26.93] |
| 2012Anton Emelyanov | 2 | 330 | 0 | 240 | 1.4% | 3.66 [0.17, 76.60] |
| 2012K. R. Kumar | 9 | 360 | 0 | 348 | 1.2% | 18.84 [1.09, 324.91] |
| 2013Raquel Duran | 5 | 185 | 1 | 283 | 1.8% | 7.83 [0.91, 67.60] |
| 2014Rosanna Asselta | 69 | 2350 | 4 | 1111 | 12.4% | 8.37 [3.05, 23.00] |
| 2016Caroline Ran | 10 | 1625 | 2 | 2025 | 4.2% | 6.26 [1.37, 28.62] |
| 2016David Crosiers | 3 | 266 | 1 | 536 | 1.5% | 6.10 [0.63, 58.95] |
| 2016Silvia Jesús | 5 | 532 | 0 | 542 | 1.2% | 11.31 [0.62, 205.09] |
| **Subtotal (95% CI)** | | **9916** | | **8066** | **68.7%** | **4.85 [3.21, 7.32]** |
| Total events | 190 | | 27 | | | |
| Heterogeneity: Chi² = 14.46, df = 14 (P = 0.42); I² = 3% | | | | | | |
| Test for overall effect: Z = 7.50 (P < 0.00001) | | | | | | |
| **7.23.3 Hispanics** | | | | | | |
| 2010Adriana Vaz dos Santos | 2 | 110 | 0 | 155 | 1.0% | 7.17 [0.34, 150.74] |
| 2012Beatriz de Carvalho Guimarães | 3 | 237 | 0 | 186 | 1.3% | 5.57 [0.29, 108.45] |
| **Subtotal (95% CI)** | | **347** | | **341** | **2.3%** | **6.25 [0.74, 52.89]** |
| Total events | 5 | | 0 | | | |
| Heterogeneity: Chi² = 0.01, df = 1 (P = 0.91); I² = 0% | | | | | | |
| Test for overall effect: Z = 1.68 (P = 0.09) | | | | | | |
| **7.23.4 Mix** | | | | | | |
| 2005Christine Sato | 1 | 88 | 1 | 122 | 2.0% | 1.39 [0.09, 22.54] |
| 2007L.N. Clark | 2 | 100 | 0 | 94 | 1.2% | 4.80 [0.23, 101.23] |
| 2008W.C. Nichols | 6 | 450 | 3 | 359 | 7.8% | 1.60 [0.40, 6.46] |
| 2015Fabin Han | 2 | 225 | 0 | 110 | 1.6% | 2.47 [0.12, 51.93] |
| 2017Thomas R. Barber | 1 | 106 | 1 | 283 | 1.3% | 2.69 [0.17, 43.33] |
| **Subtotal (95% CI)** | | **969** | | **968** | **13.7%** | **2.05 [0.76, 5.54]** |
| Total events | 12 | | 5 | | | |
| Heterogeneity: Chi² = 0.54, df = 4 (P = 0.97); I² = 0% | | | | | | |
| Test for overall effect: Z = 1.41 (P = 0.16) | | | | | | |
| **7.23.5 African** | | | | | | |
| 2010Kenya Nishioka | 1 | 428 | 3 | 372 | 7.6% | 0.29 [0.03, 2.78] |
| 2011S. Lesage(2) | 2 | 194 | 0 | 177 | 1.2% | 4.61 [0.22, 96.69] |
| 2017Melinda Barkhuizen | 1 | 105 | 0 | 40 | 1.7% | 1.16 [0.05, 29.13] |
| **Subtotal (95% CI)** | | **727** | | **589** | **10.4%** | **0.93 [0.24, 3.55]** |
| Total events | 4 | | 3 | | | |
| Heterogeneity: Chi² = 2.11, df = 2 (P = 0.35); I² = 5% | | | | | | |
| Test for overall effect: Z = 0.10 (P = 0.92) | | | | | | |
| **Total (95% CI)** | | **12287** | | **10264** | **100.0%** | **3.98 [2.82, 5.63]** |
| Total events | 217 | | 37 | | | |
| Heterogeneity: Chi² = 22.36, df = 25 (P = 0.62); I² = 0% | | | | | | |
| Test for overall effect: Z = 7.83 (P < 0.00001) | | | | | | |
| Test for subgroup differences: Chi² = 7.41, df = 4 (P = 0.12), I² = 46.0% | | | | | | |

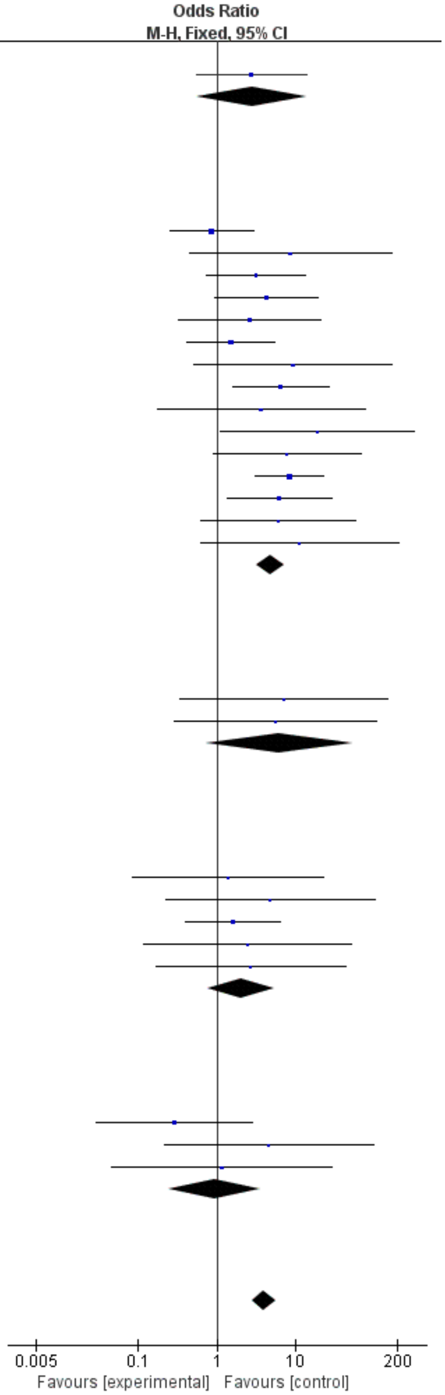


(H)

| Study or Subgroup | Experimental Events | Experimental Total | Control Events | Control Total | Weight | Odds Ratio M-H, Fixed, 95% CI |
|---|---|---|---|---|---|---|
| **7.25.2 European/West Asians** | | | | | | |
| 2007Jose Bras | 0 | 230 | 2 | 430 | 25.7% | 0.37 [0.02, 7.78] |
| 2011Suzanne Lesage(1) | 1 | 1391 | 1 | 391 | 23.0% | 0.28 [0.02, 4.50] |
| 2012K. R. Kumar | 0 | 360 | 1 | 348 | 22.4% | 0.32 [0.01, 7.91] |
| 2013Michael A. Nalls | 0 | 151 | 1 | 1962 | 3.2% | 4.32 [0.18, 106.39] |
| 2013Raquel Duran | 1 | 185 | 0 | 283 | 5.8% | 4.61 [0.19, 113.77] |
| 2014Rosanna Asselta | 5 | 2350 | 1 | 1111 | 20.0% | 2.37 [0.28, 20.28] |
| **Subtotal (95% CI)** | | **4667** | | **4525** | **100.0%** | **1.11 [0.39, 3.13]** |
| Total events | 7 | | 6 | | | |
| Heterogeneity: Chi² = 3.94, df = 5 (P = 0.56); I² = 0% | | | | | | |
| Test for overall effect: Z = 0.19 (P = 0.85) | | | | | | |
| **Total (95% CI)** | | **4667** | | **4525** | **100.0%** | **1.11 [0.39, 3.13]** |
| Total events | 7 | | 6 | | | |
| Heterogeneity: Chi² = 3.94, df = 5 (P = 0.56); I² = 0% | | | | | | |
| Test for overall effect: Z = 0.19 (P = 0.85) | | | | | | |
| Test for subgroup differences: Not applicable | | | | | | |

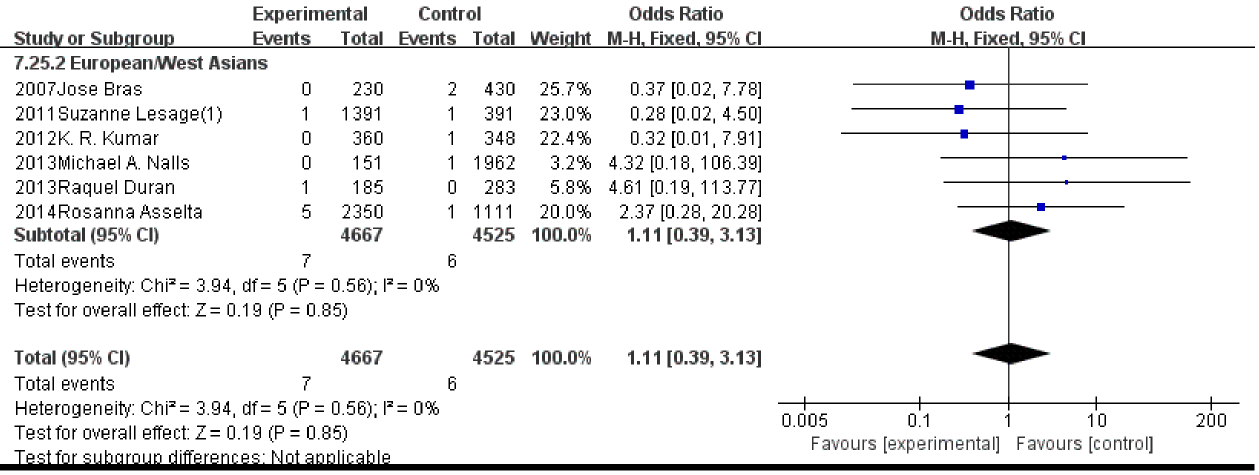


(I)

| Study or Subgroup | Experimental Events | Experimental Total | Control Events | Control Total | Weight | Odds Ratio M-H, Fixed, 95% CI |
|---|---|---|---|---|---|---|
| **7.27.1 East Asians** | | | | | | |
| 2007Shira G. Ziegler | 1 | 92 | 0 | 92 | 5.3% | 3.03 [0.12, 75.42] |
| 2008Usha Gutt | 1 | 184 | 1 | 92 | 14.2% | 0.50 [0.03, 8.04] |
| 2011C.-L. Huang | 2 | 967 | 0 | 780 | 5.9% | 4.04 [0.19, 84.31] |
| 2014Yuanzhe Li | 4 | 147 | 0 | 100 | 6.2% | 6.30 [0.34, 118.38] |
| **Subtotal (95% CI)** | | **1390** | | **1064** | **31.7%** | **2.72 [0.68, 10.86]** |
| Total events | 8 | | 1 | | | |
| Heterogeneity: Chi² = 1.82, df = 3 (P = 0.61); I² = 0% | | | | | | |
| Test for overall effect: Z = 1.42 (P = 0.16) | | | | | | |
| **7.27.2 European/West Asians** | | | | | | |
| 2007Jose Bras | 1 | 230 | 0 | 430 | 3.7% | 5.63 [0.23, 138.69] |
| 2009 Kallirhoe Kalinderi | 1 | 172 | 0 | 132 | 6.0% | 2.32 [0.09, 57.35] |
| 2009Juliane Neumann | 1 | 790 | 0 | 257 | 8.1% | 0.98 [0.04, 24.09] |
| 2011Marina Moraitou | 7 | 205 | 0 | 206 | 5.2% | 15.60 [0.89, 275.03] |
| 2011Nu´ria Seto´-Salvia | 2 | 225 | 0 | 186 | 5.8% | 4.17 [0.20, 87.45] |
| 2011Suzanne Lesage(1) | 1 | 1391 | 0 | 391 | 8.4% | 0.84 [0.03, 20.78] |
| 2012K. R. Kumar | 6 | 360 | 2 | 348 | 21.5% | 2.93 [0.59, 14.63] |
| 2013Raquel Duran | 1 | 185 | 0 | 283 | 4.2% | 4.61 [0.19, 113.77] |
| **Subtotal (95% CI)** | | **3558** | | **2233** | **62.9%** | **3.77 [1.49, 9.53]** |
| Total events | 20 | | 2 | | | |
| Heterogeneity: Chi² = 2.72, df = 7 (P = 0.91); I² = 0% | | | | | | |
| Test for overall effect: Z = 2.81 (P = 0.005) | | | | | | |
| **7.27.3 Mix** | | | | | | |
| 2007L.N. Clark | 1 | 100 | 0 | 94 | 5.5% | 2.85 [0.11, 70.81] |
| **Subtotal (95% CI)** | | **100** | | **94** | **5.5%** | **2.85 [0.11, 70.81]** |
| Total events | 1 | | 0 | | | |
| Heterogeneity: Not applicable | | | | | | |
| Test for overall effect: Z = 0.64 (P = 0.52) | | | | | | |
| **Total (95% CI)** | | **5048** | | **3391** | **100.0%** | **3.39 [1.60, 7.16]** |
| Total events | 29 | | 3 | | | |
| Heterogeneity: Chi² = 4.65, df = 12 (P = 0.97); I² = 0% | | | | | | |
| Test for overall effect: Z = 3.20 (P = 0.001) | | | | | | |
| Test for subgroup differences: Chi² = 0.16, df = 2 (P = 0.92), I² = 0% | | | | | | |

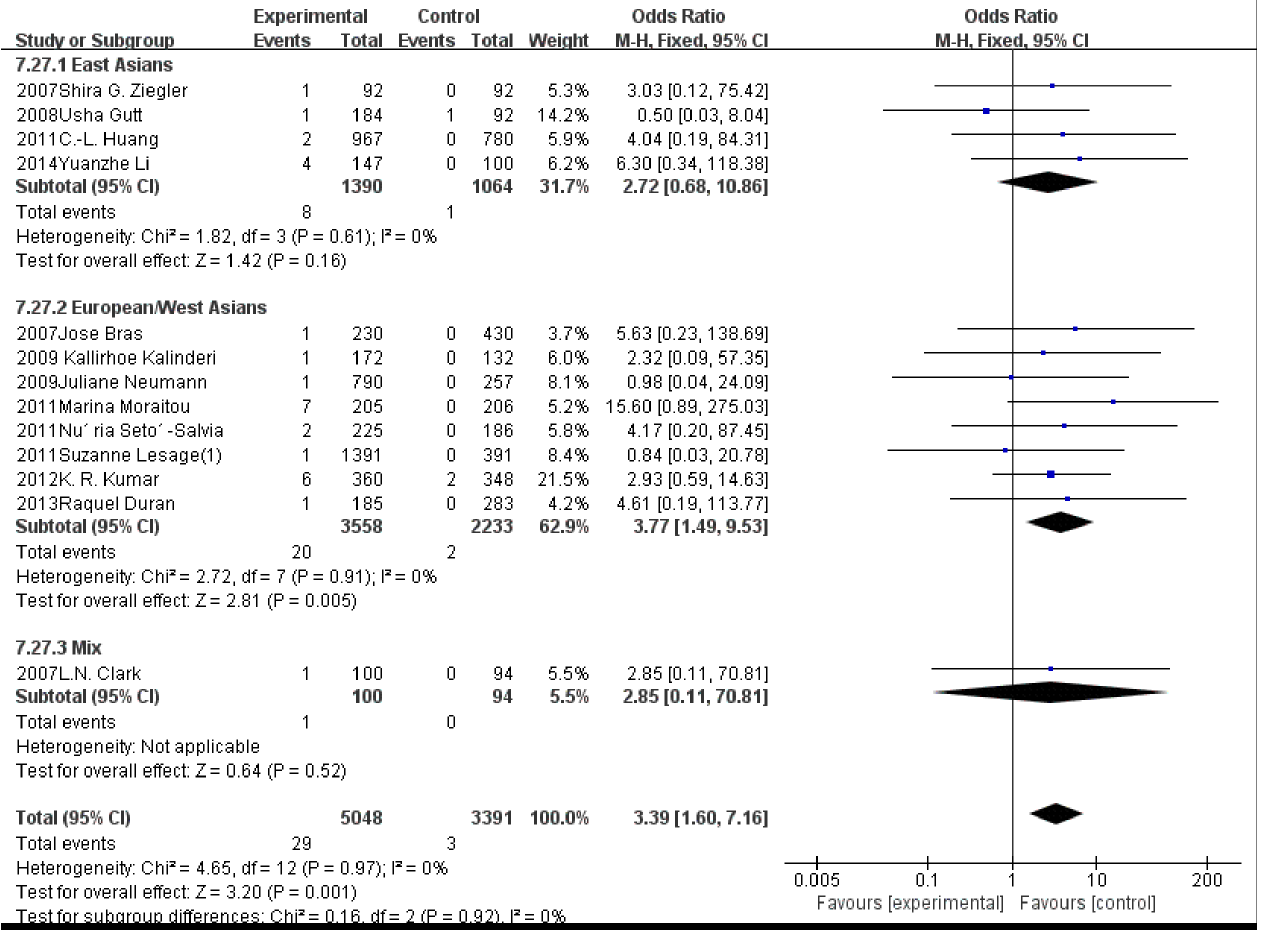


(J)

| Study or Subgroup | Experimental Events | Experimental Total | Control Events | Control Total | Weight | Odds Ratio M-H, Fixed, 95% CI |
|---|---|---|---|---|---|---|
| **7.29.1 European/West Asians** | | | | | | |
| 2009Juliane Neumann | 1 | 790 | 0 | 257 | 22.4% | 0.98 [0.04, 24.09] |
| 2013Michael A. Nalls | 0 | 151 | 2 | 1962 | 10.7% | 2.59 [0.12, 54.15] |
| 2014Rosanna Asselta | 1 | 2350 | 0 | 1111 | 20.2% | 1.42 [0.06, 34.87] |
| **Subtotal (95% CI)** | | **3291** | | **3330** | **53.4%** | **1.47 [0.22, 9.94]** |
| Total events | 2 | | 2 | | | |
| Heterogeneity: Chi² = 0.20, df = 2 (P = 0.91); I² = 0% | | | | | | |
| Test for overall effect: Z = 0.39 (P = 0.69) | | | | | | |
| **7.29.2 African** | | | | | | |
| 2011S. Lesage(2) | 0 | 194 | 1 | 177 | 46.6% | 0.30 [0.01, 7.47] |
| **Subtotal (95% CI)** | | **194** | | **177** | **46.6%** | **0.30 [0.01, 7.47]** |
| Total events | 0 | | 1 | | | |
| Heterogeneity: Not applicable | | | | | | |
| Test for overall effect: Z = 0.73 (P = 0.46) | | | | | | |
| **Total (95% CI)** | | **3485** | | **3507** | **100.0%** | **0.92 [0.20, 4.32]** |
| Total events | 2 | | 3 | | | |
| Heterogeneity: Chi² = 0.98, df = 3 (P = 0.81); I² = 0% | | | | | | |
| Test for overall effect: Z = 0.10 (P = 0.92) | | | | | | |
| Test for subgroup differences: Chi² = 0.69, df = 1 (P = 0.41), I² = 0% | | | | | | |

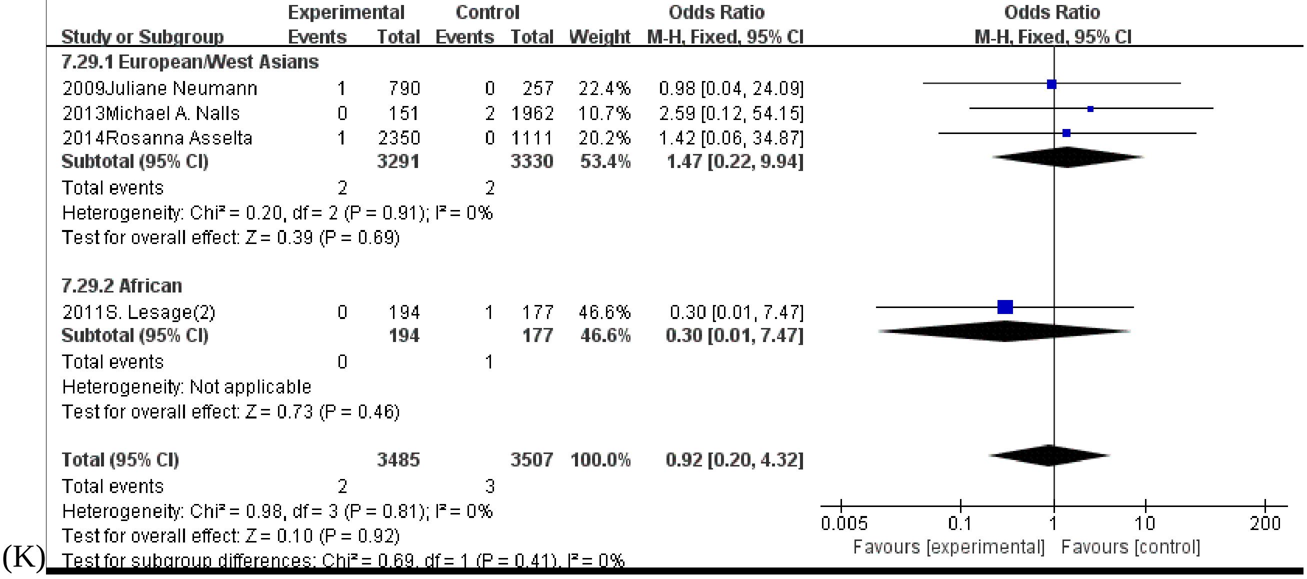


(K)

| Study or Subgroup | Experimental Events | Experimental Total | Control Events | Control Total | Weight | Odds Ratio M-H, Fixed, 95% CI |
|---|---|---|---|---|---|---|
| **7.31.1 East Asians** | | | | | | |
| 2007Eng-King Tan | 8 | 331 | 0 | 347 | 1.2% | 18.26 [1.05, 317.65] |
| 2007Shira G. Ziegler | 1 | 92 | 0 | 92 | 1.2% | 3.03 [0.12, 75.42] |
| 2007Yih-Ru Wu | 13 | 518 | 2 | 339 | 5.8% | 4.34 [0.97, 19.34] |
| 2008Usha Gutt | 4 | 184 | 1 | 92 | 3.2% | 2.02 [0.22, 18.36] |
| 2009Jun Mitsui | 8 | 534 | 0 | 544 | 1.2% | 17.58 [1.01, 305.37] |
| 2010Qi-Ying Sun | 11 | 402 | 0 | 413 | 1.2% | 24.29 [1.43, 413.63] |
| 2010X.-Y. Mao | 20 | 616 | 1 | 411 | 2.8% | 13.76 [1.84, 102.92] |
| 2011C.-L. Huang | 27 | 967 | 1 | 780 | 2.6% | 22.38 [3.03, 165.03] |
| 2012Jung Mi Choi | 2 | 277 | 0 | 291 | 1.2% | 5.29 [0.25, 110.69] |
| 2012Xiong Zhang | 6 | 195 | 0 | 443 | 0.7% | 30.42 [1.71, 542.79] |
| 2012Youpei Wang | 8 | 208 | 1 | 298 | 1.9% | 11.88 [1.47, 95.72] |
| 2014Teeratorn Pulkes | 15 | 480 | 1 | 395 | 2.6% | 12.71 [1.67, 96.65] |
| 2014Yuanzhe Li | 12 | 147 | 0 | 100 | 1.3% | 18.54 [1.09, 316.88] |
| 2014Zhe Yu | 2 | 184 | 0 | 130 | 1.4% | 3.58 [0.17, 75.09] |
| 2015Guo ji-feng | 26 | 1019 | 1 | 1030 | 2.4% | 26.94 [3.65, 198.92] |
| **Subtotal (95% CI)** | | **6154** | | **5705** | **30.6%** | **12.43 [6.92, 22.31]** |
| Total events | 163 | | 8 | | | |
| Heterogeneity: Chi² = 7.90, df = 14 (P = 0.89); I² = 0% | | | | | | |
| Test for overall effect: Z = 8.44 (P < 0.00001) | | | | | | |
| **7.31.2 European/West Asians** | | | | | | |
| 2006M. Toft | 3 | 311 | 1 | 474 | 1.9% | 4.61 [0.48, 44.49] |
| 2007Elvira V. De Marco | 8 | 395 | 1 | 483 | 2.2% | 9.96 [1.24, 80.01] |
| 2007Jose Bras | 3 | 230 | 0 | 430 | 0.8% | 13.25 [0.68, 257.56] |
| 2008Ignacio F. Mata | 10 | 721 | 1 | 554 | 2.7% | 7.78 [0.99, 60.94] |
| 2009 Kallirhoe Kalinderi | 2 | 172 | 0 | 132 | 1.4% | 3.89 [0.18, 81.62] |
| 2009Juliane Neumann | 11 | 790 | 0 | 257 | 1.8% | 7.60 [0.45, 129.38] |
| 2011Marina Moraitou | 6 | 205 | 1 | 206 | 2.4% | 6.18 [0.74, 51.80] |
| 2011Nu´ ria Seto´ -Salvia | 6 | 225 | 0 | 186 | 1.3% | 11.05 [0.62, 197.37] |
| 2011Suzanne Lesage(1) | 19 | 1391 | 0 | 391 | 1.9% | 11.12 [0.67, 184.66] |
| 2012Anton Emelyanov | 6 | 330 | 2 | 240 | 5.5% | 2.20 [0.44, 11.01] |
| 2012K. R. Kumar | 2 | 360 | 1 | 348 | 2.5% | 1.94 [0.17, 21.48] |
| 2013Michael A. Nalls | 3 | 151 | 3 | 1962 | 1.0% | 13.24 [2.65, 66.15] |
| 2013Raquel Duran | 2 | 185 | 0 | 283 | 1.0% | 7.72 [0.37, 161.82] |
| 2014Rosanna Asselta | 47 | 2350 | 3 | 1111 | 9.7% | 7.54 [2.34, 24.27] |
| 2016Caroline Ran | 35 | 1625 | 3 | 2025 | 6.4% | 14.84 [4.55, 48.33] |
| 2016David Crosiers | 3 | 266 | 1 | 536 | 1.6% | 6.10 [0.63, 58.95] |
| 2016Rita Török | 3 | 124 | 0 | 122 | 1.2% | 7.06 [0.36, 138.09] |
| 2016Silvia Jesús | 13 | 532 | 6 | 542 | 14.1% | 2.24 [0.84, 5.93] |
| **Subtotal (95% CI)** | | **10363** | | **10282** | **59.4%** | **6.52 [4.23, 10.05]** |
| Total events | 182 | | 23 | | | |
| Heterogeneity: Chi² = 10.92, df = 17 (P = 0.86); I² = 0% | | | | | | |
| Test for overall effect: Z = 8.50 (P < 0.00001) | | | | | | |
| **7.31.3 Hispanics** | | | | | | |
| 2007Mariana Spitz | 2 | 65 | 0 | 267 | 0.5% | 21.06 [1.00, 444.16] |
| 2010Adriana Vaz dos Santos | 2 | 110 | 0 | 155 | 1.0% | 7.17 [0.34, 150.74] |
| 2012Beatriz de Carvalho Guimarães | 6 | 237 | 0 | 186 | 1.3% | 10.47 [0.59, 187.11] |
| 2013M de L Gonz´ alez-del Rinc´ on | 7 | 128 | 0 | 252 | 0.8% | 31.17 [1.77, 550.28] |
| **Subtotal (95% CI)** | | **540** | | **860** | **3.6%** | **15.44 [3.44, 69.33]** |
| Total events | 17 | | 0 | | | |
| Heterogeneity: Chi² = 0.58, df = 3 (P = 0.90); I² = 0% | | | | | | |
| Test for overall effect: Z = 3.57 (P = 0.0004) | | | | | | |
| **7.31.4 Mix** | | | | | | |
| 2005Christine Sato | 1 | 88 | 0 | 122 | 1.0% | 4.20 [0.17, 104.32] |
| 2007L.N. Clark | 2 | 100 | 0 | 94 | 1.2% | 4.80 [0.23, 101.23] |
| 2008W.C. Nichols | 9 | 450 | 0 | 359 | 1.3% | 15.47 [0.90, 266.72] |
| 2015Fabin Han | 4 | 225 | 0 | 110 | 1.6% | 4.49 [0.24, 84.14] |
| **Subtotal (95% CI)** | | **863** | | **685** | **5.2%** | **7.33 [1.66, 32.37]** |
| Total events | 16 | | 0 | | | |
| Heterogeneity: Chi² = 0.56, df = 3 (P = 0.91); I² = 0% | | | | | | |
| Test for overall effect: Z = 2.63 (P = 0.009) | | | | | | |
| **7.31.5 African** | | | | | | |
| 2011S. Lesage(2) | 2 | 194 | 0 | 177 | 1.3% | 4.61 [0.22, 96.69] |
| **Subtotal (95% CI)** | | **194** | | **177** | **1.3%** | **4.61 [0.22, 96.69]** |
| Total events | 2 | | 0 | | | |
| Heterogeneity: Not applicable | | | | | | |
| Test for overall effect: Z = 0.98 (P = 0.32) | | | | | | |
| **Total (95% CI)** | | **18114** | | **17709** | **100.0%** | **8.66 [6.25, 12.01]** |
| Total events | 380 | | 31 | | | |
| Heterogeneity: Chi² = 23.60, df = 41 (P = 0.99); I² = 0% | | | | | | |
| Test for overall effect: Z = 12.95 (P < 0.00001) | | | | | | |
| Test for subgroup differences: Chi² = 3.85, df = 4 (P = 0.43), I² = 0% | | | | | | |

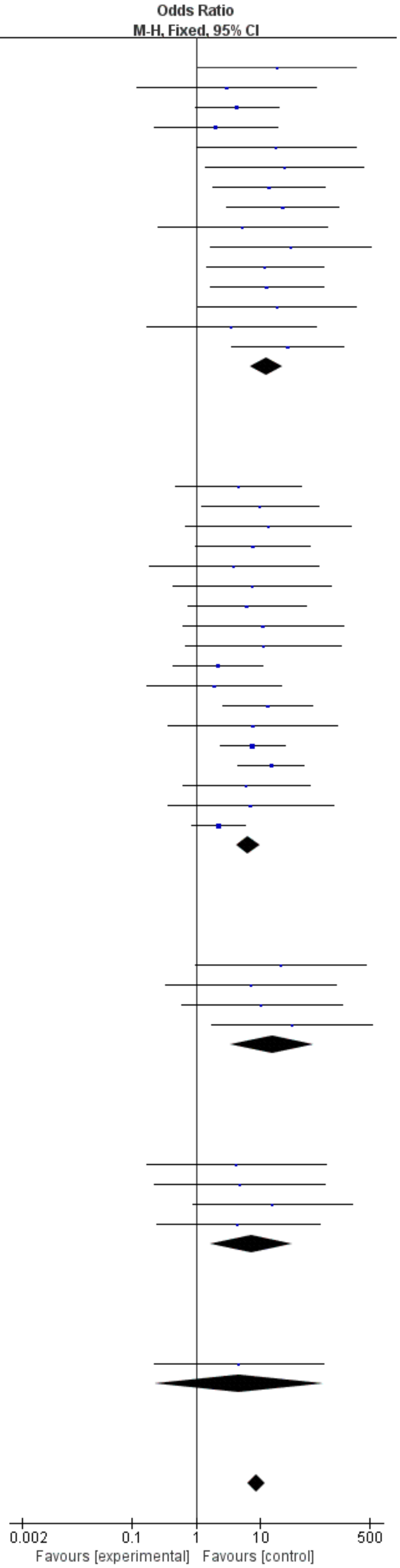


(L)

(M)

| Study or Subgroup | Experimental Events | Experimental Total | Control Events | Control Total | Weight | Odds Ratio M-H, Fixed, 95% CI |
|---|---|---|---|---|---|---|
| **7.33.1 East Asians** | | | | | | |
| 2007Shira G. Ziegler | 0 | 92 | 1 | 92 | 17.4% | 0.33 [0.01, 8.20] |
| 2008Usha Gutt | 0 | 184 | 1 | 92 | 23.2% | 0.17 [0.01, 4.10] |
| 2009Jun Mitsui | 2 | 534 | 1 | 544 | 11.5% | 2.04 [0.18, 22.58] |
| **Subtotal (95% CI)** | | **810** | | **728** | **52.1%** | **0.63 [0.15, 2.77]** |
| Total events | 2 | | 3 | | | |
| Heterogeneity: Chi² = 1.74, df = 2 (P = 0.42); I² = 0% | | | | | | |
| Test for overall effect: Z = 0.61 (P = 0.54) | | | | | | |
| **7.33.2 European/West Asians** | | | | | | |
| 2009 Kallirhoe Kalinderi | 0 | 172 | 2 | 132 | 32.8% | 0.15 [0.01, 3.18] |
| 2011Suzanne Lesage(1) | 1 | 1391 | 0 | 391 | 9.1% | 0.84 [0.03, 20.78] |
| **Subtotal (95% CI)** | | **1563** | | **523** | **41.9%** | **0.30 [0.04, 2.13]** |
| Total events | 1 | | 2 | | | |
| Heterogeneity: Chi² = 0.59, df = 1 (P = 0.44); I² = 0% | | | | | | |
| Test for overall effect: Z = 1.20 (P = 0.23) | | | | | | |
| **7.33.3 African** | | | | | | |
| 2011S. Lesage(2) | 1 | 194 | 0 | 177 | 6.0% | 2.75 [0.11, 67.99] |
| **Subtotal (95% CI)** | | **194** | | **177** | **6.0%** | **2.75 [0.11, 67.99]** |
| Total events | 1 | | 0 | | | |
| Heterogeneity: Not applicable | | | | | | |
| Test for overall effect: Z = 0.62 (P = 0.54) | | | | | | |
| **Total (95% CI)** | | **2567** | | **1428** | **100.0%** | **0.62 [0.22, 1.80]** |
| Total events | 4 | | 5 | | | |
| Heterogeneity: Chi² = 3.43, df = 5 (P = 0.63); I² = 0% | | | | | | |
| Test for overall effect: Z = 0.87 (P = 0.38) | | | | | | |
| Test for subgroup differences: Chi² = 1.35, df = 2 (P = 0.51), I² = 0% | | | | | | |

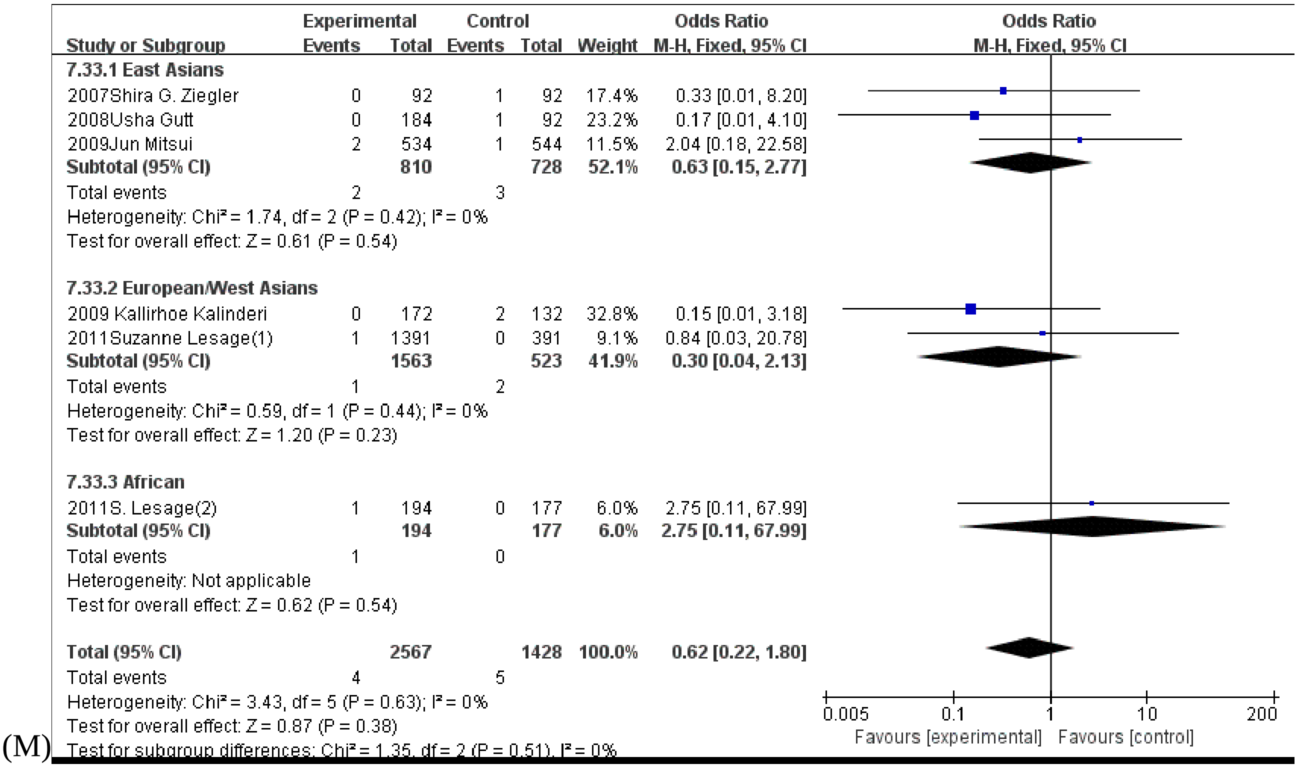


(N)

| Study or Subgroup | Experimental Events | Experimental Total | Control Events | Control Total | Weight | Odds Ratio M-H, Fixed, 95% CI |
|---|---|---|---|---|---|---|
| **7.37.1 European/West Asians** | | | | | | |
| 2009Juliane Neumann | 3 | 790 | 0 | 257 | 28.4% | 2.29 [0.12, 44.46] |
| 2011Suzanne Lesage(1) | 2 | 1391 | 0 | 391 | 29.5% | 1.41 [0.07, 29.40] |
| 2012K. R. Kumar | 1 | 360 | 0 | 348 | 19.2% | 2.91 [0.12, 71.63] |
| 2013Michael A. Nalls | 0 | 151 | 1 | 1962 | 8.1% | 4.32 [0.18, 106.39] |
| 2013Raquel Duran | 3 | 185 | 0 | 283 | 14.7% | 10.87 [0.56, 211.75] |
| **Subtotal (95% CI)** | | **2877** | | **3241** | **100.0%** | **3.58 [0.88, 14.50]** |
| Total events | 9 | | 1 | | | |
| Heterogeneity: Chi² = 1.02, df = 4 (P = 0.91); I² = 0% | | | | | | |
| Test for overall effect: Z = 1.78 (P = 0.07) | | | | | | |
| **Total (95% CI)** | | **2877** | | **3241** | **100.0%** | **3.58 [0.88, 14.50]** |
| Total events | 9 | | 1 | | | |
| Heterogeneity: Chi² = 1.02, df = 4 (P = 0.91); I² = 0% | | | | | | |
| Test for overall effect: Z = 1.78 (P = 0.07) | | | | | | |
| Test for subgroup differences: Not applicable | | | | | | |

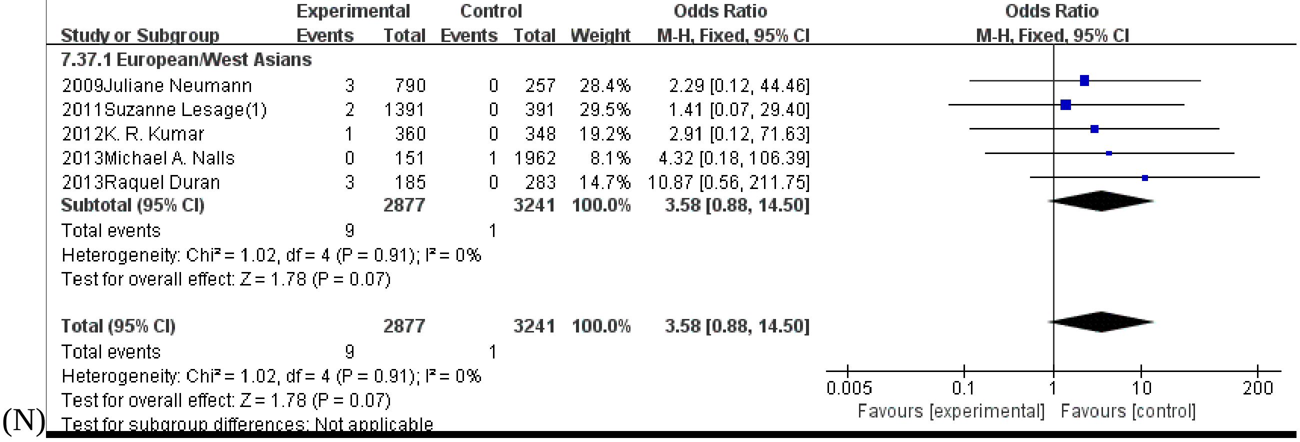


(O)

| Study or Subgroup | Experimental Events | Experimental Total | Control Events | Control Total | Weight | Odds Ratio M-H, Fixed, 95% CI |
|---|---|---|---|---|---|---|
| **7.41.1 East Asians** | | | | | | |
| 2007Shira G. Ziegler | 1 | 92 | 0 | 92 | 20.5% | 3.03 [0.12, 75.42] |
| 2008Usha Gutt | 1 | 184 | 1 | 92 | 55.3% | 0.50 [0.03, 8.04] |
| 2014Zhe Yu | 1 | 184 | 0 | 130 | 24.2% | 2.13 [0.09, 52.79] |
| **Subtotal (95% CI)** | | **460** | | **314** | **100.0%** | **1.41 [0.26, 7.57]** |
| Total events | 3 | | 1 | | | |
| Heterogeneity: Chi² = 0.82, df = 2 (P = 0.66); I² = 0% | | | | | | |
| Test for overall effect: Z = 0.40 (P = 0.69) | | | | | | |
| **Total (95% CI)** | | **460** | | **314** | **100.0%** | **1.41 [0.26, 7.57]** |
| Total events | 3 | | 1 | | | |
| Heterogeneity: Chi² = 0.82, df = 2 (P = 0.66); I² = 0% | | | | | | |
| Test for overall effect: Z = 0.40 (P = 0.69) | | | | | | |
| Test for subgroup differences: Not applicable | | | | | | |

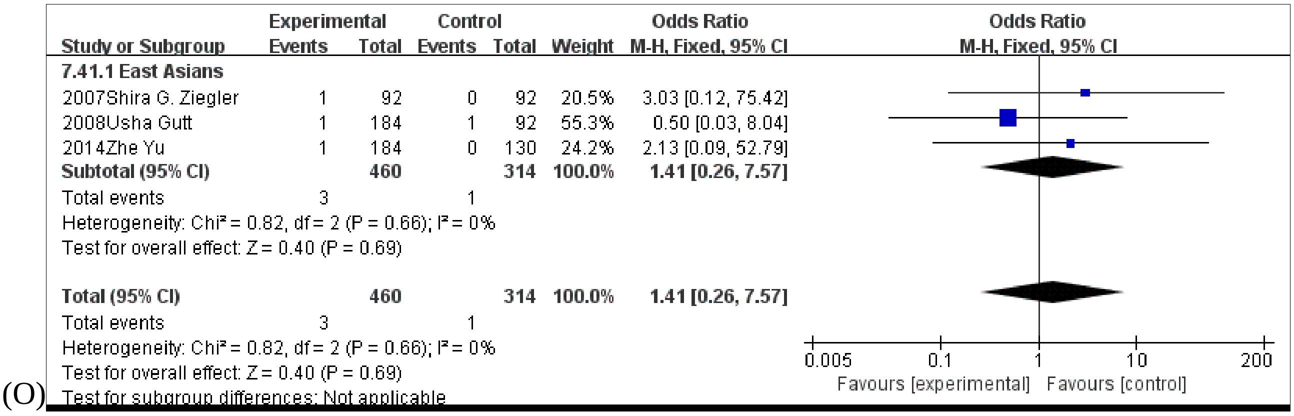

(P)

| Study or Subgroup | Experimental Events | Experimental Total | Control Events | Control Total | Weight | Odds Ratio M-H, Fixed, 95% CI |
|---|---|---|---|---|---|---|
| **7.44.1 East Asians** | | | | | | |
| 2007Yih-Ru Wu | 2 | 518 | 2 | 339 | 16.2% | 0.65 [0.09, 4.66] |
| 2009Jun Mitsui | 14 | 534 | 2 | 544 | 13.0% | 7.30 [1.65, 32.26] |
| 2011C.-L. Huang | 7 | 967 | 1 | 780 | 7.4% | 5.68 [0.70, 46.27] |
| 2014Yuanzhe Li | 1 | 147 | 1 | 100 | 8.0% | 0.68 [0.04, 10.97] |
| 2014Zhe Yu | 3 | 184 | 0 | 130 | 3.9% | 5.03 [0.26, 98.27] |
| **Subtotal (95% CI)** | | **2350** | | **1893** | **48.4%** | **3.56 [1.53, 8.29]** |
| Total events | 27 | | 6 | | | |
| Heterogeneity: Chi² = 5.36, df = 4 (P = 0.25); I² = 25% | | | | | | |
| Test for overall effect: Z = 2.94 (P = 0.003) | | | | | | |
| **7.44.2 European/West Asians** | | | | | | |
| 2009Juliane Neumann | 2 | 790 | 0 | 257 | 5.1% | 1.63 [0.08, 34.12] |
| 2011Nu´ ria Seto´ -Salvia | 1 | 225 | 0 | 186 | 3.7% | 2.49 [0.10, 61.54] |
| 2011Suzanne Lesage(1) | 2 | 1391 | 0 | 391 | 5.2% | 1.41 [0.07, 29.40] |
| 2012K. R. Kumar | 1 | 360 | 0 | 348 | 3.4% | 2.91 [0.12, 71.63] |
| 2013Michael A. Nalls | 0 | 151 | 3 | 1962 | 3.4% | 1.85 [0.10, 35.94] |
| 2013Raquel Duran | 3 | 185 | 0 | 283 | 2.6% | 10.87 [0.56, 211.75] |
| 2016David Crosiers | 1 | 266 | 0 | 536 | 2.2% | 6.06 [0.25, 149.31] |
| **Subtotal (95% CI)** | | **3368** | | **3963** | **25.6%** | **3.24 [1.03, 10.20]** |
| Total events | 10 | | 3 | | | |
| Heterogeneity: Chi² = 1.44, df = 6 (P = 0.96); I² = 0% | | | | | | |
| Test for overall effect: Z = 2.01 (P = 0.04) | | | | | | |
| **7.44.3 Hispanics** | | | | | | |
| 2010Adriana Vaz dos Santos | 1 | 110 | 0 | 155 | 2.8% | 4.26 [0.17, 105.56] |
| **Subtotal (95% CI)** | | **110** | | **155** | **2.8%** | **4.26 [0.17, 105.56]** |
| Total events | 1 | | 0 | | | |
| Heterogeneity: Not applicable | | | | | | |
| Test for overall effect: Z = 0.88 (P = 0.38) | | | | | | |
| **7.44.4 Mix** | | | | | | |
| 2007L.N. Clark | 1 | 100 | 0 | 94 | 3.4% | 2.85 [0.11, 70.81] |
| 2008W.C. Nichols | 4 | 450 | 1 | 359 | 7.4% | 3.21 [0.36, 28.85] |
| 2015Fabin Han | 1 | 225 | 1 | 110 | 9.0% | 0.49 [0.03, 7.85] |
| **Subtotal (95% CI)** | | **775** | | **563** | **19.8%** | **1.91 [0.45, 8.09]** |
| Total events | 6 | | 2 | | | |
| Heterogeneity: Chi² = 1.20, df = 2 (P = 0.55); I² = 0% | | | | | | |
| Test for overall effect: Z = 0.88 (P = 0.38) | | | | | | |
| **7.44.5 African** | | | | | | |
| 2011S. Lesage(2) | 2 | 194 | 0 | 177 | 3.5% | 4.61 [0.22, 96.69] |
| **Subtotal (95% CI)** | | **194** | | **177** | **3.5%** | **4.61 [0.22, 96.69]** |
| Total events | 2 | | 0 | | | |
| Heterogeneity: Not applicable | | | | | | |
| Test for overall effect: Z = 0.98 (P = 0.32) | | | | | | |
| **Total (95% CI)** | | **6797** | | **6751** | **100.0%** | **3.21 [1.77, 5.79]** |
| Total events | 46 | | 11 | | | |
| Heterogeneity: Chi² = 8.55, df = 16 (P = 0.93); I² = 0% | | | | | | |
| Test for overall effect: Z = 3.86 (P = 0.0001) | | | | | | |
| Test for subgroup differences: Chi² = 0.63, df = 4 (P = 0.96), I² = 0% | | | | | | |

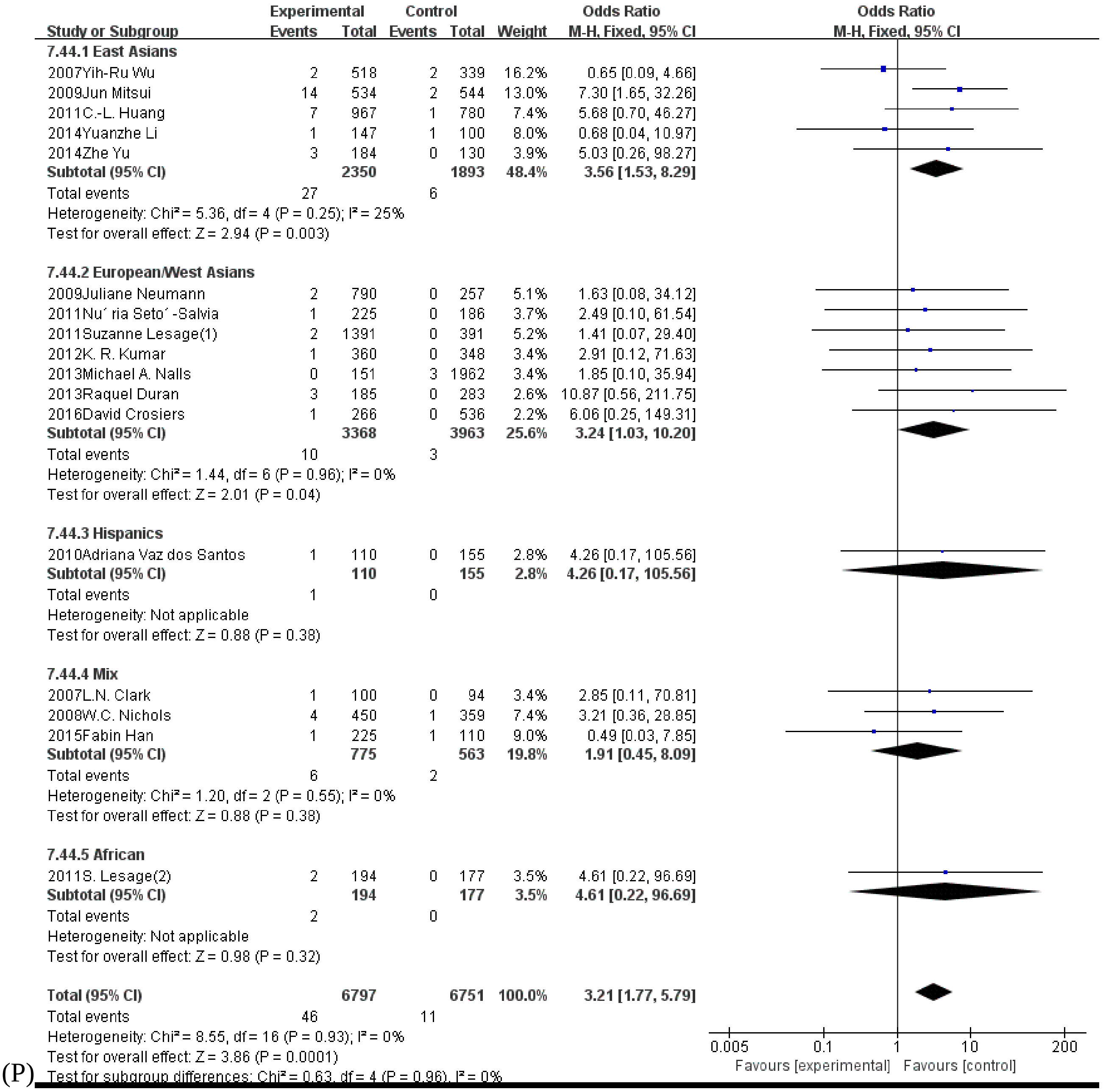


**Supplementary Figure 3**: Forest plots of the association between each *GBA* variant included and PD risks in non-AJ and by areas. (A)-(P) were individually responsive to the variants IVS2+1G＞A, R120W, R131C, R163Q, H255Q, E326K, T369M, N370S, E388K, D409H, D443N, L444P, V460L/M/V, R463C, Q497R and RecNciI.
