# Supplementary material for: Integrated Genetic Analysis of Racial Differences of Common GBA Variants in Parkinson's Disease: A Meta-Analysis
Source: Front Mol Neurosci. 2018 Feb 15;11:43. doi: 10.3389/fnmol.2018.00043 (PMC5829555; doi:10.3389/fnmol.2018.00043)
Supplement: Supplementary file 8 [file Image4.PDF]

Supplementary Material

Integrated genetic analysis of racial differences of common GBA variants in parkinson's disease: a Meta-analysis

Yuan Zhang[1 †], Li Shu[1 †], Qiying Sun[2,3,4], Xun Zhou[1], Hongxu Pan[1], Jifeng Guo[1, 3,4], Beisha Tang[1, 3,4,5*]

[†] These authors have contributed equally to this work and are co-first authors.

[*] Correspondence: Beisha Tang bstang7398@163.com

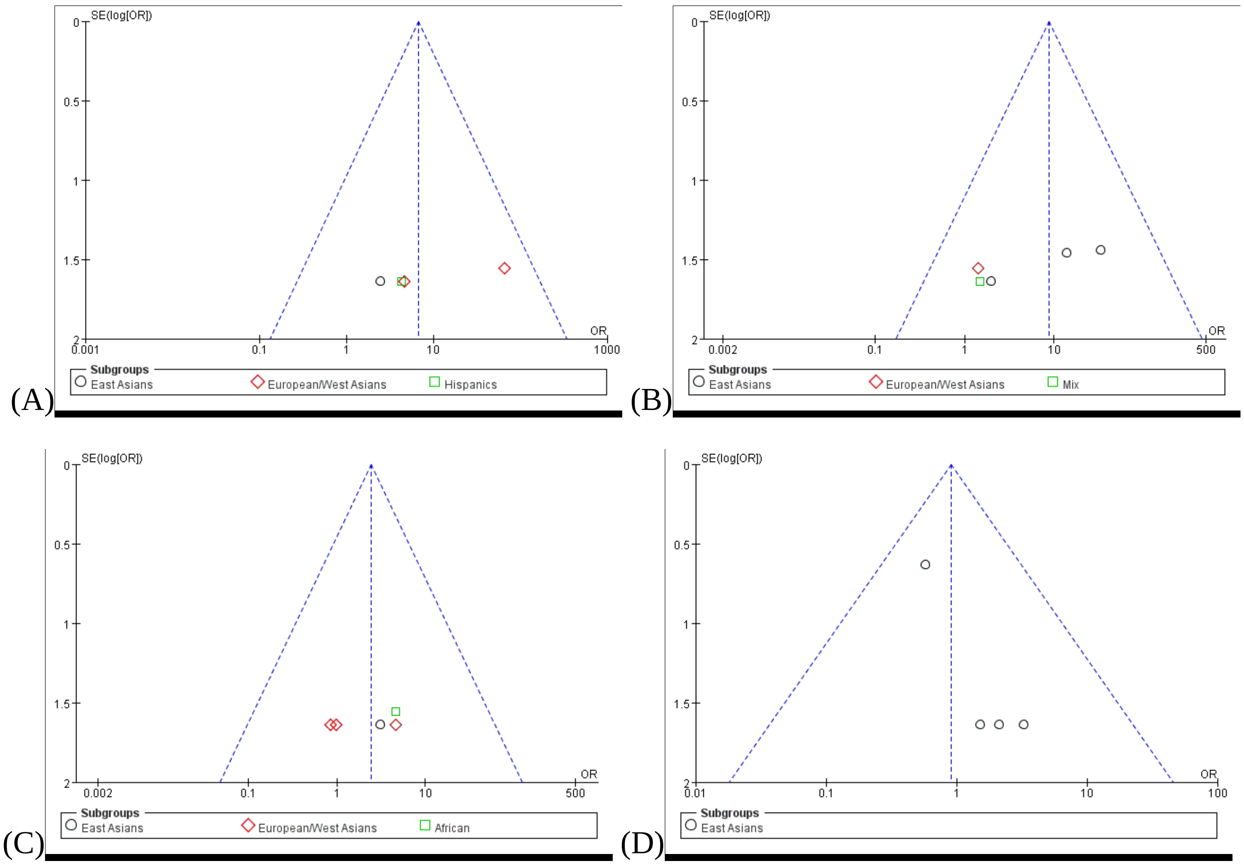

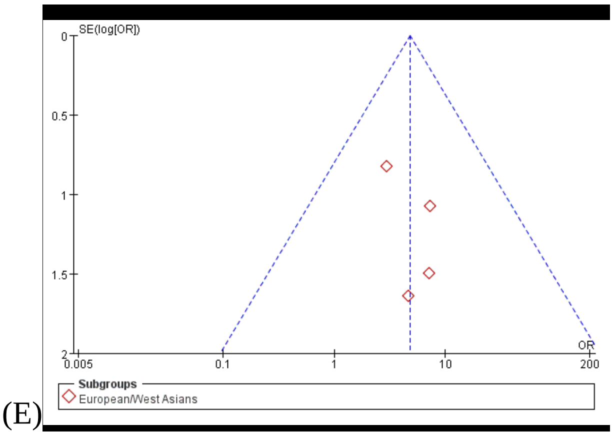
SE(log[OR])
0
0.5
1
1.5
2
0.005
0.1
1
10
200
OR
Subgroups
European/West Asians

(E)

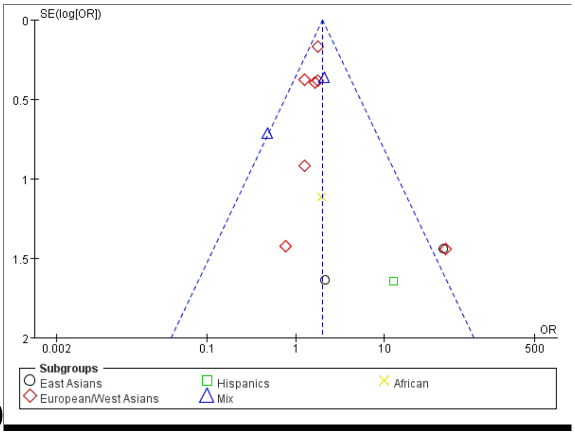
SE(log[OR])
0
0.5
1
1.5
2
0.002
0.1
1
10
500
OR
Subgroups
East Asians
Hispanics
African
European/West Asians
Mix

(F)

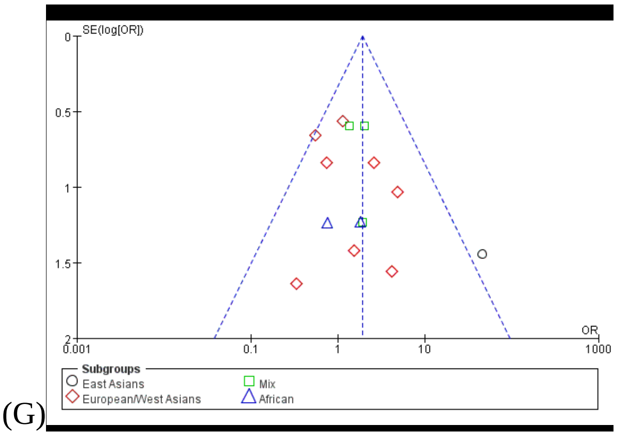
SE(log[OR])
0
0.5
1
1.5
2
0.001
0.1
1
10
1000
OR
Subgroups
East Asians
Mix
European/West Asians
African

(G)

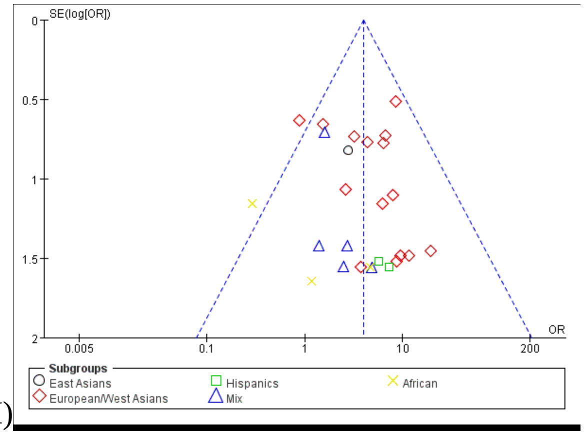
SE(log[OR])
0
0.5
1
1.5
2
0.005
0.1
1
10
200
OR
Subgroups
East Asians
Hispanics
African
European/West Asians
Mix

(H)

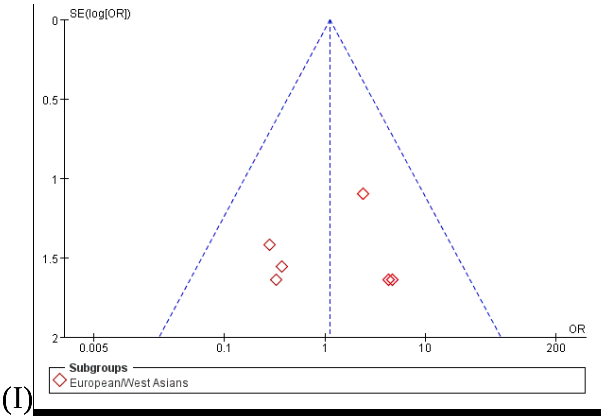
SE(log[OR])
0
0.5
1
1.5
2
0.005
0.1
1
10
200
OR
Subgroups
European/West Asians

(I)

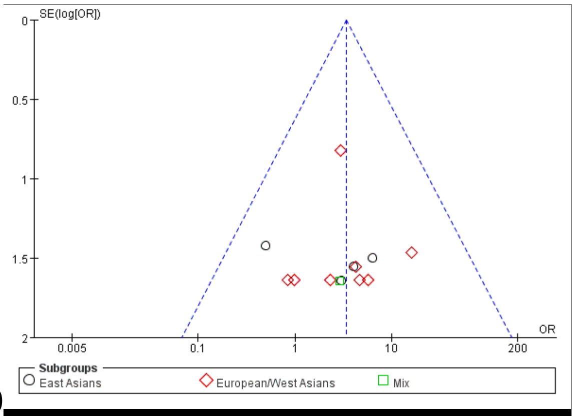
SE(log[OR])
0
0.5
1
1.5
2
0.005
0.1
1
10
200
OR
Subgroups
East Asians
European/West Asians
Mix

(J)

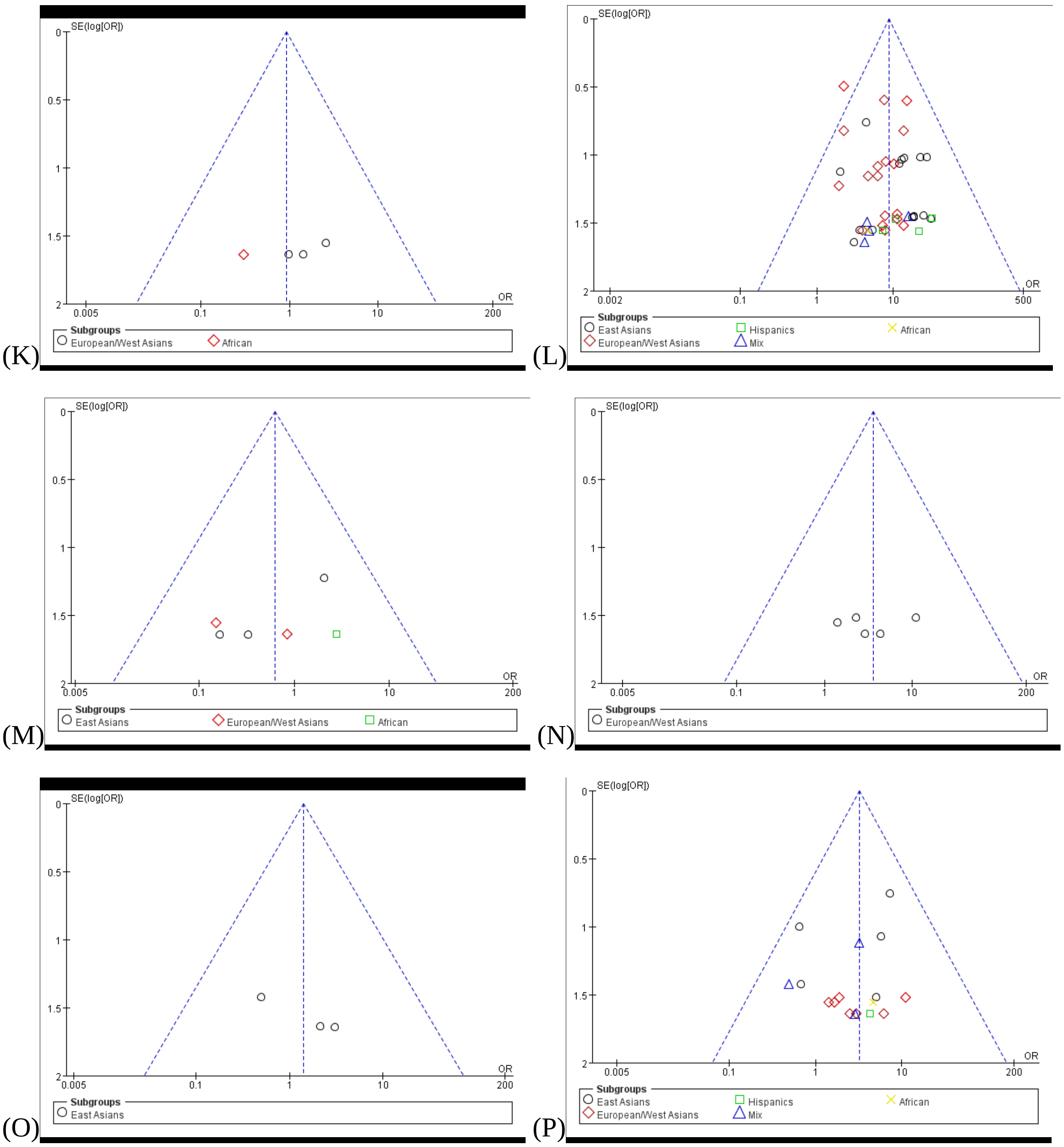

Supplementary Figure 4: Funnel plots of the association between each *GBA* variant and PD risks in non-AJ and by areas. (A)-(P) were individually responsive to the variants IVS2+1G>A, R120W, R131C, R163Q, H255Q, E326K, T369M, N370S, E388K, D409H, D443N, L444P, V460L/M/V, R463C, Q497R and RecNciI.
